# Supplementary material for: Private sector delivery of quality care for maternal, newborn and child health in low-income and middle-income countries: a mixed-methods systematic review protocol
Source: BMJ Open. 2020 Feb 17;10(2):e033141. doi: 10.1136/bmjopen-2019-033141 (PMC7045217; doi:10.1136/bmjopen-2019-033141)
Supplement: Supplementary data [file bmjopen-2019-033141supp001.pdf]

Background information										
Reviewer's initials	Number assigned to each article/study (e.g., 001, 002)	First author's surname	Full name of the study or article	Year of publication	State the exclusion criteria for the study, or state "none."<br>*If study meets any of the exclusion criteria, end data extraction.*	Does this study meet all inclusion criteria? Check PICOTS, language, year, and inclusion of quality. If all criteria are met, state "all."	Type of publication	If PubTyp = 5, give details.	Detail any other publications linked to the study (e.g. papers describing methods, additional analyses)	Language of publication
**Reviewer**	**Study ID**	**Author**	**Study Name**	**Publication Date**	**Exclusion Criteria**	**Inclusion Criteria**	**Publication Type$1**	**Publication Type$2**	**Other Publications**	**Language of Publication**

				Intervention background information									
List all countries in which the study was conducted	Country(ies) income group	World region(s)	State the primary or main objective(s)/aim(s) of the study	Give (Month/Year) or (Year)	Give (Month/Year) or (Year)	Intervention duration in months	Which agency or agencies implemented the intervention?	Details	At which geographical level did the study occur?	Give specific detail for previous response (e.g. Bongo district (17 villages, 6,158 inhabitants))	What was the main identifying characteristic of the population targeted?	Give specific detail for previous response (e.g., women in the third trimester). Use author's words (with quotation marks)	Provide the number enrolled in the intervention population
**Country**	**Country Income Group**	**World Region**	**Study Objective**	**Intervention Start Date**	**Intervention End Date**	**Intervention Duration**	**Implementing Agency$1**	**Implementing Agency$2**	**Geographic Level$1**	**Geographic Level$2**	**Study Population$1**	**Study Population$2**	**Study Population$3**

Provide the number enrolled in the control population **Study Population$4**	Provide the mean age of the intervention population **Study Population$5**	Provide the mean age of the control population **Study Population$6**	Provide the mean age of the combined population (intervention and control) **Study Population$7**	Copy all text describing the intervention; ensure comprehensive extraction of intervention characteristics **Intervention Description**	Did the study examine a single intervention or multiple interventions? **Intervention Quantity**	Was the intervention focused on supply-side or demand-side factors? **Intervention Supply--Demand**	Were women during pregnancy, childbirth and/or postpartum intervention recipients? (Unless it is explicit, it is a no). **Intervention Recipient$1**	Was a subgroup of women during pregnancy, childbirth, postpartum (e.g., women with HIV) the intervention recipient? (Unless it is explicit, it is a no). **Intervention Recipient$2**	Were women defined more generally (e.g., all women, all women of reproductive age) intervention recipients? (Unless it is explicit, it is a no). **Intervention Recipient$3**	Were newborns intervention recipients? (Unless it is explicit, it is a no). **Intervention Recipient$4**	Were children intervention recipients? (Unless it is explicit, it is a no). **Intervention Recipient$5**	Were health care providers intervention recipients? (Unless it is explicit, it is a no). **Intervention Recipient$6**

Intervention details										
Were any others intervention recipients? (Unless it is explicit, it is a no).	Give specific detail for previous response (Intervention recipient code: “author’s description”)	Was on-site support for quality improvement introduced/changed as part of the intervention? (Unless it is explicit, it is a no).	Were data systems introduced/changed as part of the intervention? (Unless it is explicit, it is a no).	Were learning systems introduced/changed as part of the intervention? (Unless it is explicit, it is a no).	Was program management selected/changed as part of the intervention? (Unless it is explicit, it is a no).	Was advocacy introduced/ provided as part of the intervention? (Unless it is explicit, it is a no).	Was policy and strategy development introduced/provided as part of the intervention? (Unless it is explicit, it is a no).	Were other systems introduced or changed as part of the intervention?	Give specific details for previous responses (e.g., coaching, training, electronic data systems, community involvement) (Nature of intervention code: “author’s description”)	Did the text explicitly use one of the following quality terms: ‘quality of care,’ ‘quality care,’ ‘quality assessment,’ ‘quality improvement,’ ‘clinical quality,’ ‘perceived quality,’ or another ‘quality’ term?
**Intervention Recipient$7**	**Intervention Recipient$8**	**Nature of Intervention$1**	**Nature of Intervention$2**	**Nature of Intervention$3**	**Nature of Intervention$4**	**Nature of Intervention$5**	**Nature of Intervention$6**	**Nature of Intervention$7**	**Nature of Intervention$8**	**Reference to Quality$1**

Did the text explicitly define ‘quality of care,’ ‘quality care,’ ‘quality assessment,’ ‘quality improvement’ or another ‘quality’ term?	If Quality$2 = 1, how did the text define/refer to ‘quality of care,’ ‘quality care,’ ‘quality assessment,’ ‘quality improvement’ or another ‘quality’ term?’ Give exact text in quotation marks.	Which of the six dimensions of quality did the study address?	If Quality$4 = 7, specify the dimensions (in alphabetical order).	Did the study report on the outcome ‘maternal morbidity?’	In the author’s words, include the text describing the outcome ‘maternal morbidity.’	Report all outcome data for ‘maternal morbidity.’	Did the study report on the outcome ‘maternal mortality?’	In the author’s words, include the text describing the outcome ‘maternal mortality.’	Report all outcome data for ‘maternal mortality.’	Did the study report on the outcome ‘newborn morbidity?’	In the author’s words, include the text describing the outcome ‘newborn morbidity.’	Report all outcome data for ‘newborn morbidity.’
**Reference to Quality$2**	**Reference to Quality$3**	**Reference to Quality$4**	**Reference to Quality$5**	**Critical Outcome$1A**	**Critical Outcome$1B**	**Critical Outcome$1C**	**Critical Outcome$2A**	**Critical Outcome$2B**	**Critical Outcome$2C**	**Critical Outcome$3A**	**Critical Outcome$3B**	**Critical Outcome$3C**

Outcomes													
Did the study report on the outcome ‘newborn mortality?’	In the author’s words, include the text describing the outcome ‘newborn mortality.’	Report all outcome data for ‘newborn mortality.’	Did the study report on the outcome ‘child morbidity?’	In the author’s words, include the text describing the outcome ‘child morbidity.’	Report all outcome data for ‘child morbidity.’	Did the study report on the outcome ‘child mortality?’	In the author’s words, include the text describing the outcome ‘child mortality.’	Report all outcome data for ‘child mortality.’	Did the study report on the outcome ‘quality of care?’	In the author’s words, include the text describing the outcome ‘quality of care.’	Report all outcome data for ‘quality of care.’	Did the study report on the outcome ‘experience of care, including respectful care?’	In the author’s words, include the text describing the outcome ‘experience of care, including respectful care.’
**Critical Outcome$4A**	**Critical Outcome$4B**	**Critical Outcome$4C**	**Critical Outcome$5A**	**Critical Outcome$5B**	**Critical Outcome$5C**	**Critical Outcome$6A**	**Critical Outcome$6B**	**Critical Outcome$6C**	**Critical Outcome$7A**	**Critical Outcome$7B**	**Critical Outcome$7C**	**Critical Outcome$8A**	**Critical Outcome$8B**

Report all outcome data for ‘experience of care, including respectful care.’	Did the study report on the outcome ‘service utilization and efficiency?’	In the author’s words, include the text describing the outcome ‘service utilization and efficiency.’	Report all outcome data for ‘service utilization and efficiency.’	Did the study report on the outcome 'infant and/or child growth?'	In the author’s words, include the text describing the outcome ‘infant and/or child growth.’	Report all outcome data for ‘infant and/or child growth.’	What other outcomes were described or measured? Give details. Include all outcomes relating to stillbirth, childbirth, health system outcomes, and women's views and satisfaction.	Was the intervention evaluated?	What type of evaluation was done?	Give details with quotations	What was the design of the study?	Give details with quotations
**Critical Outcome$8C**	**Critical Outcome$9A**	**Critical Outcome$9B**	**Critical Outcome$9C**	**Secondary Outcome$1A**	**Secondary Outcome$1B**	**Secondary Outcome$1C**	**Additional Outcomes**	**Evaluated**	**Type of Evaluation$1**	**Type of Evaluation$2**	**Study Type$1**	**Study Type$2**

Evaluation/study details										
Control or comparator group included?	If Control/ Comparator$1 = 1, give details	What type of data were presented in the evaluation findings/results?	Were the data longitudinal?	What claims are made for the effects of the intervention on the outcome/s relevant to quality of maternal, newborn, and child health care services?	Give details. Use author's words (with quotation marks)	Copy any text about barriers/enablers to the intervention, factors influencing its delivery.	Extract all data on the cost of the intervention and cost compared to outcomes.	Extract all data on the cost effectiveness of the intervention.	Did the study discuss or propose any mechanisms for engaging the private sector in planning, delivering, and demonstrating accountability for quality maternal, newborn, and child health services? If so, give details and use the author's words (with quotation marks).	In what context was the study conducted? Give details (e.g. regulatory environment, collaborative service delivery models).
**Control/ Comparator$1**	**Control/ Comparator$2**	**Data Type**	**Longitudinal Data**	**Intervention Claim$1**	**Intervention Claim$2**	**Strategy Effectiveness**	**Cost Data**	**Cost Effectiveness Data**	**Mechanisms**	**Context**

													Qu
Include any notes you think are relevant	Are the individuals selected to participate in the study likely to be representative of the target population?	What percentage of selected individuals agreed to participate?	Rate this section (see dictionary)	Was the study described as randomized? (Check Study Type$1) If NO, go to Confounders $1.	If YES, was the method of randomization described? (see dictionary)	If YES, was the method appropriate? (see dictionary)	Rate this section (see dictionary)	Were there important differences between groups prior to the intervention?	If YES, indicate the percentage of relevant confounders that were controlled (either in the design (e.g. stratification, matching) or analysis)?	Rate this section (see dictionary)	Was (were) the outcome assessor(s) aware of the information or exposure status of participants?	Were the study participants aware of the research question?	Rate this section (see dictionary)
**Notes**	**Selection Bias$1**	**Selection Bias$2**	**Selection Bias$3**	**Study Design$1**	**Study Design$2**	**Study Design$3**	**Study Design$4**	**Confounders$1**	**Confounders$2**	**Confounders$3**	**Blinding$1**	**Blinding$2**	**Blinding$3**

uality assessment (for quantitative studies): Reviewer 1

Were data collection tools shown to be valid? **Data Collection Methods$1**	Were data collection tools shown to be reliable? **Data Collection Methods$2**	Rate this section (see dictionary) **Data Collection Methods$3**	Were withdrawals and dropouts reported in terms of numbers and/or reasons per group? **Withdrawals and Dropouts$1**	Indicate the percentage of participants completing the study. (If the percentage differs by groups, record the lowest.) **Withdrawals and Dropouts$2**	Rate this section (see dictionary) **Withdrawals and Dropouts$3**	What percentage of participants received the allocated intervention or exposure of interest? **Intervention Integrity$1**	Was the consistency of the intervention measured? **Intervention Integrity$2**	Is it likely that the subjects received an unintended intervention (contamination or co-intervention) that may influence the results? **Intervention Integrity$3**	Indicate the unit of allocation. **Analyses$1**	Indicate the unit of analysis. **Analyses$2**	Are the statistical methods appropriate for the study design? **Analyses$3**	Is the analysis performed by intervention allocation status (i.e. intention to treat) rather than the actual intervention received? **Analyses$4**	Based on the final section ratings for selection bias, study design, confounders, blinding, data collection methods, and withdrawals and dropouts, what is the global rating for this paper? **Global Rating$1**

With both reviewers discussing the ratings, is there a discrepancy between the two reviewers with respect to the following sections: for selection bias, study design, confounders, blinding, data collection methods, and withdrawals and dropouts	If YES, indicate the reason for the discrepancy.	Final decision of both reviewers	Are the individuals selected to participate in the study likely to be representative of the target population?	What percentage of selected individuals agreed to participate?	Rate this section (see dictionary)	Was the study described as randomized? (Check Study Type$1) If NO, go to Confounders $4.	If YES, was the method of randomization described? (see dictionary)	If YES, was the method appropriate? (see dictionary)	Rate this section (see dictionary)	Were there important differences between groups prior to the intervention?	If YES, indicate the percentage of relevant confounders that were controlled (either in the design (e.g. stratification, matching) or analysis)?	Rate this section (see dictionary)
**Global Rating$2**	**Global Rating$3**	**Global Rating$4**	**Selection Bias$4**	**Selection Bias$5**	**Selection Bias$6**	**Study Design$5**	**Study Design$6**	**Study Design$7**	**Study Design$8**	**Confounders$4**	**Confounders$5**	**Confounders$6**

Quality assessment (for quantitative studies): Reviewer 2

Was (were) the outcome assessor(s) aware of the information or exposure status of participants? **Blinding$4**	Were the study participants aware of the research question? **Blinding$5**	Rate this section (see dictionary) **Blinding$6**	Were data collection tools shown to be valid? **Data Collection Methods$4**	Were data collection tools shown to be reliable? **Data Collection Methods$5**	Rate this section (see dictionary) **Data Collection Methods$6**	Were withdrawals and dropouts reported in terms of numbers and/or reasons per group? **Withdrawals and Dropouts$4**	Indicate the percentage of participants completing the study. (If the percentage differs by groups, record the lowest.) **Withdrawals and Dropouts$5**	Rate this section (see dictionary) **Withdrawals and Dropouts$6**	What percentage of participants received the allocated intervention or exposure of interest? **Intervention Integrity$4**	Was the consistency of the intervention measured? **Intervention Integrity$5**	Is it likely that the subjects received an unintended intervention (contamination or co-intervention) that may influence the results? **Intervention Integrity$6**	Indicate the unit of allocation. **Analyses$5**	Indicate the unit of analysis. **Analyses$6**

Are the statistical methods appropriate for the study design? Analyses$7	Is the analysis performed by intervention allocation status (i.e. intention to treat) rather than the actual intervention received? Analyses$8	Based on the final section ratings for selection bias, study design, confounders, blinding, data collection methods, and withdrawals and dropouts, what is the global rating for this paper? Global Rating$5	Does the study have a clear statement of, and rationale for, its research question/aims/ purposes? Scope and Purpose$1	Is the study thoroughly contextualized by existing literature? Scope and Purpose$2	Rate this section Scope and Purpose$3	Is the method/design apparent and consistent with the research intent? Design$1	Is the data collection strategy apparent and appropriate? Design$2	Rate this section Design$3	Are the sampling and sampling method appropriate? Sampling Strategy$1	Rate this section Sampling Strategy$2	Is the analytical approach appropriate? Analysis$1	Rate this section Analysis$2

Quality assessment (for qualitative studies): Reviewer 1

Is the context described and taken account of in the interpretation?	Is a clear audit trail given?	Are data used to support interpretation?	Rate this section	Is researcher reflexivity demonstrated?	Rate this section	Is there demonstration of sensitivity to ethical concerns?	Rate this section	Are relevance and transferability evident?	Rate this section	Based on the final section ratings for scope and purpose, design, sampling strategy, analysis, interpretation, reflexivity, ethical dimensions, and relevance and transferability, what is the global rating for this paper?
**Interpretation$1**	**Interpretation$2**	**Interpretation$3**	**Interpretation$4**	**Reflexivity$1**	**Reflexivity$2**	**Ethical Dimensions$1**	**Ethical Dimensions$2**	**Relevance and Transferability$1**	**Relevance and Transferability$2**	**Global Rating$1**

													Quality
With both reviewers discussing the ratings, is there a discrepancy between the two reviewers with respect to the following sections: scope and purpose, design, sampling strategy, analysis, interpretation, reflexivity, ethical dimensions, and relevance and transferability<br>**Global Rating$2**	If YES, indicate the reason for the discrepancy.<br>**Global Rating$3**	Final decision of both reviewers<br>**Global Rating$4**	Does the study have a clear statement of, and rationale for, its research question/aims/ purposes?<br>**Scope and Purpose$4**	Is the study thoroughly contextualized by existing literature?<br>**Scope and Purpose$5**	Rate this section<br>**Scope and Purpose$6**	Is the method/design apparent and consistent with the research intent?<br>**Design$4**	Is the data collection strategy apparent and appropriate?<br>**Design$5**	Rate this section<br>**Design$6**	Are the sampling and sampling method appropriate?<br>**Sampling Strategy$3**	Rate this section<br>**Sampling Strategy$4**	Is the analytical approach appropriate?<br>**Analysis$3**	Rate this section<br>**Analysis$4**	

assessment (for qualitative studies): Reviewer 2

Is the context described and taken account of in the interpretation?	Is a clear audit trail given?	Are data used to support interpretation?	Rate this section	Is researcher reflexivity demonstrated?	Rate this section	Is there demonstration of sensitivity to ethical concerns?	Rate this section	Are relevance and transferability evident?	Rate this section	Based on the final section ratings for scope and purpose, design, sampling strategy, analysis, interpretation, reflexivity, ethical dimensions, and relevance and transferability, what is the global rating for this paper?
**Interpretation$5**	**Interpretation$6**	**Interpretation$7**	**Interpretation$8**	**Reflexivity$3**	**Reflexivity$4**	**Ethical Dimensions$3**	**Ethical Dimensions$4**	**Relevance and Transferability$3**	**Relevance and Transferability$4**	**Global Rating$5**
