# Supplementary material for: Private sector delivery of quality care for maternal, newborn and child health in low-income and middle-income countries: a mixed-methods systematic review protocol
Source: BMJ Open. 2020 Feb 17;10(2):e033141. doi: 10.1136/bmjopen-2019-033141 (PMC7045217; doi:10.1136/bmjopen-2019-033141)
Supplement: Supplementary data [file bmjopen-2019-033141supp002.pdf]

Annex B: Codebook

Variable	Question and instruction	Response options
Background information		
Reviewer	State your initials	SL<br>BM
Study ID	Number assigned to each article/study (e.g., 001, 002)	#
Author	First author's surname	Text
Study Name	Full name of the study or article	Text
Publication Date	Year of publication	#
Exclusion Criteria	State the exclusion criteria for the study, or state "none."<br><br>*If study meets any of the exclusion criteria, end data extraction.*	Text
Inclusion Criteria	Does this study meet all inclusion criteria? Check PICOTS, language, year, and inclusion of quality. If all criteria are met, state "all."<br><br>*If all of the inclusion criteria are not met, return to "exclusion criteria."*	All
Publication Type$1	Type of publication	1 = Peer-reviewed journal article<br>2 = Non-peer-reviewed journal article<br>3 = Report<br>4 = Book or book chapter<br>5 = Other
Publication Type$2	If PubTyp = 5, give details.	Text<br>99 = Not applicable
Other Publications	Detail any other publications linked to the study (e.g. papers describing methods, additional analyses)	Text
Language of Publication		1 = English<br>2 = French<br>3 = German<br>4 = Italian<br>5 = Other
Country	List all countries in which the study was conducted	Text
Country Income Group[1]	Country income group	1 = Low<br>2 = Lower-middle<br>3 = Upper-middle<br>4 = Multiple
World Region[2]	World region(s)	1 = Africa

[1] World Bank Atlas method groupings of countries by income: https://datahelpdesk.worldbank.org/knowledgebase/articles/906519

[2] WHO region groupings: Africa, Asia, Europe, Latin America and the Caribbean, Northern America, Oceania: https://unstats.un.org/unsd/methodology/m49/

Lattof SR, Maliqi B. BMJ Open 2023; 10:e033141. doi: 10.1136/bmjopen-2019-033141

Variable	Question and instruction	Response options
		2 = Asia<br>3 = Europe<br>4 = Latin America and the Caribbean<br>5 = Northern America<br>6 = Oceania<br>7 = Multiple
Study Objective	State the primary or main objective(s) or aim(s) of the study	Text
Intervention background information		
Intervention Start Date	Give (Month/Year) or (Year)<br><br>[Give start date of implementation rather than preliminary work]	(MM/YYYY) or (YYYY)<br>98 = Unclear/not specified
Intervention End Date	Give (Month/Year) or (Year)	(MM/YYYY) or (YYYY)<br>Ongoing= Ongoing at time of publication<br>98= Unclear/not specified
Intervention Duration	Duration of the intervention in months. If not straightforward, give details.	Text<br>98 = Unclear/not specified
Implementing Agency$1	Which type of agency or agencies implemented the intervention?	1 = Domestic government (in the case of a public-private partnership)<br>2 = Domestic non-profit organization (e.g., charity, civil society organization)<br>3 = Domestic for-profit organization<br>4 = International non-governmental organization (NGO)<br>5 = International for-profit organization<br>6 = Other international institution<br>7 = Multiple answers from list<br>8 = Other<br>98 = Unclear/not specified
Implementing Agency$2	If Agency$1 = 1-8, give details. (e.g., international NGO “Health Unlimited”…)	Text<br>99 = Not applicable
Geographic Level$1	At which geographical level did the study occur?	1 = National<br>2 = Sub-national (e.g., region, state, county, district, whole city)<br>3 = Local (e.g., village, neighborhood)<br>4 = Health facility<br>5 = Other<br>98 = Unclear/not specified
Geographic Level$2	Give specific detail for previous response (e.g. Bongo district (17 villages, 6,158 inhabitants)). Provide name if only one location unit.	Text<br>99 = if GeoLvl$1 = 98

Variable	Question and instruction	Response options
Study Population$1	What was the main identifying characteristic of the population the study targeted?	1 = Pregnant women<br>2 = Women during childbirth<br>3 = Mothers postpartum<br>4 = Newborns<br>5 = Children<br>6 = Health care providers<br>7 = Multiple answers from list<br>8 = Other, specify<br>98 = Unclear/not specified<br>99 = Not applicable
Study Population$2	Give specific detail for previous response (e.g., women in the third trimester). Use author's words (with quotation marks).	Text
Study Population$3	Provide the number enrolled in the intervention population.	#<br>98 = Unclear/not specified
Study Population$4	Provide the number enrolled in the control population, if applicable.	#<br>98 = Unclear/not specified<br>99 = No control population
Study Population$5	Provide the mean age of the intervention population.	#<br>98 = Unclear/not specified
Study Population$6	Provide the mean age of the control population, if applicable.	#<br>98 = Unclear/not specified<br>99 = No control population
Study Population$7	Provide the mean age of the combined population (intervention and control), if applicable.	#<br>98 = Unclear/not specified<br>99 = No control population
Intervention details		
Intervention Description	Copy all text describing the intervention; ensure comprehensive extraction of intervention characteristics.	Text
Intervention Quantity	Did the study examine a single intervention or multiple interventions?	1 = Single intervention<br>2 = Multiple interventions<br>98 = Unclear/not specified
Intervention Supply – Demand	Was the intervention focused on supply-side or demand-side factors?	1 = Supply side (e.g., staff training, change in types of staff, service environment, provider communication skills)<br>2 = Demand-side (e.g., education, peer encouragement for service use)<br>3 = Both<br>98 = Unclear/not specified
Intervention Recipient$1	Were women during pregnancy, childbirth and/or postpartum intervention recipients?<br>(Unless it is explicit, it is a no).	1 = Indirect<br>2 = Direct<br>3 = Not a recipient
Intervention Recipient$2	Was a subgroup of women during pregnancy, childbirth, postpartum	1 = Indirect<br>2 = Direct

Variable	Question and instruction	Response options
	(e.g., women who experience birth complications, women with HIV) the intervention recipient? (Unless it is explicit, it is a no).	3 = Not a recipient
Intervention Recipient$3	Were women defined more generally (e.g., all women, all women of reproductive age) intervention recipients? (Unless it is explicit, it is a no).	1 = Indirect<br>2 = Direct<br>3 = Not a recipient
Intervention Recipient$4	Were newborns intervention recipients? (Unless it is explicit, it is a no).	1 = Indirect<br>2 = Direct<br>3 = Not a recipient
Intervention Recipient$5	Were children intervention recipients? (Unless it is explicit, it is a no).	1 = Indirect<br>2 = Direct<br>3 = Not a recipient
Intervention Recipient$6	Were health care providers intervention recipients? (Unless it is explicit, it is a no).	1 = Indirect<br>2 = Direct<br>3 = Not a recipient
Intervention Recipient$7	Were any others intervention recipients? (Unless it is explicit, it is a no).	1 = Indirect<br>2 = Direct<br>3 = Not a recipient
Intervention Recipient$8	Give specific detail for previous response (Intervention recipient code: "author's description")	Text<br>99 = If Recipient$1-Recipient$7 = 3
Nature of Intervention$1	Was on-site support for quality improvement introduced/changed as part of the intervention? (Unless it is explicit, it is a no).	0 = No<br>1 = Yes
Nature of Intervention$2	Were data systems introduced/changed as part of the intervention? (Unless it is explicit, it is a no).	0 = No<br>1 = Yes
Nature of Intervention$3	Were learning systems introduced/changed as part of the intervention? (Unless it is explicit, it is a no).	0 = No<br>1 = Yes
Nature of Intervention$4	Was a program management selected/changed as part of the intervention? (Unless it is explicit, it is a no).	0 = No<br>1 = Yes
Nature of Intervention$5	Was advocacy introduced/ provided as part of the intervention? (Unless it is explicit, it is a no).	0 = No<br>1 = Yes
Nature of Intervention$6	Was policy and strategy development introduced/provided as part of the intervention? (Unless it is explicit, it is a no).	0 = No<br>1 = Yes
Nature of	Were other systems introduced or	0 = No

Variable	Question and instruction	Response options
Intervention$7	changed as part of the intervention?	1 = Yes
Nature of Intervention$8	Give specific details for previous responses (e.g., coaching, training, electronic data systems, community involvement)<br>(Nature of intervention code: "author's description")	Text
Reference to Quality$1	Did the text explicitly use one of the following quality terms: 'quality of care,' 'quality care,' 'quality assessment,' 'quality improvement,' 'clinical quality,' 'perceived quality,' or another 'quality' term?	0 = No<br>1 = Yes, quality of care or quality care<br>2 = Yes, quality assessment<br>3 = Yes, quality improvement<br>4 = Yes, clinical quality<br>5 = Yes, perceived quality<br>6 = Yes, multiple quality terms<br>7 = Yes, another quality term [specify in Quality$3]
Reference to Quality$2	Did the text explicitly define 'quality of care,' 'quality care,' 'quality assessment,' 'quality improvement,' 'clinical quality,' 'perceived quality,' or another 'quality' term?	0 = No<br>1 = Yes
Reference to Quality$3	If Quality$2 = 1, how did the text define/refer to 'quality of care,' 'quality care,' 'quality assessment,' 'quality improvement,' 'clinical quality,' 'perceived quality,' or another 'quality' term? Give exact text in quotation marks.	Text<br>99 = If Quality$2=0
Reference to Quality$4	Which of the six dimensions of quality[3] did the study address?<br>• *Safe*—delivering health care which minimises risks and harm to service users, including avoiding preventable injuries and reducing medical errors<br>• *Effective*—providing services based on scientific knowledge and evidence-based guidelines<br>• *Timely*—reducing delays in providing/receiving health care<br>• *Efficient*—delivering health care in a manner which maximises	1 = Safe<br>2 = Effective<br>3 = Timely<br>4 = Efficient<br>5 = Equitable<br>6 = People-centered<br>7 = Multiple (2-5) dimensions<br>8 = All dimensions<br>98 = Unclear/not specified

[3] Operational definitions for the characteristics of quality care come from WHO's vision for quality of care for pregnant women and newborns: https://www.ncbi.nlm.nih.gov/pmc/articles/PMC5029576/

Variable	Question and instruction	Response options
	resource use and avoids wastage<br>• *Equitable*—delivering health care which does not vary in quality because of personal characteristics such as gender, race, ethnicity, geographical location or socioeconomic status<br>• *People-centered*—providing care which takes into account the preferences and aspirations of individual service users and the cultures of their communities	
Reference to Quality$5	If Quality$4 = 7, specify the dimensions (in alphabetical order).	Text
Outcomes		
Critical Outcome$1A	Did the study report on the outcome 'maternal morbidity?'	0 = No<br>1 = Yes<br>98 = Unclear/not specified
Critical Outcome$1B	In the author's words, include the text describing the outcome 'maternal morbidity.'	Text<br>98 = Unclear/not specified<br>99 = If Outcom$1A=0
Critical Outcome$1C	Report all outcome data for 'maternal morbidity.'	Text<br>98 = Unclear/not specified<br>99 = If Outcom$1A=0
Critical Outcome$2A	Did the study report on the outcome 'maternal mortality?'	0 = No<br>1 = Yes<br>98 = Unclear/not specified
Critical Outcome$2B	In the author's words, include the text describing the outcome 'maternal mortality.'	Text<br>98 = Unclear/not specified<br>99 = If Outcom$2A=0
Critical Outcome$2C	Report all outcome data for 'maternal mortality.'	Text<br>98 = Unclear/not specified<br>99 = If Outcom$2A=0
Critical Outcome$3A	Did the study report on the outcome 'newborn morbidity?'	0 = No<br>1 = Yes<br>98 = Unclear/not specified
Critical Outcome$3B	In the author's words, include the text describing the outcome 'newborn morbidity.'	Text<br>98 = Unclear/not specified<br>99 = If Outcom$3A=0
Critical Outcome$3C	Report all outcome data for 'newborn morbidity.'	Text<br>98 = Unclear/not specified<br>99 = If Outcom$3A=0
Critical Outcome$4A	Did the study report on the outcome 'newborn mortality?'	0 = No<br>1 = Yes<br>98 = Unclear/not specified
Critical Outcome$4B	In the author's words, include the	Text

Variable	Question and instruction	Response options
	text describing the outcome ‘newborn mortality.’	98 = Unclear/not specified<br>99 = If Outcom$4A=0
Critical Outcome$4C	Report all outcome data for ‘newborn mortality.’	Text<br>98 = Unclear/not specified<br>99 = If Outcom$4A=0
Critical Outcome$5A	Did the study report on the outcome ‘child morbidity?’	0 = No<br>1 = Yes<br>98 = Unclear/not specified
Critical Outcome$5B	In the author’s words, include the text describing the outcome ‘child morbidity.’	Text<br>98 = Unclear/not specified<br>99 = If Outcom$5A=0
Critical Outcome$5C	Report all outcome data for ‘child morbidity.’	Text<br>98 = Unclear/not specified<br>99 = If Outcom$5A=0
Critical Outcome$6A	Did the study report on the outcome ‘child mortality?’	0 = No<br>1 = Yes<br>98 = Unclear/not specified
Critical Outcome$6B	In the author’s words, include the text describing the outcome ‘child mortality.’	Text<br>98 = Unclear/not specified<br>99 = If Outcom$6A=0
Critical Outcome$6C	Report all outcome data for ‘child mortality.’	Text<br>98 = Unclear/not specified<br>99 = If Outcom$6A=0
Critical Outcome$7A	Did the study report on the outcome ‘quality of care?’ This includes outcomes involving:<br>• Safety<br>• Efficacy<br>• Timeliness<br>• Efficiency<br>• Equitability<br>• People-centered care	0 = No<br>1 = Yes<br>98 = Unclear/not specified
Critical Outcome$7B	In the author’s words, include the text describing the outcome ‘quality of care.’	Text<br>98 = Unclear/not specified<br>99 = If Outcom$7A=0
Critical Outcome$7C	Report all outcome data for ‘quality of care.’	Text<br>98 = Unclear/not specified<br>99 = If Outcom$7A=0
Critical Outcome$8A	Did the study report on the outcome ‘experience of care, including respectful care?’	0 = No<br>1 = Yes<br>98 = Unclear/not specified
Critical Outcome$8B	In the author’s words, include the text describing the outcome ‘experience of care, including respectful care.’	Text<br>98 = Unclear/not specified<br>99 = If Outcom$8A=0
Critical Outcome$8C	Report all outcome data for ‘experience of care, including respectful care.’	Text<br>98 = Unclear/not specified<br>99 = If Outcom$2A=0
Critical Outcome$9A	Did the study report on the	0 = No

Variable	Question and instruction	Response options
	outcome 'service utilization and efficiency?'	1 = Yes<br>98 = Unclear/not specified
Critical Outcome$9B	In the author's words, include the text describing the outcome 'service utilization and efficiency.'	Text<br>98 = Unclear/not specified<br>99 = If Outcom$9A=0
Critical Outcome$9C	Report all outcome data for 'service utilization and efficiency.'	Text<br>98 = Unclear/not specified<br>99 = If Outcom$9A=0
Secondary Outcome$1A	Did the study report on the outcome 'infant and/or child growth?'	0 = No<br>1 = Yes<br>98 = Unclear/not specified
Secondary Outcome$1B	In the author's words, include the text describing the outcome 'infant and/or child growth.'	Text<br>98 = Unclear/not specified<br>99 = If Outcom$9A=0
Secondary Outcome$1C	Report all outcome data for 'infant and/or child growth.'	Text<br>98 = Unclear/not specified<br>99 = If Outcom$9A=0
Additional Outcomes	What other outcomes were described or measured? Give details. Include all outcomes relating to stillbirth, childbirth, health system outcomes, and women's views and satisfaction.	Text
Evaluation/study details		
Evaluated	Was the intervention evaluated?	0 = No<br>1 = Yes
Type of Evaluation$1	What type of evaluation was done?	1 = Impact (quantitative change in relevant outcome (use of services))<br>2 = Process (evaluation of intervention implementation)<br>3 = Impact and process<br>4 = Economic<br>5 = Multiple<br>6 = Other<br>98 = Unclear/not specified<br>99 = Not applicable
Type of Evaluation$2	Give details with quotations	Text<br>99 = Not applicable
Study Type$1	What was the design of the study?	1 = Randomized controlled trial<br>2 = Controlled clinical trial<br>3 = Cohort analytic (two groups pre + post)<br>4 = Case-control<br>5 = Cohort (one group pre + post (before and after))<br>6 = Interrupted time series<br>7 = Qualitative<br>8 = Mixed methods<br>9 = Regression analysis

Lattof SR, Maliqi B. *BMJ Open* 2023; 10:e033141. doi: 10.1136/bmjopen-2019-033141

Variable	Question and instruction	Response options
		10 = Other<br>98 = Unclear/not specified
Study Type$2	Give details with quotations	Text<br>99 = Not applicable
Control/Comparator$1	Control/comparator group included?	0 = No<br>1 = Yes<br>98 = Unclear/not specified
Control/Comparator$2	If Control/Comparator$1 = 1, give details	Text<br>99= Not applicable
Data Type	What type of data were presented in the evaluation findings/results?	1 = Quantitative<br>2 = Qualitative<br>3 = Both
Longitudinal Data	Were the data longitudinal?	0 = No<br>1 = Yes<br>98 = Unclear/not specified
Intervention Claims$1	What claims are made for the effects of the intervention on the outcome/s relevant to quality of maternal, newborn, and child health services?	1 = Positive<br>2 = Neutral<br>3 = Negative<br>4 = Mixed (give details)<br>5 = Other (give details)<br>98 = Unclear/not specified
Intervention Claims$2	Give details. Use author's words (with quotation marks).	Text<br>99 = Not applicable
Strategy Effectiveness	Copy any text about barriers/enablers to the intervention, factors influencing its delivery.	Text
Cost Data	Extract all data on the cost of the intervention and cost compared to outcomes.	Text
Cost Effectiveness Data	Extract all data on the cost effectiveness of the intervention.	Text
Mechanisms	Did the study discuss or propose any mechanisms for engaging the private sector in planning, delivering, and demonstrating accountability for quality maternal, newborn, and child health services? If so, give details and use the author's words (with quotation marks).	Text
Context	In what context was the study conducted? Give details (e.g. regulatory environment, collaborative service delivery models).	Text
Notes	Include any notes you think are relevant	Text

Variable	Question and instruction	Response options
Quality assessment (for quantitative studies): Reviewer 1		
Selection Bias$1	Are the individuals selected to participate in the study likely to be representative of the target population?	1 = Very likely<br>2 = Somewhat likely<br>3 = Not likely<br>98 = Unclear/not specified
Selection Bias$2	What percentage of selected individuals agreed to participate?	1 = 80-100% agreement<br>2 = 60-79% agreement<br>3 = Less than 60% agreement<br>98 = Unclear/not specified<br>99 = Not applicable
Selection Bias$3	Rate this section (see dictionary[4])	1 = Strong<br>2 = Moderate<br>3 = Weak
Study Design$1	Was the study described as randomized? (Check Study Type$1)<br><br>If NO, go to Confounders$1.	0 = No<br>1 = Yes
Study Design$2	If YES, was the method of randomization described? (see dictionary)	0 = No<br>1 = Yes
Study Design$3	If YES, was the method appropriate? (see dictionary)	0 = No<br>1 = Yes
Study Design$4	Rate this section (see dictionary)	1 = Strong<br>2 = Moderate<br>3 = Weak
Confounders$1	Were there important differences between groups prior to the intervention?<br><br>The following are examples of confounders:<br>1. Race<br>2. Sex<br>3. Marital status/family<br>4. Age<br>5. SES (income or class)<br>6. Education<br>7. Health status<br>Pre-intervention score on outcome measure	0 = No<br>1 = Yes<br>98 = Unclear/not specified
Confounders$2	If YES, indicate the percentage of relevant confounders that were controlled (either in the design (e.g. stratification, matching) or analysis)?	1 = 80-100% (most)<br>2 = 60-79% (some)<br>3 = Less than 60% (few or none)<br>98 = Unclear/not specified<br>99 = Confou$1=0 or 98
Confounders$3	Rate this section (see dictionary)	1 = Strong

[4] Follow EPHPP's 'Quality Assessment Tool for Quantitative Studies Dictionary' to score study quality for quantitative studies.

Variable	Question and instruction	Response options
		2 = Moderate<br>3 = Weak
Blinding$1	Was (were) the outcome assessor(s) aware of the information or exposure status of participants?	0 = No<br>1 = Yes<br>98 = Unclear/not specified
Blinding$2	Were the study participants aware of the research question?	0 = No<br>1 = Yes<br>98 = Unclear/not specified
Blinding$3	Rate this section (see dictionary)	1 = Strong<br>2 = Moderate<br>3 = Weak
Data Collection Methods$1	Were data collection tools shown to be valid?	0 = No<br>1 = Yes<br>98 = Unclear/not specified
Data Collection Methods$2	Were data collection tools shown to be reliable?	0 = No<br>1 = Yes<br>98 = Unclear/not specified
Data Collection Methods$3	Rate this section (see dictionary)	1 = Strong<br>2 = Moderate<br>3 = Weak
Withdrawals and Dropouts$1	Were withdrawals and dropouts reported in terms of numbers and/or reasons per group?	0 = No<br>1 = Yes<br>98 = Unclear/not specified<br>99 = Not applicable
Withdrawals and Dropouts$2	Indicate the percentage of participants completing the study. (If the percentage differs by groups, record the lowest.)	1 = 80-100%<br>2 = 60-79%<br>3 = Less than 60%<br>98 = Unclear/not specified<br>99 = Not applicable
Withdrawals and Dropouts$3	Rate this section (see dictionary)	1 = Strong<br>2 = Moderate<br>3 = Weak<br>99 = Not applicable
Intervention Integrity$1	What percentage of participants received the allocated intervention or exposure of interest?	1 = 80-100%<br>2 = 60-79%<br>3 = Less than 60%<br>98 = Unclear/not specified
Intervention Integrity$2	Was the consistency of the intervention measured?	0 = No<br>1 = Yes<br>98 = Unclear/not specified
Intervention Integrity$3	Is it likely that the subjects received an unintended intervention (contamination or co-intervention) that may influence the results?	0 = No<br>1 = Yes<br>98 = Unclear/not specified
Analyses$1	Indicate the unit of allocation.	1 = Community<br>2 = Organization/institution<br>3 = Practice/office

Variable	Question and instruction	Response options
		4 = Individual
Analyses$2	Indicate the unit of analysis.	1 = Community<br>2 = Organization/institution<br>3 = Practice/office<br>4 = Individual
Analyses$3	Are the statistical methods appropriate for the study design?	0 = No<br>1 = Yes<br>98 = Unclear/not specified
Analyses$4	Is the analysis performed by intervention allocation status (i.e. intention to treat) rather than the actual intervention received?	0 = No<br>1 = Yes<br>98 = Unclear/not specified
Global Rating$1	Based on the final section ratings for selection bias, study design, confounders, blinding, data collection methods, and withdrawals and dropouts, what is the global rating for this paper?	1 = Strong (no WEAK ratings)<br>2 = Moderate (one WEAK rating)<br>3 = Weak (two or more WEAK ratings)
Global Rating$2	With both reviewers discussing the ratings, is there a discrepancy between the two reviewers with respect to the following sections: for selection bias, study design, confounders, blinding, data collection methods, and withdrawals and dropouts	0 = No<br>1 = Yes
Global Rating$3	If YES, indicate the reason for the discrepancy.	1 = Oversight<br>2 = Differences in interpretation of criteria<br>3 = Differences in interpretation of study
Global Rating$4	Final decision of both reviewers	1 = Strong<br>2 = Moderate<br>3 = Weak
Quality assessment (for quantitative studies): Reviewer 2		
Selection Bias$4	Are the individuals selected to participate in the study likely to be representative of the target population?	1 = Very likely<br>2 = Somewhat likely<br>3 = Not likely<br>98 = Unclear/not specified
Selection Bias$5	What percentage of selected individuals agreed to participate?	1 = 80-100% agreement<br>2 = 60-79% agreement<br>3 = Less than 60% agreement<br>98 = Unclear/not specified<br>99 = Not applicable
Selection Bias$6	Rate this section (see dictionary[5])	1 = Strong<br>2 = Moderate

[5] Follow EPHPP's 'Quality Assessment Tool for Quantitative Studies Dictionary' to score study quality for quantitative studies.

Variable	Question and instruction	Response options
		3 = Weak
Study Design$5	Was the study described as randomized? (Check Study Type$1)<br><br>If NO, go to Confounders$4.	0 = No<br>1 = Yes
Study Design$6	If YES, was the method of randomization described? (see dictionary)	0 = No<br>1 = Yes
Study Design$7	If YES, was the method appropriate? (see dictionary)	0 = No<br>1 = Yes
Study Design$8	Rate this section (see dictionary)	1 = Strong<br>2 = Moderate<br>3 = Weak
Confounders$4	Were there important differences between groups prior to the intervention?<br><br>The following are examples of confounders:<br>8. Race<br>9. Sex<br>10. Marital status/family<br>11. Age<br>12. SES (income or class)<br>13. Education<br>14. Health status<br>Pre-intervention score on outcome measure	0 = No<br>1 = Yes<br>98 = Unclear/not specified
Confounders$5	If YES, indicate the percentage of relevant confounders that were controlled (either in the design (e.g. stratification, matching) or analysis)?	1 = 80-100% (most)<br>2 = 60-79% (some)<br>3 = Less than 60% (few or none)<br>98 = Unclear/not specified<br>99 = Confou$1=0 or 98
Confounders$6	Rate this section (see dictionary)	1 = Strong<br>2 = Moderate<br>3 = Weak
Blinding$4	Was (were) the outcome assessor(s) aware of the information or exposure status of participants?	0 = No<br>1 = Yes<br>98 = Unclear/not specified
Blinding$5	Were the study participants aware of the research question?	0 = No<br>1 = Yes<br>98 = Unclear/not specified
Blinding$6	Rate this section (see dictionary)	1 = Strong<br>2 = Moderate<br>3 = Weak
Data Collection Methods$4	Were data collection tools shown to be valid?	0 = No<br>1 = Yes<br>98 = Unclear/not specified

Variable	Question and instruction	Response options
Data Collection Methods$5	Were data collection tools shown to be reliable?	0 = No<br>1 = Yes<br>98 = Unclear/not specified
Data Collection Methods$6	Rate this section (see dictionary)	1 = Strong<br>2 = Moderate<br>3 = Weak
Withdrawals and Dropouts$4	Were withdrawals and dropouts reported in terms of numbers and/or reasons per group?	0 = No<br>1 = Yes<br>98 = Unclear/not specified<br>99 = Not applicable
Withdrawals and Dropouts$5	Indicate the percentage of participants completing the study. (If the percentage differs by groups, record the lowest.)	1 = 80-100%<br>2 = 60-79%<br>3 = Less than 60%<br>98 = Unclear/not specified<br>99 = Not applicable
Withdrawals and Dropouts$6	Rate this section (see dictionary)	1 = Strong<br>2 = Moderate<br>3 = Weak<br>99 = Not applicable
Intervention Integrity$4	What percentage of participants received the allocated intervention or exposure of interest?	1 = 80-100%<br>2 = 60-79%<br>3 = Less than 60%<br>98 = Unclear/not specified
Intervention Integrity$5	Was the consistency of the intervention measured?	0 = No<br>1 = Yes<br>98 = Unclear/not specified
Intervention Integrity$6	Is it likely that the subjects received an unintended intervention (contamination or co-intervention) that may influence the results?	0 = No<br>1 = Yes<br>98 = Unclear/not specified
Analyses$5	Indicate the unit of allocation.	1 = Community<br>2 = Organization/institution<br>3 = Practice/office<br>4 = Individual
Analyses$6	Indicate the unit of analysis.	1 = Community<br>2 = Organization/institution<br>3 = Practice/office<br>4 = Individual
Analyses$7	Are the statistical methods appropriate for the study design?	0 = No<br>1 = Yes<br>98 = Unclear/not specified
Analyses$8	Is the analysis performed by intervention allocation status (i.e. intention to treat) rather than the actual intervention received?	0 = No<br>1 = Yes<br>98 = Unclear/not specified
Global Rating$5	Based on the final section ratings for selection bias, study design, confounders, blinding, data	1 = Strong (no WEAK ratings)<br>2 = Moderate (one WEAK rating)<br>3 = Weak (two or more WEAK ratings)

Variable	Question and instruction	Response options
	collection methods, and withdrawals and dropouts, what is the global rating for this paper?	
Quality assessment (for qualitative studies): Reviewer 1		
Scope and Purpose$1	Does the study have a clear statement of, and rationale for, its research question/aims/purposes?<br><br>Specific prompts:<br>? Clarity of focus demonstrated<br>? Explicit purpose given, such as descriptive/explanatory intent, theory building, hypothesis testing<br>? Link between research and existing knowledge demonstrated	0 = No<br>1 = Yes
Scope and Purpose$2	Is the study thoroughly contextualized by existing literature?<br><br>Specific prompt:<br>? Evidence of systematic approach to literature review, location of literature to contextualize the findings, or both	0 = No<br>1 = Yes
Scope and Purpose$3	Rate this section.<br><br>When Purpose$1 and Purpose$2 = 1, then Purpose$3 = 1. When Purpose$1 or Purpose$2 = 0, then Purpose$3 = 2. When Purpose$1 and Purpose$2 = 0, then Purpose$3 = 3.	1 = Strong<br>2 = Moderate<br>3 = Weak
Design$1	Is the method/design apparent and consistent with the research intent?<br><br>Specific prompts:<br>? Rationale given for use of qualitative design<br>? Discussion of epistemological/ontological grounding<br>? Rationale explored for specific qualitative method (e.g. ethnography, grounded theory, phenomenology)<br>? Discussion of why particular method chosen is most appropriate/sensitive/relevant for research question/aims	0 = No<br>1 = Yes

Variable	Question and instruction	Response options
	? Setting appropriate	
Design$2	Is the data collection strategy apparent and appropriate?<br><br>Specific prompts:<br>? Were data collection methods appropriate for type of data required and for specific qualitative method?<br>? Were they likely to capture the complexity/diversity of experience and illuminate context in sufficient detail?<br>??Was triangulation of data sources used if appropriate?	0 = No<br>1 = Yes
Design$3	Rate this section<br><br>When Design$1 and Design$2 = 1, then Design$3 = 1. When Design$1 or Design$2 = 0, then Design$3 = 2. When Design$1 and Design$2 = 0, then Design$3 = 3.	1 = Strong<br>2 = Moderate<br>3 = Weak
Sampling Strategy$1	Are the sampling and sampling method appropriate?<br><br>Specific prompts:<br>? Selection criteria detailed, and description of how sampling was undertaken<br>? Justification for sampling strategy given<br>? Thickness of description likely to be achieved from sampling<br>? Any disparity between planned and actual sample explained	0 = No<br>1 = Yes
Sampling Strategy$2	Rate this section<br><br>When Strategy$1 = 1, then Strategy$2 = 1. When Strategy$1 = 0, then Strategy$2 = 3. At the reviewer's discretion, Strategy$2 may be rated as 2, if the study met most but not all of the prompts.	1 = Strong<br>2 = Moderate<br>3 = Weak
Analysis$1	Is the analytical approach appropriate?<br><br>Specific prompts:<br>? Approach made explicit (e.g. Thematic distillation, constant comparative method, grounded	0 = No<br>1 = Yes

Variable	Question and instruction	Response options
	theory)<br>⍰ Was it appropriate for the qualitative method chosen?<br>⍰ Was data managed by software package or by hand and why?<br>⍰ Discussion of how coding systems/conceptual frameworks evolved<br>⍰ How was context of data retained during analysis<br>⍰ Evidence that the subjective meanings of participants were portrayed<br>⍰ Evidence of more than one researcher involved in stages if appropriate to epistemological/theoretical stance<br>⍰ Did research participants have any involvement in analysis (e.g. member checking)<br>⍰ Evidence provided that data reached saturation or discussion/rationale if it did not<br>⍰ Evidence that deviant data was sought, or discussion/rationale if it was not	
Analysis$2	Rate this section<br><br>When Analysis$1 = 1, then Analysis$2 = 1. When Analysis$1 = 0, then Analysis$2 = 3. At the reviewer's discretion, Analysis$2 may be rated as 2, if the study met most but not all of the prompts.	1 = Strong<br>2 = Moderate<br>3 = Weak
Interpretation$1	Is the context described and taken account of in the interpretation?<br><br>Specific prompts:<br>⍰ Description of social/physical and interpersonal contexts of data collection<br>⍰ Evidence that researcher spent time 'dwelling with the data', interrogating it for competing/alternative explanations of phenomena	0 = No<br>1 = Yes
Interpretation$2	Is a clear audit trail given?<br><br>Specific prompt:<br>⍰ Sufficient discussion of research	0 = No<br>1 = Yes

Variable	Question and instruction	Response options
	processes such that others can follow the 'decision trail'	
Interpretation$3	Are data used to support interpretation?<br><br>Specific prompts:<br>? Extensive use of field notes entries/verbatim interview quotes in discussion of findings<br>? Clear exposition of how interpretation led to conclusions	0 = No<br>1 = Yes
Interpretation$4	Rate this section<br><br>When Interpretation$1, Interpretation$2, and Interpretation$3 = 1, then Interpretation$4 = 1. When one response to Interpretation$1—Interpretation$3 = 0, then Interpretation$4 = 2. When two or more responses to Interpretation$1—Interpretation$3 = 0, then Interpretation$4 = 3.	1 = Strong<br>2 = Moderate<br>3 = Weak
Reflexivity$1	Is researcher reflexivity demonstrated?<br><br>Specific prompts:<br>? Discussion of relationship between researcher and participants during fieldwork<br>? Demonstration of researcher's influence on stages of research process<br>? Evidence of self-awareness/insight<br>? Documentation of effects of the research on researcher<br>? Evidence of how problems/complications met were dealt with	0 = No<br>1 = Yes
Reflexivity$2	Rate this section<br><br>When Reflexivity$1 = 1, then Reflexivity$2 = 1. When Reflexivity$1 = 0, then Reflexivity$2 = 3. At the reviewer's discretion, Reflexivity$2 may be rated as 2, if the study met most but not all of the prompts.	1 = Strong<br>2 = Moderate<br>3 = Weak
Ethical Dimensions$1	Is there demonstration of	0 = No

Variable	Question and instruction	Response options
	sensitivity to ethical concerns?<br><br>Specific prompts:<br>? Ethical committee approval granted<br>? Clear commitment to integrity, honesty, transparency, equality and mutual respect in relationships with participants<br>? Evidence of fair dealing with all research participants<br>? Recording of dilemmas met and how resolved in relation to ethical issues<br>? Documentation of how autonomy, consent, confidentiality, anonymity were managed	1 = Yes
Ethical Dimensions$2	Rate this section<br><br>When Dimensions$1 = 1, then Dimensions$2 = 1. When Dimensions$1 = 0, then Dimensions$2 = 3. At the reviewer's discretion, Dimensions$2 may be rated as 2, if the study met most but not all of the prompts.	1 = Strong<br>2 = Moderate<br>3 = Weak
Relevance and Transferability$1	Are relevance and transferability evident?<br><br>Specific prompts:<br>? Sufficient evidence for typicality specificity to be assessed<br>? Analysis interwoven with existing theories and other relevant explanatory literature drawn from similar settings and studies<br>? Discussion of how explanatory propositions/emergent theory may fit other contexts<br>? Limitations/weaknesses of study clearly outlined<br>? Clearly resonates with other knowledge and experience<br>? Results/conclusions obviously supported by evidence<br>? Interpretation plausible and 'makes sense'<br>? Provides new insights and increases understanding	0 = No<br>1 = Yes

Variable	Question and instruction	Response options
	? Significance for current policy and practice outlined<br>? Assessment of value/empowerment for participants<br>? Outlines further directions for investigation<br>? Comment on whether aims/purposes of research were achieved	
Relevance and Transferability$2	Rate this section<br><br>Transferability$1 = 1, then Transferability$2 = 1. When Transferability$1 = 0, then Transferability$2 = 3. At the reviewer's discretion, Transferability$2 may be rated as 2, if the study met most but not all of the prompts.	1 = Strong<br>2 = Moderate<br>3 = Weak
Global Rating$1	Based on the final section ratings for scope and purpose, design, sampling strategy, analysis, interpretation, reflexivity, ethical dimensions, and relevance and transferability, what is the global rating for this paper?	1 = Strong (no WEAK ratings)<br>2 = Moderate (one WEAK rating)<br>3 = Weak (two or more WEAK ratings)
Global Rating$2	With both reviewers discussing the ratings, is there a discrepancy between the two reviewers with respect to the following sections: scope and purpose, design, sampling strategy, analysis, interpretation, reflexivity, ethical dimensions, and relevance and transferability	0 = No<br>1 = Yes
Global Rating$3	If YES, indicate the reason for the discrepancy.	1 = Oversight<br>2 = Differences in interpretation of criteria<br>3 = Differences in interpretation of study
Global Rating$4	Final decision of both reviewers	1 = Strong<br>2 = Moderate<br>3 = Weak
Quality assessment (for qualitative studies): Reviewer 2		
Scope and Purpose$4	Does the study have a clear statement of, and rationale for, its research question/aims/purposes?	0 = No<br>1 = Yes

Variable	Question and instruction	Response options
	Specific prompts:<br>? Clarity of focus demonstrated<br>? Explicit purpose given, such as descriptive/explanatory intent, theory building, hypothesis testing<br>? Link between research and existing knowledge demonstrated	
Scope and Purpose$5	Is the study thoroughly contextualized by existing literature?<br><br>Specific prompt:<br>? Evidence of systematic approach to literature review, location of literature to contextualize the findings, or both	0 = No<br>1 = Yes
Scope and Purpose$6	Rate this section.<br><br>When Purpose$4 and Purpose$5 = 1, then Purpose$6 = 1. When Purpose$4 or Purpose$5 = 0, then Purpose$6 = 2. When Purpose$4 and Purpose$5 = 0, then Purpose$6 = 3.	1 = Strong<br>2 = Moderate<br>3 = Weak
Design$4	Is the method/design apparent and consistent with the research intent?<br><br>Specific prompts:<br>? Rationale given for use of qualitative design<br>? Discussion of epistemological/ontological grounding<br>? Rationale explored for specific qualitative method (e.g. ethnography, grounded theory, phenomenology)<br>? Discussion of why particular method chosen is most appropriate/sensitive/relevant for research question/aims<br>? Setting appropriate	0 = No<br>1 = Yes
Design$5	Is the data collection strategy apparent and appropriate?<br><br>Specific prompts:<br>? Were data collection methods appropriate for type of data required and for specific qualitative	0 = No<br>1 = Yes

Variable	Question and instruction	Response options
	method?<br>? Were they likely to capture the complexity/diversity of experience and illuminate context in sufficient detail?<br>??Was triangulation of data sources used if appropriate?	
Design$6	Rate this section<br><br>When Design$4 and Design$5 = 1, then Design$6 = 1. When Design$4 or Design$5 = 0, then Design$6 = 2. When Design$4 and Design$5 = 0, then Design$6 = 3.	1 = Strong<br>2 = Moderate<br>3 = Weak
Sampling Strategy$3	Are the sampling and sampling method appropriate?<br><br>Specific prompts:<br>? Selection criteria detailed, and description of how sampling was undertaken<br>? Justification for sampling strategy given<br>? Thickness of description likely to be achieved from sampling<br>? Any disparity between planned and actual sample explained	0 = No<br>1 = Yes
Sampling Strategy$4	Rate this section<br><br>When Strategy$3 = 1, then Strategy$4 = 1. When Strategy$3 = 0, then Strategy$4 = 3. At the reviewer's discretion, Strategy$4 may be rated as 2, if the study met most but not all of the prompts.	1 = Strong<br>2 = Moderate<br>3 = Weak
Analysis$3	Is the analytical approach appropriate?<br><br>Specific prompts:<br>? Approach made explicit (e.g. Thematic distillation, constant comparative method, grounded theory)<br>? Was it appropriate for the qualitative method chosen?<br>? Was data managed by software package or by hand and why?<br>? Discussion of how coding systems/conceptual frameworks evolved	0 = No<br>1 = Yes

Variable	Question and instruction	Response options
	? How was context of data retained during analysis<br>? Evidence that the subjective meanings of participants were portrayed<br>? Evidence of more than one researcher involved in stages if appropriate to epistemological/theoretical stance<br>? Did research participants have any involvement in analysis (e.g. member checking)<br>? Evidence provided that data reached saturation or discussion/rationale if it did not<br>? Evidence that deviant data was sought, or discussion/rationale if it was not	
Analysis$4	Rate this section<br><br>When Analysis$3 = 1, then Analysis$4 = 1. When Analysis$3 = 0, then Analysis$4 = 3. At the reviewer's discretion, Analysis$4 may be rated as 2, if the study met most but not all of the prompts.	1 = Strong<br>2 = Moderate<br>3 = Weak
Interpretation$5	Is the context described and taken account of in the interpretation?<br><br>Specific prompts:<br>? Description of social/physical and interpersonal contexts of data collection<br>? Evidence that researcher spent time 'dwelling with the data', interrogating it for competing/alternative explanations of phenomena	0 = No<br>1 = Yes
Interpretation$6	Is a clear audit trail given?<br><br>Specific prompt:<br>? Sufficient discussion of research processes such that others can follow the 'decision trail'	0 = No<br>1 = Yes
Interpretation$7	Are data used to support interpretation?<br><br>Specific prompts:<br>? Extensive use of field notes entries/verbatim interview quotes	0 = No<br>1 = Yes

Variable	Question and instruction	Response options
	in discussion of findings<br>? Clear exposition of how interpretation led to conclusions	
Interpretation$8	Rate this section<br><br>When Interpretation$5, Interpretation$6, and Interpretation$7 = 1, then Interpretation$8 = 1. When one response to Interpretation$5—Interpretation$7 = 0, then Interpretation$8 = 2. When two or more responses to Interpretation$5—Interpretation$7 = 0, then Interpretation$8 = 3.	1 = Strong<br>2 = Moderate<br>3 = Weak
Reflexivity$3	Is researcher reflexivity demonstrated?<br><br>Specific prompts:<br>? Discussion of relationship between researcher and participants during fieldwork<br>? Demonstration of researcher's influence on stages of research process<br>? Evidence of self-awareness/insight<br>? Documentation of effects of the research on researcher<br>? Evidence of how problems/complications met were dealt with	0 = No<br>1 = Yes
Reflexivity$4	Rate this section<br><br>When Reflexivity$3 = 1, then Reflexivity$4 = 1. When Reflexivity$3 = 0, then Reflexivity$4 = 3. At the reviewer's discretion, Reflexivity$4 may be rated as 2, if the study met most but not all of the prompts.	1 = Strong<br>2 = Moderate<br>3 = Weak
Ethical Dimensions$3	Is there demonstration of sensitivity to ethical concerns?<br><br>Specific prompts:<br>? Ethical committee approval granted<br>? Clear commitment to integrity, honesty, transparency, equality and mutual respect in relationships	0 = No<br>1 = Yes

Variable	Question and instruction	Response options
	with participants<br>? Evidence of fair dealing with all research participants<br>? Recording of dilemmas met and how resolved in relation to ethical issues<br>? Documentation of how autonomy, consent, confidentiality, anonymity were managed	
Ethical Dimensions$4	Rate this section<br><br>When Dimensions$3 = 1, then Dimensions$4 = 1. When Dimensions$3 = 0, then Dimensions$4 = 3. At the reviewer's discretion, Dimensions$4 may be rated as 2, if the study met most but not all of the prompts.	1 = Strong<br>2 = Moderate<br>3 = Weak
Relevance and Transferability$3	Are relevance and transferability evident?<br><br>Specific prompts:<br>? Sufficient evidence for typicality specificity to be assessed<br>? Analysis interwoven with existing theories and other relevant explanatory literature drawn from similar settings and studies<br>? Discussion of how explanatory propositions/emergent theory may fit other contexts<br>? Limitations/weaknesses of study clearly outlined<br>? Clearly resonates with other knowledge and experience<br>? Results/conclusions obviously supported by evidence<br>? Interpretation plausible and 'makes sense'<br>? Provides new insights and increases understanding<br>? Significance for current policy and practice outlined<br>? Assessment of value/empowerment for participants<br>? Outlines further directions for investigation<br>? Comment on whether	0 = No<br>1 = Yes

Variable	Question and instruction	Response options
	aims/purposes of research were achieved	
Relevance and Transferability$4	Rate this section<br><br>Transferability$3 = 1, then Transferability$4 = 1. When Transferability$3 = 0, then Transferability$4 = 3. At the reviewer's discretion, Transferability$4 may be rated as 2, if the study met most but not all of the prompts.	1 = Strong<br>2 = Moderate<br>3 = Weak
Global Rating$5	Based on the final section ratings for scope and purpose, design, sampling strategy, analysis, interpretation, reflexivity, ethical dimensions, and relevance and transferability, what is the global rating for this paper?	1 = Strong (no WEAK ratings)<br>2 = Moderate (one WEAK rating)<br>3 = Weak (two or more WEAK ratings)
