# Supplementary material for: Private sector delivery of quality care for maternal, newborn and child health in low-income and middle-income countries: a mixed-methods systematic review protocol
Source: BMJ Open. 2020 Feb 17;10(2):e033141. doi: 10.1136/bmjopen-2019-033141 (PMC7045217; doi:10.1136/bmjopen-2019-033141)
Supplement: Author's manuscript [file bmjopen-2019-033141.draft_revisions.pdf]

BMJ Open is committed to open peer review. As part of this commitment we make the peer review history of every article we publish publicly available.

When an article is published we post the peer reviewers' comments and the authors' responses online. We also post the versions of the paper that were used during peer review. These are the versions that the peer review comments apply to.

The versions of the paper that follow are the versions that were submitted during the peer review process. They are not the versions of record or the final published versions. They should not be cited or distributed as the published version of this manuscript.

BMJ Open is an open access journal and the full, final, typeset and author-corrected version of record of the manuscript is available on our site with no access controls, subscription charges or pay-per-view fees (http://bmjopen.bmj.com).

If you have any questions on BMJ Open's open peer review process please email info.bmjopen@bmj.com

BMJ Open

Private sector delivery of quality care for maternal, newborn, and child health: A mixed-methods systematic review protocol

Journal:	*BMJ Open*
Manuscript ID	bmjopen-2019-033141
Article Type:	Protocol
Date Submitted by the Author:	22-Jul-2019
Complete List of Authors:	Lattof, Samantha; World Health Organization, Department of Maternal, Newborn, Child and Adolescent Health<br>Maliqi, Blerta; World Health Organization, Department of Maternal, Newborn, Child and Adolescent Health
Keywords:	private sector, maternal health, newborn health, child health, Quality in health care < HEALTH SERVICES ADMINISTRATION & MANAGEMENT, systematic review

SCHOLARONE™
Manuscripts

Title

Private sector delivery of quality care for maternal, newborn, and child health: A mixed-methods systematic review protocol

Authors

Samantha R. Lattof, PhD; Department of Maternal, Newborn, Child and Adolescent Health; World Health Organization; Avenue Appia 20, 1202 Geneva, Switzerland; lattofs@who.int

Blerta Maliqi, MD; Department of Maternal, Newborn, Child and Adolescent Health; World Health Organization; Avenue Appia 20, 1202 Geneva, Switzerland; maliqib@who.int

Keywords

private sector, maternal health, newborn health, child health, quality of care, systematic review, protocols & guidelines

Word count

2,971 words

For peer review only

Abstract

Introduction

To accelerate progress to reach the Sustainable Development Goals for ending preventable maternal, newborn, and child deaths, it is critical that both the public and private health service delivery systems invest in increasing coverage of interventions to sustainably deliver quality care for mothers, newborns, and children at scale. Although various approaches have been successful in high-income countries, little is known about how to effectively engage and sustain private sector involvement in delivering quality care in low- and middle-income countries. Our systematic review will examine private sector implementation of quality care for maternal, newborn, and child health (MNCH) and the impact of this care. This protocol details our intended methodological and analytical approaches, based on the Preferred Reporting Items for Systematic reviews and Meta-Analyses (PRISMA) reporting guideline for protocols.

Methods and analysis

Following the PRISMA approach, this systematic review will include quantitative, qualitative, and mixed-methods studies addressing the provision of quality MNCH care by private sector providers. For inclusion, studies in low- and middle-income countries must examine at least one of the following outcomes: maternal morbidity or mortality; newborn morbidity or mortality; child morbidity or mortality; quality of care; experience of care; and service utilization and efficiency. Depending on the results, analyses could include meta-analysis, descriptive quantitative statistics, narrative synthesis, and thematic synthesis. Quality will be assessed using tools for qualitative and quantitative studies.

Ethics and dissemination

Formal ethical approval is not required for this research, as the secondary data are not identifiable. Findings from this review will be used to develop models for effective collaboration of the private and public sectors in implementing quality of care for MNCH. In addition to publishing our findings in a peer-reviewed journal, the findings will be shared through the Quality of Care Network, relevant mailing lists, webinars, and social media.

For peer review only

Strengths and limitations of this study

- This systematic review protocol is the first to examine private sector delivery of quality maternal, newborn, and child health care in low- and middle-income countries.
- By using the PRISMA checklist, we increase the potential usefulness, clarity, and transparency with which we will report the results form this study.
- To minimize potential bias from the publication of positive results and to increase the validity of this systematic review, we will make a concerted effort to supplement our electronic database searches with grey literature and programmatic reports.
- We acknowledge a risk of bias in locating studies, as it is possible evidence on the provision of quality health care by the private sector may be disseminated internally beyond public reach.
- For transparency and quality, the protocol includes a codebook and data extraction template.

For peer review only

Introduction

The private sector plays a key role in delivering sexual and reproductive health services. It provides a substantial proportion of family planning services among women aged 15-49 years in Asia (45%), Latin America and the Caribbean (44%), and sub-Saharan Africa (28%).[1] In certain countries, like Democratic Republic of Congo and Nigeria, the private sector's family planning market share exceeds 60%.[2] One in five births in low- and middle-income countries occurred with care delivered by the private sector.[3] Despite the private sector's expanding role in many countries, the quality of services varies. To accelerate progress to reach the Sustainable Development Goals for ending preventable maternal, newborn, and child deaths, it is critical that both the public and private health service delivery systems invest in increasing coverage of interventions to sustainably deliver quality care at scale.

The private sector addresses an increasing volume of maternal, newborn, and child health (MNCH) care needs amongst countries in the Network for Improving Quality of Care for Maternal, Newborn and Child Health (the Network). The Network, a consortium of eleven countries and their technical partners, aims to halve maternal and newborn deaths and stillbirths in health facilities in five years' time.[4] Whilst the Network's efforts to achieve this ambitious goal have largely focused on strengthening the public health sector, members of the Network recognize that private providers (i.e., non-government providers, for-profit businesses) are an important source of health care and have a role to play in improving quality care. However, little is known about how to effectively engage and sustain private sector involvement in delivering quality care in low and middle-income countries. This gap must be addressed, if the Network is to achieve its aims of reducing maternal and newborn deaths and stillbirths.

The engagement and contribution of the private sector in implementing quality care standards, developing and identifying best practices for delivering quality MNCH care, and strengthening health systems for delivering quality are areas of great potential that require immediate attention. There is a need to understand what can be done to create, to nurture, and to encourage a vibrant private sector that is fully engaged in improving and sustaining quality of care for mothers, newborns, and children. We aim to begin filling these knowledge gaps by conducting a systematic review that addresses the following research questions:

Primary research questions

1. How does the provision of quality health care by the private sector affect morbidity and mortality among mothers, newborns, and children?
2. How does provision of quality health care by the private sector affect utilization of services by mothers, newborns, and children?
3. How effective and efficient is the private sector at delivering quality of care?
4. Among mothers, newborns, and children utilizing health care provided by the private sector, what are their experiences of care?

Secondary research questions

For peer review only

5. What mechanisms[a] exist for engaging the private sector in delivering quality maternal, newborn, and child health services?
6. What types of regulatory and collaborative service delivery models[b] exist between the public and private sectors to deliver quality of care?

This protocol details our intended methodological and analytical approaches for the systematic review, based on the Preferred Reporting Items for Systematic reviews and Meta-Analyses (PRISMA) reporting guideline for protocols.[5]

Methods and analysis

This systematic review will utilize the PRISMA checklist for reporting systematic reviews and meta-analyses so as to ensure clarity and transparency.[6]

Inclusion/exclusion criteria

This review focuses on studies examining the private sector's involvement in delivering quality health care to pregnant women, mothers, newborns, and children in low- and middle-income countries. Journal articles and grey literature (e.g., reports, book chapters), both peer-reviewed and non-peer-reviewed, will be eligible for inclusion. The PICOTS (population, interventions, control, outcomes, timeframe, setting) criteria, detailed in Table 1, provide an overview of our inclusion criteria. Quantitative, qualitative, and mixed-methods studies in low- and middle-income countries will be considered, provided that they report on one of the following outcomes:

- Maternal morbidity or mortality
- Newborn morbidity or mortality
- Child morbidity or mortality
- Quality of care
- Experience of care, including respectful care
- Service utilization and efficiency

In recognition of the rapid increase in public-private collaborations for health during the late 1990s,[7] items must be published from 1 January 1995 to 30 June 2019. Items will be eligible if published in English, French, German, or Italian. For inclusion, quality must be a specific focus amongst the interventions being implemented, such as quality assessment, quality improvement or the delivery of quality care.

Table 1. PICOTS criteria used in the systematic review

PICOTS	
Populations	Pregnant women, mothers, newborns, and children

[a] These mechanisms may address demonstrating accountability for quality MNCH services, the development and implementation of MNCH standards and quality improvement approaches, the development of quality of care implementation packages for MNCH, and the development of policies and plans for quality MNCH.

[b] These models may include the private sector's involvement in national MNCH quality of care structures and coordinating mechanisms.

For peer review only

Interventions	Delivery of quality maternal, newborn, and/or child health services by the private sector
Control	None
Outcomes	Quantitative, qualitative, or mixed-methods data on:<br>- Maternal morbidity or mortality<br>- Newborn morbidity or mortality<br>- Child morbidity or mortality<br>- Quality of care<br>- Experience of care, including respectful care<br>- Service utilization and efficiency
Timeframe	1 January 1995 to 30 June 2019
Setting	Low- and middle-income countries

As we are focused on service delivery, we are limiting the private sector to providers (e.g., private health facilities, private health providers, civil society organizations delivering care, charities delivering care). Thus, private suppliers (e.g., pharmaceutical companies) and private payers (e.g., private health insurance companies) will be excluded. Unless commentaries and editorials introduce new evidence, we will exclude them in favour of the original study or studies being discussed. Study protocols will also be excluded.

Search strategy and terms

We will search the following eight electronic databases that were assessed for likely coverage, availability, and relevance of literature on the private sector:

- Cumulative Index to Nursing and Allied Health (CINAHL)
- EconLit
- Excerpta Medica Database (EMBASE)
- International Bibliography of the Social Sciences (IBSS)
- Popline
- PubMed
- ScienceDirect
- Web of Science

Whilst we considered the LILACS database that includes literature on Latin America and the Caribbean, our test search returned no results. A test search in Open Grey returned one extraneous result, so we excluded that database as well. Based on discussions with experts and an initial assessment of the electronic databases, we suspect that a significant body of literature on the private sector is published as grey literature or programmatic reports. Thus, we will make a concerted effort to supplement our electronic database searches with expert-recommended articles and reports that we will obtain by sending a standardized request for literature to a list of experts and working groups identified in collaboration with colleagues at WHO.

Given our interest in locating grey literature, we will also conduct a targeted search of publications on two websites: Health Care Provider Performance Review (HCPPR) and the Maternal healthcare markets Evaluation Team (MET) at the London School of Hygiene & Tropical Medicine. HCPPR is a database of over 700 studies from a systematic review on the

For peer review only

effectiveness of strategies to improve healthcare provider performance in low- and middle-income countries.[8] HCPPR's search strategy included extensive searching of document inventories for unpublished studies. MET conducts multidisciplinary research on the role of public and private health sectors in delivering maternal healthcare. Its website includes both peer-reviewed literature and grey literature. We acknowledge risk of bias in locating studies. Our efforts to locate evidence on the provision of quality health care by the private sector may be limited, specifically when such evidence is only disseminated internally where it is beyond public reach.

Our searches will be conducted using combinations of search terms detailed in Table 2. These terms aim to capture all studies examining the private sector; quality of care; and maternal, newborn, and child health (MNCH). The terms were developed and tested for sensitivity, and they will be adapted to the basic search particulars (e.g., wildcards (*), capacity for complex searches) of each electronic database.

Table 2. Search terms and their combinations

1. Private sector	2. Quality of care	3. MNCH
private sector	quality	matern*
for-profit		pregnan*
for profit		mother*
public-private		newborn*
private enterprise*		infant*
NGO		child*
non-government*		pediatric*
		paediatric*

Screening process

Searches and application of the inclusion/exclusion criteria will be conducted according to the PRISMA flow approach. Citation abstracts for all items uncovered by our searches will be exported into EndNote for screening. After first removing duplicates, remaining items will be screened for inclusion on the basis of title and abstract (TIAB). If inclusion/exclusion cannot be determined based on the TIAB, then the item will be pushed forward for full-text screening. SL will conduct all TIAB and full-text screening, seeking guidance from BM on items considered borderline or problematic.

Data extraction

The data extraction form (Annex B) will be piloted with five randomly selected studies. Any necessary changes will be discussed amongst the authors prior to full data extraction. Based on the guidance in our codebook (Annex A), SL will extract data for all studies into the data extraction template in Excel. Data will be extracted on the following categories:

- Background information (e.g., author, date, setting, study objective)
- Intervention background information (e.g., implementing agency, geographic level, study population)

For peer review only

- Intervention details (e.g., intervention recipients, nature of intervention)
- Critical outcomes (both quantitative and qualitative):
  - Maternal morbidity or mortality
  - Newborn morbidity or mortality
  - Child morbidity or mortality
  - Quality of care
  - Experience of care, including respectful care
  - Service utilization and efficiency
- Evaluation/study details (e.g., study type, data type, intervention claims, strategy effectiveness, cost data)

Study quality and risks of bias will be assessed in duplicate using quality assessment tools for quantitative and qualitative studies.[9-11]

Data synthesis

Once the data have been extracted, we will analyse the quantitative and qualitative findings separately. Depending on the quantitative studies and data available in the final inventory, a meta-analysis may be appropriate to examine impacts of the private sector's provision of quality MNCH care (e.g., outcomes on morbidity, mortality, service utilization and efficiency). The meta-analysis will be limited to studies rated as strong or moderate quality, and meta-biases of included studies will be assessed using ROBIS.[12] If high heterogeneity exists between study designs, populations, and definitions, then we will instead present the quantitative findings using a narrative synthesis to report the data thematically with tables of descriptive quantitative statistics and study outcomes.

For the qualitative data, we will enter the verbatim findings into NVivo for a three-step thematic synthesis.[13] Each line of text will initially be coded based on its content and meaning. All codes will be included in a codebook with new codes added when appropriate. The codes will then be related together to develop categories and linkages between the data. After reviewing the data for analytical themes, we will make inferences about the provision of quality maternal, newborn, and child health care by the private sector. We suspect that this type of synthesis will be particularly well suited to data on patients' experiences of care by the private sector and existing mechanisms for engaging the private sector in delivering quality MNCH services.

Patient and public involvement

The design of this systematic review protocol did not involve patients, though we hope the final inventory of studies included in this review will report on patients' experiences of care. The need for this systematic review was initiated by discussions with public sector health officials in Network countries.

Final search strategy by database

The full electronic search strategies for all databases, including limits and filters used, appear below.

For peer review only

CINAHL

Search strategy: We will search all sets of search terms (Table 2).

Search options:
- Search mode: Boolean/phrase
- Limit results:
  - Published date: January 1995 to June 2019
  - Language: English, French, German, Italian

Search terms: ("private sector" OR for-profit OR "for profit" OR public-private OR "private enterprise*" OR NGO OR non-government*) AND (quality) AND (matern* OR pregnan* OR mother* OR newborn* OR infant* OR child* OR pediatric* OR paediatric*)

EconLit

Search strategy: We will search all sets of search terms (Table 2).

Search options:
- Search mode: Boolean/phrase
- Limit results:
  - Published date: January 1995 to June 2019

Search terms: ("private sector" OR for-profit OR "for profit" OR public-private OR "private enterprise*" OR NGO OR non-government*) AND (quality) AND (matern* OR pregnan* OR mother* OR newborn* OR infant* OR child* OR pediatric* OR paediatric*)

EMBASE

Search strategy: We will search all search terms (Table 2) using the multi-field search in the abstract field.

Search options:
- Limit results:
  - Publication year: 1995 – current
  - Language: English, French, German, Italian

Search terms: (private sector OR for-profit OR for profit OR public-private OR private enterprise* OR NGO OR non-government*) AND (quality) AND (matern* OR pregnan* OR mother* OR newborn* OR infant* OR child* OR pediatric* OR paediatric*)

HCPPR

Search strategy: For this systematic review database on the effectiveness of strategies to improve health care provider performance in low- and middle-income countries, we will obtain a list of all included grey literature on the private sector directly from the project investigator.

For peer review only

IBSS

Search strategy: We will search all sets of search terms (Table 2) using the advanced search feature. To minimize extraneous results returned during test searches, searches will be conducted "anywhere except full text." Thus, the searches will use title and abstract, in line with our initial plans for TIAB screening.

Search options:

- Limit results:
  - Publication date: 1 January 1995 – 30 June 2019
  - Language: English, French, German, Italian

Search terms: noft(("private sector" OR for-profit OR "for profit" OR public-private OR "private enterprise*" OR NGO OR non-government*) AND (quality) AND (matern* OR pregnan* OR mother* OR newborn* OR infant* OR child* OR pediatric* OR paediatric*))

MET

Search strategy: On the MET publications page, we will export all documents that have not been published in peer-reviewed journals (e.g., reports, policy briefs). Peer-reviewed publications appear in journals that have been indexed in other electronic databases included in this systematic review.

Popline

Search strategy: We will search all sets of search terms (Table 2). Since the database does not permit users to include wildcards inside quotation marks, we will split "private enterprise*" into two terms: "private enterprise" and "private enterprises."

Search options:

- Filters:
  - Year: Published since 1995
  - Language: English, French, German, Italian

Search terms: (("private sector" OR for-profit OR "for profit" OR public-private OR "private enterprise" OR "private enterprises" OR NGO OR non-government*) AND (quality) AND (matern* OR pregnan* OR mother* OR newborn* OR infant* OR child* OR pediatric* OR paediatric*))

PubMed

Search strategy: We will search all sets of search terms (Table 2) using the advanced search builder.

Search options:

- Limit results:

For peer review only

- Publication dates: From 1 January 1995 to 30 June 2019
- Language: English, French, German, Italian

Search terms: ("private sector" OR for-profit OR "for profit" OR public-private OR "private enterprise*" OR NGO OR non-government*) AND (quality) AND (matern* OR pregnan* OR mother* OR newborn* OR infant* OR child* OR pediatric* OR paediatric*)

ScienceDirect

Search strategy: Since this database does not support wildcards (*) or more than eight Boolean connectors per field, we will search a modified set of search terms (Table 2) using the advanced search feature and limiting keywords to those most widely used. We will split the terms between articles and title, abstract, or keywords, as detailed below.

Search options:

- Limit results:
  - Year(s): 1995-2019

Search terms:

- Find articles with these terms: (maternal OR maternity OR newborn OR child OR children OR childhood OR childbirth OR pregnancy OR pregnant)
- Title, abstract or keywords: (quality) AND ("private sector" OR for-profit OR "for profit" OR public-private OR "private enterprise" OR "private enterprises" OR NGO OR non-governmental)

Web of Science

Search strategy: We will search all sets of search terms (Table 2) using the advanced search feature and topic (TS) field tag.

Search options:

- Limit results:
  - Year(s): 1995-2019
  - Language: English, French, German, Italian

Search terms: ("private sector" OR for-profit OR "for profit" OR public-private OR "private enterprise*" OR NGO OR non-government*) AND (quality) AND (matern* OR pregnan* OR mother* OR newborn* OR infant* OR child* OR pediatric* OR paediatric*)

Ethics and dissemination

Formal ethical approval is not required for this research, as the publically available secondary data are not identifiable.

This systematic review will provide evidence on different models that can be drawn upon by countries in planning and implementing their national plans and processes for quality of

For peer review only

care. Findings from this review will be used to develop models for effective collaboration of the private and public sectors in implementing quality of care for MNCH. In addition to publishing our findings in a peer-reviewed journal, the findings will be shared through the Quality of Care Network, relevant mailing lists, webinars, and social media.

Should we need to amend this protocol following its publication, we will ensure that future publications arising from this protocol provide the date of each amendment, describe the change(s), and report the rationale for the change(s).

Acknowledgements

We wish to thank David Clarke and Wilson Were (WHO) for their contributions to the wider discussions around this research and Alex Rowe (Centers for Disease Control and Prevention) for sharing information on the HCPPR database, including a list of private sector grey literature. This protocol has been submitted to PROSPERO for registration.

Contributors

BM and SL conceived the idea for the review. SL designed and wrote the first draft of the protocol. Both authors contributed to subsequent revisions and approved the protocol prior to its submission. SL is the guarantor.

Funding

This work was supported by MSD for Mothers. This funder had no role in the design and development of the study protocol or the decision to publish.

Competing interests

We declare no competing interests.

Data sharing statement

The data extraction workbook and codebook are available as supplementary annexes.

References

1. Ugaz JI, Chatterji M, Gribble JN, et al. Regional trends in the use of short-acting and long-acting contraception accessed through the private and public sectors. *International Journal of Gynecology & Obstetrics* 2015;130(S3):E3-E7. doi: 10.1016/j.ijgo.2015.03.021

For peer review only

2. Dennis ML, Benova L, Owolabi OO, et al. Meeting need vs. sharing the market: a systematic review of methods to measure the use of private sector family planning and childbirth services in sub-Saharan Africa. *BMC health services research* 2018;18(1):699. doi: 10.1186/s12913-018-3514-y [published Online First: 2018/09/12]
3. Benova L, Macleod D, Footman K, et al. Role of the private sector in childbirth care: cross-sectional survey evidence from 57 low- and middle-income countries using Demographic and Health Surveys. *Tropical Medicine & International Health* 2015;20(12):1657-73. doi: 10.1111/tmi.12598
4. Adeniran A, Likaka A, Knutsson A, et al. Leadership, action, learning and accountability to deliver quality care for women, newborns and children. *Bull World Health Organ* 2018;96(3):222-24. doi: 10.2471/BLT.17.197939 [published Online First: 02/05]
5. Moher D, Shamseer L, Clarke M, et al. Preferred reporting items for systematic review and meta-analysis protocols (PRISMA-P) 2015 statement. *Systematic Reviews* 2015;4(1):1. doi: 10.1186/2046-4053-4-1
6. Liberati A, Altman DG, Tetzlaff J, et al. The PRISMA statement for reporting systematic reviews and meta-analyses of studies that evaluate healthcare interventions: explanation and elaboration. *BMJ* 2009;339:b2700. doi: 10.1136/bmj.b2700
7. Buse K, Walt G. Global public-private partnerships: part I - a new development in health? *WHO Bulletin* 2000;78(4):549-61.
8. Rowe SY, Peters DH, Holloway KA, et al. A systematic review of the effectiveness of strategies to improve health care provider performance in low- and middle-income countries: Methods and descriptive results. *PLOS ONE* 2019;14(5):e0217617. doi: 10.1371/journal.pone.0217617
9. EPHPP. Quality assessment tool for quantitative studies Hamilton: Effective Public Health Practice Project 2010 [Available from: https://merst.ca/ephpp/.
10. Walsh D, Downe S. Appraising the quality of qualitative research. *Midwifery* 2006;22(2):108-19. doi: 10.1016/j.midw.2005.05.004 [published Online First: 2005/10/26]
11. Solnes Miltenburg A, Roggeveen Y, Elteren Mv, et al. A protocol for a systematic review of birth preparedness and complication readiness programs. *Systematic Reviews* 2013;2(1):11. doi: 10.1186/2046-4053-2-11
12. Whiting P, Savovic J, Higgins JP, et al. ROBIS: A new tool to assess risk of bias in systematic reviews was developed. *Journal of clinical epidemiology* 2016;69:225-34. doi: 10.1016/j.jclinepi.2015.06.005 [published Online First: 2015/06/21]
13. Thomas J, Harden A. Methods for the thematic synthesis of qualitative research in systematic reviews. *BMC Medical Research Methodology* 2008;8(1):45. doi: 10.1186/1471-2288-8-45

Reviewer's initials	Number assigned to each article/study (e.g., 001, 002)	First author's surname	Full name of the study or article	Year of publication
**Reviewer**	**Study ID**	**Author**	**Study Name**	**Publication Date**

peer review only

Background information				
State the exclusion criteria for the study, or state "none."<br><br>*If study meets any of the exclusion criteria, end data extraction.*	Does this study meet all inclusion criteria? Check PICOTS, language, year, and inclusion of quality. If all criteria are met, state "all."	Type of publication	If PubTyp = 5, give details.	Detail any other publications linked to the study (e.g. papers describing methods, additional analyses)
**Exclusion Criteria**	**Inclusion Criteria**	**Publication Type$1**	**Publication Type$2**	**Other Publications**

For peer review only

Language of publication	List all countries in which the study was conducted	Country(ies) income group	World region(s)	State the primary or main objective(s)/aim(s) of the study	Give (Month/Year) or (Year)
**Language of Publication**	**Country**	**Country Income Group**	**World Region**	**Study Objective**	**Intervention Start Date**

peer review only

Intervention back					
Give (Month/Year) or (Year)	Intervention duration in months	Which agency or agencies implemented the intervention?	Details	At which geographical level did the study occur?	Give specific detail for previous response (e.g. Bongo district (17 villages, 6,158 inhabitants))
**Intervention End Date**	**Intervention Duration**	**Implementing Agency$1**	**Implementing Agency$2**	**Geographic Level$1**	**Geographic Level$2**

For peer review only

ground information

What was the main identifying characteristic of the population targeted?	Give specific detail for previous response (e.g., women in the third trimester). Use author's words (with quotation marks)	Provide the number enrolled in the intervention population	Provide the number enrolled in the control population	Provide the mean age of the intervention population	Provide the mean age of the control population
**Study Population$1**	**Study Population$2**	**Study Population$3**	**Study Population$4**	**Study Population$5**	**Study Population$6**

peer review only

Provide the mean age of the combined population (intervention and control)	Copy all text describing the intervention; ensure comprehensive extraction of intervention characteristics	Did the study examine a single intervention or multiple interventions?	Was the intervention focused on supply-side or demand-side factors?	Were women during pregnancy, childbirth and/or postpartum intervention recipients? (Unless it is explicit, it is a no).	Was a subgroup of women during pregnancy, childbirth, postpartum (e.g., women with HIV) the intervention recipient? (Unless it is explicit, it is a no).
**Study Population$7**	**Intervention Description**	**Intervention Quantity**	**Intervention Supply--Demand**	**Intervention Recipient$1**	**Intervention Recipient$2**

peer review only

Intervent					
Were women defined more generally (e.g., all women, all women of reproductive age) intervention recipients? (Unless it is explicit, it is a no).	Were newborns intervention recipients? (Unless it is explicit, it is a no).	Were children intervention recipients? (Unless it is explicit, it is a no).	Were health care providers intervention recipients? (Unless it is explicit, it is a no).	Were any others intervention recipients? (Unless it is explicit, it is a no).	Give specific detail for previous response (Intervention recipient code: “author’s description”)
**Intervention Recipient$3**	**Intervention Recipient$4**	**Intervention Recipient$5**	**Intervention Recipient$6**	**Intervention Recipient$7**	**Intervention Recipient$8**

peer review only

tion details					
Was on-site support for quality improvement introduced/changed as part of the intervention? (Unless it is explicit, it is a no).	Were data systems introduced/changed as part of the intervention? (Unless it is explicit, it is a no).	Were learning systems introduced/changed as part of the intervention? (Unless it is explicit, it is a no).	Was a program management selected/changed as part of the intervention? (Unless it is explicit, it is a no).	Was advocacy introduced/ provided as part of the intervention? (Unless it is explicit, it is a no).	Were other systems introduced or changed as part of the intervention?
**Nature of Intervention$1**	**Nature of Intervention$2**	**Nature of Intervention$3**	**Nature of Intervention$4**	**Nature of Intervention$5**	**Nature of Intervention$6**

peer review only

Give specific details for previous responses (e.g., coaching, training, electronic data systems, community involvement) (Nature of intervention code: “author’s description”)	Did the text explicitly use one of the following quality terms: ‘quality of care,’ ‘quality care,’ ‘quality assessment,’ ‘quality improvement’ or another ‘quality’ term?	Did the text explicitly define ‘quality of care,’ ‘quality care,’ ‘quality assessment,’ ‘quality improvement’ or another ‘quality’ term?	If Quality$2 = 1, how did the text define/refer to ‘quality of care,’ ‘quality care,’ ‘quality assessment,’ ‘quality improvement’ or another ‘quality’ term?’ Give exact text in quotation marks.	Did the study report on the outcome ‘maternal morbidity?’
**Nature of Intervention$7**	**Reference to Quality$1**	**Reference to Quality$2**	**Reference to Quality$3**	**Critical Outcome$1A**

peer review only

In the author's words, include the text describing the outcome 'maternal morbidity.'	Report all outcome data for 'maternal morbidity.'	Did the study report on the outcome 'maternal mortality?'	In the author's words, include the text describing the outcome 'maternal mortality.'	Report all outcome data for 'maternal mortality.'	Did the study report on the outcome 'newborn morbidity?'	In the author's words, include the text describing the outcome 'newborn morbidity.'
**Critical Outcome$1B**	**Critical Outcome$1C**	**Critical Outcome$2A**	**Critical Outcome$2B**	**Critical Outcome$2C**	**Critical Outcome$3A**	**Critical Outcome$3B**

peer review only

Outcomes						
Report all outcome data for 'newborn morbidity.'	Did the study report on the outcome 'newborn mortality?'	In the author's words, include the text describing the outcome 'newborn mortality.'	Report all outcome data for 'newborn mortality.'	Did the study report on the outcome 'child morbidity?'	In the author's words, include the text describing the outcome 'child morbidity.'	Report all outcome data for 'child morbidity.'
**Critical Outcome$3C**	**Critical Outcome$4A**	**Critical Outcome$4B**	**Critical Outcome$4C**	**Critical Outcome$5A**	**Critical Outcome$5B**	**Critical Outcome$5C**

For peer review only

Did the study report on the outcome 'child mortality?'	In the author's words, include the text describing the outcome 'child mortality.'	Report all outcome data for 'child mortality.'	Did the study report on the outcome 'quality of care?'	In the author's words, include the text describing the outcome 'quality of care.'	Report all outcome data for 'quality of care.'
**Critical Outcome$6A**	**Critical Outcome$6B**	**Critical Outcome$6C**	**Critical Outcome$7A**	**Critical Outcome$7B**	**Critical Outcome$7C**

peer review only

Did the study report on the outcome 'experience of care, including respectful care?'	In the author's words, include the text describing the outcome 'experience of care, including respectful care.'	Report all outcome data for 'experience of care, including respectful care.'	Did the study report on the outcome 'service utilization and efficiency?'	In the author's words, include the text describing the outcome 'service utilization and efficiency.'	Report all outcome data for 'service utilization and efficiency.'
**Critical Outcome$8A**	**Critical Outcome$8B**	**Critical Outcome$8C**	**Critical Outcome$9A**	**Critical Outcome$9B**	**Critical Outcome$9C**

For peer review only

What other outcomes were described or measured? Give details. Include all outcomes relating to stillbirth, childbirth, health system outcomes, and women's views and satisfaction.	Was the intervention evaluated?	What type of evaluation was done?	Give details with quotations	What was the design of the study?
**Additional Outcomes**	**Evaluated**	**Type of Evaluation$1**	**Type of Evaluation$2**	**Study Type$1**

peer review only

Evaluation/study details				
Give details with quotations	Control or comparator group included?	If Control/ Comparator$1 = 1, give details	What type of data were presented in the evaluation findings/results?	Were the data longitudinal?
**Study Type$2**	**Control/ Comparator$1**	**Control/ Comparator$2**	**Data Type**	**Longitudinal Data**

peer review only

s				
What claims are made for the effects of the intervention on the outcome/s relevant to quality of maternal, newborn, and child health care services?	Give details. Use author's words (with quotation marks)	Copy any text about barriers/enablers to the intervention, factors influencing its delivery.	Extract all data on the cost of the intervention and cost compared to outcomes.	Did the study discuss or propose any mechanisms for engaging the private sector in planning, delivering, and demonstrating accountability for quality maternal, newborn, and child health services? If so, give details and use the author's words (with quotation marks).
**Intervention Claim$1**	**Intervention Claim$2**	**Strategy Effectiveness**	**Cost Data**	**Mechanisms**

peer review only

Context	Notes	Selection Bias$1	Selection Bias$2	Selection Bias$3	Study Design$1
In what context was the study conducted? Give details (e.g. regulatory environment, collaborative service delivery models).	Include any notes you think are relevant	Are the individuals selected to participate in the study likely to be representative of the target population?	What percentage of selected individuals agreed to participate?	Rate this section (see dictionary)	Was the study described as randomized? (Check Study Type$1) If NO, go to Confounders$1.

peer review only

If YES, was the method of randomization described? (see dictionary)	If YES, was the method appropriate? (see dictionary)	Rate this section (see dictionary)	Were there important differences between groups prior to the intervention?	If YES, indicate the percentage of relevant confounders that were controlled (either in the design (e.g. stratification, matching) or analysis)?	Rate this section (see dictionary)
**Study Design$2**	**Study Design$3**	**Study Design$4**	**Confounders$1**	**Confounders$2**	**Confounders$3**

peer review only

Quality assessment (for quantitative studies): Re						
Was (were) the outcome assessor(s) aware of the information or exposure status of participants?	Were the study participants aware of the research question?	Rate this section (see dictionary)	Were data collection tools shown to be valid?	Were data collection tools shown to be reliable?	Rate this section (see dictionary)	Were withdrawals and dropouts reported in terms of numbers and/or reasons per group?
**Blinding$1**	**Blinding$2**	**Blinding$3**	**Data Collection Methods$1**	**Data Collection Methods$2**	**Data Collection Methods$3**	**Withdrawals and Dropouts$1**

peer review only

eviewer 1					
Indicate the percentage of participants completing the study. (If the percentage differs by groups, record the lowest.)	Rate this section (see dictionary)	What percentage of participants received the allocated intervention or exposure of interest?	Was the consistency of the intervention measured?	Is it likely that the subjects received an unintended intervention (contamination or co-intervention) that may influence the results?	Indicate the unit of allocation.
**Withdrawals and Dropouts$2**	**Withdrawals and Dropouts$3**	**Intervention Integrity$1**	**Intervention Integrity$2**	**Intervention Integrity$3**	**Analyses$1**

peer review only

Indicate the unit of analysis.	Are the statistical methods appropriate for the study design?	Is the analysis performed by intervention allocation status (i.e. intention to treat) rather than the actual intervention received?	Based on the final section ratings for selection bias, study design, confounders, blinding, data collection methods, and withdrawals and dropouts, what is the global rating for this paper?	With both reviewers discussing the ratings, is there a discrepancy between the two reviewers with respect to the following sections: for selection bias, study design, confounders, blinding, data collection methods, and withdrawals and dropouts
**Analyses$2**	**Analyses$3**	**Analyses$4**	**Global Rating$1**	**Global Rating$2**

peer review only

Global Rating$3	Global Rating$4	Selection Bias$4	Selection Bias$5	Selection Bias$6	Study Design$5
If YES, indicate the reason for the discrepancy.	Final decision of both reviewers	Are the individuals selected to participate in the study likely to be representative of the target population?	What percentage of selected individuals agreed to participate?	Rate this section (see dictionary)	Was the study described as randomized? (Check Study Type$1) If NO, go to Confounders$ 4.

For peer review only

If YES, was the method of randomization described? (see dictionary)	If YES, was the method appropriate? (see dictionary)	Rate this section (see dictionary)	Were there important differences between groups prior to the intervention?	If YES, indicate the percentage of relevant confounders that were controlled (either in the design (e.g. stratification, matching) or analysis)?	Rate this section (see dictionary)
**Study Design$6**	**Study Design$7**	**Study Design$8**	**Confounders$4**	**Confounders$5**	**Confounders$6**

peer review only

Quality assessment (for quantitative studies): Reviewer 2						
Was (were) the outcome assessor(s) aware of the information or exposure status of participants?	Were the study participants aware of the research question?	Rate this section (see dictionary)	Were data collection tools shown to be valid?	Were data collection tools shown to be reliable?	Rate this section (see dictionary)	Were withdrawals and dropouts reported in terms of numbers and/or reasons per group?
**Blinding$4**	**Blinding$5**	**Blinding$6**	**Data Collection Methods$4**	**Data Collection Methods$5**	**Data Collection Methods$6**	**Withdrawals and Dropouts$4**

peer review only

Indicate the percentage of participants completing the study. (If the percentage differs by groups, record the lowest.)	Rate this section (see dictionary)	What percentage of participants received the allocated intervention or exposure of interest?	Was the consistency of the intervention measured?	Is it likely that the subjects received an unintended intervention (contamination or co-intervention) that may influence the results?	Indicate the unit of allocation.
**Withdrawals and Dropouts$5**	**Withdrawals and Dropouts$6**	**Intervention Integrity$4**	**Intervention Integrity$5**	**Intervention Integrity$6**	**Analyses$5**

peer review only

Indicate the unit of analysis.	Are the statistical methods appropriate for the study design?	Is the analysis performed by intervention allocation status (i.e. intention to treat) rather than the actual intervention received?	Based on the final section ratings for selection bias, study design, confounders, blinding, data collection methods, and withdrawals and dropouts, what is the global rating for this paper?	Does the study have a clear statement of, and rationale for, its research question/aims/ purposes?	Is the study thoroughly contextualized by existing literature?
**Analyses$6**	**Analyses$7**	**Analyses$8**	**Global Rating$5**	**Scope and Purpose$1**	**Scope and Purpose$2**

peer review only

Rate this section	Is the method/design apparent and consistent with the research intent?	Is the data collection strategy apparent and appropriate?	Rate this section	Are the sampling and sampling method appropriate?	Rate this section
**Scope and Purpose$3**	**Design$1**	**Design$2**	**Design$3**	**Sampling Strategy$1**	**Sampling Strategy$2**

peer review only

Quality assessment (for qualit				
Is the analytical approach appropriate?	Rate this section	Is the context described and taken account of in the interpretation?	Is a clear audit trail given?	Are data used to support interpretation?
**Analysis$1**	**Analysis$2**	**Interpretation$1**	**Interpretation$2**	**Interpretation$3**

peer review only

tative studies): Reviewer 1

Rate this section	Is researcher reflexivity demonstrated?	Rate this section	Is there demonstration of sensitivity to ethical concerns?	Rate this section	Are relevance and transferability evident?
**Interpretation$4**	**Reflexivity$1**	**Reflexivity$2**	**Ethical Dimensions$1**	**Ethical Dimensions$2**	**Relevance and Transferability$1**

peer review only

Rate this section	Based on the final section ratings for scope and purpose, design, sampling strategy, analysis, interpretation, reflexivity, ethical dimensions, and relevance and transferability, what is the global rating for this paper?	With both reviewers discussing the ratings, is there a discrepancy between the two reviewers with respect to the following sections: scope and purpose, design, sampling strategy, analysis, interpretation, reflexivity, ethical dimensions, and relevance and transferability	If YES, indicate the reason for the discrepancy.	Final decision of both reviewers
**Relevance and Transferability$2**	**Global Rating$1**	**Global Rating$2**	**Global Rating$3**	**Global Rating$4**

peer review only

Does the study have a clear statement of, and rationale for, its research question/aims/purposes?	Is the study thoroughly contextualized by existing literature?	Rate this section	Is the method/design apparent and consistent with the research intent?	Is the data collection strategy apparent and appropriate?	Rate this section
**Scope and Purpose$4**	**Scope and Purpose$5**	**Scope and Purpose$6**	**Design$4**	**Design$5**	**Design$6**

peer review only

Quality assessment (for qualitative stu					
Are the sampling and sampling method appropriate?	Rate this section	Is the analytical approach appropriate?	Rate this section	Is the context described and taken account of in the interpretation?	Is a clear audit trail given?
**Sampling Strategy$3**	**Sampling Strategy$4**	**Analysis$3**	**Analysis$4**	**Interpretation$5**	**Interpretation$6**

peer review only

dies): Reviewer 2

Are data used to support interpretation?	Rate this section	Is researcher reflexivity demonstrated?	Rate this section	Is there demonstration of sensitivity to ethical concerns?	Rate this section
**Interpretation$7**	**Interpretation$8**	**Reflexivity$3**	**Reflexivity$4**	**Ethical Dimensions$3**	**Ethical Dimensions$4**

peer review only

Are relevance and transferability evident?	Rate this section	Based on the final section ratings for scope and purpose, design, sampling strategy, analysis, interpretation, reflexivity, ethical dimensions, and relevance and transferability, what is the global rating for this paper?
**Relevance and Transferability$3**	**Relevance and Transferability$4**	**Global Rating$5**

For peer review only

Annex B: Codebook

Variable	Question and instruction	Response options
Background information		
Reviewer	State your initials	SL<br>BM
Study ID	Number assigned to each article/study (e.g., 001, 002)	#
Author	First author's surname	Text
Study Name	Full name of the study or article	Text
Publication Date	Year of publication	#
Exclusion Criteria	State the exclusion criteria for the study, or state "none."<br><br>*If study meets any of the exclusion criteria, end data extraction.*	Text
Inclusion Criteria	Does this study meet all inclusion criteria? Check PICOTS, language, year, and inclusion of quality. If all criteria are met, state "all."<br><br>*If all of the inclusion criteria are not met, return to "exclusion criteria."*	All
Publication Type$1	Type of publication	1 = Peer-reviewed journal article<br>2 = Non-peer-reviewed journal article<br>3 = Report<br>4 = Book or book chapter<br>5 = Other
Publication Type$2	If PubTyp = 5, give details.	Text<br>99 = Not applicable
Other Publications	Detail any other publications linked to the study (e.g. papers describing methods, additional analyses)	Text
Language of Publication		1 = English<br>2 = French<br>3 = German<br>4 = Italian<br>5 = Other
Country	List all countries in which the study was conducted	Text
Country Income Group[1]	Country income group	1 = Low<br>2 = Lower-middle<br>3 = Upper-middle<br>4 = Multiple
World Region[2]	World region(s)	1 = Africa

[1] World Bank Atlas method groupings of countries by income: https://datahelpdesk.worldbank.org/knowledgebase/articles/906519
[2] WHO region groupings: Africa, Asia, Europe, Latin America and the Caribbean, Northern America, Oceania: https://unstats.un.org/unsd/methodology/m49/

For peer review only - http://bmjopen.bmj.com/site/about/guidelines.xhtml

Variable	Question and instruction	Response options
		2 = Asia<br>3 = Europe<br>4 = Latin America and the Caribbean<br>5 = Northern America<br>6 = Oceania<br>7 = Multiple
Study Objective	State the primary or main objective(s) or aim(s) of the study	Text
Intervention background information		
Intervention Start Date	Give (Month/Year) or (Year)<br><br>[Give start date of implementation rather than preliminary work]	(MM/YYYY) or (YYYY)<br>98 = Unclear/not specified
Intervention End Date	Give (Month/Year) or (Year)	(MM/YYYY) or (YYYY)<br>Ongoing= Ongoing at time of publication<br>98= Unclear/not specified
Intervention Duration	Duration of the intervention in months. If not straightforward, give details.	Text<br>98 = Unclear/not specified
Implementing Agency$1	Which type of agency or agencies implemented the intervention?	1 = Domestic government (in the case of a public-private partnership)<br>2 = Domestic non-profit organization (e.g., charity, civil society organization)<br>3 = Domestic for-profit organization<br>4 = International non-governmental organization (NGO)<br>5 = International for-profit organization<br>6 = Other international institution<br>7 = Multiple answers from list<br>8 = Other<br>98 = Unclear/not specified
Implementing Agency$2	If Agency$1 = 1-8, give details. (e.g., international NGO "Health Unlimited"…)	Text<br>99 = Not applicable
Geographic Level$1	At which geographical level did the study occur?	1 = National<br>2 = Sub-national (e.g., region, state, county, district, whole city)<br>3 = Local (e.g., village, neighborhood)<br>4 = Health facility<br>5 = Other<br>98 = Unclear/not specified
Geographic Level$2	Give specific detail for previous response (e.g. Bongo district (17 villages, 6,158 inhabitants)). Provide name if only one location unit.	Text<br>99 = if GeoLvl$1 = 98

For peer review only - http://bmjopen.bmj.com/site/about/guidelines.xhtml

Variable	Question and instruction	Response options
Study Population$1	What was the main identifying characteristic of the population the study targeted?	1 = Pregnant women<br>2 = Women during childbirth<br>3 = Mothers postpartum<br>4 = Newborns<br>5 = Children<br>6 = Health care providers<br>7 = Multiple answers from list<br>8 = Other, specify<br>98 = Unclear/not specified<br>99 = Not applicable
Study Population$2	Give specific detail for previous response (e.g., women in the third trimester). Use author's words (with quotation marks).	Text
Study Population$3	Provide the number enrolled in the intervention population.	#<br>98 = Unclear/not specified
Study Population$4	Provide the number enrolled in the control population, if applicable.	#<br>98 = Unclear/not specified<br>99 = No control population
Study Population$5	Provide the mean age of the intervention population.	#<br>98 = Unclear/not specified
Study Population$6	Provide the mean age of the control population, if applicable.	#<br>98 = Unclear/not specified<br>99 = No control population
Study Population$7	Provide the mean age of the combined population (intervention and control), if applicable.	#<br>98 = Unclear/not specified<br>99 = No control population
Intervention details		
Intervention Description	Copy all text describing the intervention; ensure comprehensive extraction of intervention characteristics.	Text
Intervention Quantity	Did the study examine a single intervention or multiple interventions?	1 = Single intervention<br>2 = Multiple interventions<br>98 = Unclear/not specified
Intervention Supply – Demand	Was the intervention focused on supply-side or demand-side factors?	1 = Supply side (e.g., staff training, change in types of staff, service environment, provider communication skills)<br>2 = Demand-side (e.g., education, peer encouragement for service use)<br>3 = Both<br>98 = Unclear/not specified
Intervention Recipient$1	Were women during pregnancy, childbirth and/or postpartum intervention recipients?<br>(Unless it is explicit, it is a no).	1 = Indirect<br>2 = Direct<br>3 = Not a recipient
Intervention Recipient$2	Was a subgroup of women during pregnancy, childbirth, postpartum	1 = Indirect<br>2 = Direct

For peer review only - http://bmjopen.bmj.com/site/about/guidelines.xhtml

Variable	Question and instruction	Response options
	(e.g., women who experience birth complications, women with HIV) the intervention recipient?<br>(Unless it is explicit, it is a no).	3 = Not a recipient
Intervention Recipient$3	Were women defined more generally (e.g., all women, all women of reproductive age) intervention recipients?<br>(Unless it is explicit, it is a no).	1 = Indirect<br>2 = Direct<br>3 = Not a recipient
Intervention Recipient$4	Were newborns intervention recipients?<br>(Unless it is explicit, it is a no).	1 = Indirect<br>2 = Direct<br>3 = Not a recipient
Intervention Recipient$5	Were children intervention recipients?<br>(Unless it is explicit, it is a no).	1 = Indirect<br>2 = Direct<br>3 = Not a recipient
Intervention Recipient$6	Were health care providers intervention recipients?<br>(Unless it is explicit, it is a no).	1 = Indirect<br>2 = Direct<br>3 = Not a recipient
Intervention Recipient$7	Were any others intervention recipients?<br>(Unless it is explicit, it is a no).	1 = Indirect<br>2 = Direct<br>3 = Not a recipient
Intervention Recipient$8	Give specific detail for previous response (Intervention recipient code: "author's description")	Text<br>99 = If Recipient$1-Recipient$7 = 3
Nature of Intervention$1	Was on-site support for quality improvement introduced/changed as part of the intervention?<br>(Unless it is explicit, it is a no).	0 = No<br>1 = Yes
Nature of Intervention$2	Were data systems introduced/changed as part of the intervention?<br>(Unless it is explicit, it is a no).	0 = No<br>1 = Yes
Nature of Intervention$3	Were learning systems introduced/changed as part of the intervention?<br>(Unless it is explicit, it is a no).	0 = No<br>1 = Yes
Nature of Intervention$4	Was a program management selected/changed as part of the intervention?<br>(Unless it is explicit, it is a no).	0 = No<br>1 = Yes
Nature of Intervention$5	Was advocacy introduced/ provided as part of the intervention?<br>(Unless it is explicit, it is a no).	0 = No<br>1 = Yes
Nature of Intervention$6	Were other systems introduced or changed as part of the intervention?	0 = No<br>1 = Yes
Nature of Intervention$7	Give specific details for previous responses (e.g., coaching, training,	Text

For peer review only

Variable	Question and instruction	Response options
	electronic data systems, community involvement)<br>(Nature of intervention code: "author's description")	
Reference to Quality$1	Did the text explicitly use one of the following quality terms: 'quality of care,' 'quality care,' 'quality assessment,' 'quality improvement' or another 'quality' term?	0 = No<br>1 = Yes, quality of care or quality care<br>2 = Yes, quality assessment<br>3 = Yes, quality improvement<br>4 = Yes, multiple quality terms<br>5 = Yes, another quality term [specify in Quality$3]
Reference to Quality$2	Did the text explicitly define 'quality of care,' 'quality care,' 'quality assessment,' 'quality improvement' or another 'quality' term?	0 = No<br>1 = Yes
Reference to Quality$3	If Quality$2 = 1, how did the text define/refer to 'quality of care,' 'quality care,' 'quality assessment,' 'quality improvement' or another 'quality' term? Give exact text in quotation marks.	Text<br>99 = If Quality$2=0
Outcomes		
Critical Outcome$1A	Did the study report on the outcome 'maternal morbidity?'	0 = No<br>1 = Yes<br>98 = Unclear/not specified
Critical Outcome$1B	In the author's words, include the text describing the outcome 'maternal morbidity.'	Text<br>98 = Unclear/not specified<br>99 = If Outcom$1A=0
Critical Outcome$1C	Report all outcome data for 'maternal morbidity.'	Text<br>98 = Unclear/not specified<br>99 = If Outcom$1A=0
Critical Outcome$2A	Did the study report on the outcome 'maternal mortality?'	0 = No<br>1 = Yes<br>98 = Unclear/not specified
Critical Outcome$2B	In the author's words, include the text describing the outcome 'maternal mortality.'	Text<br>98 = Unclear/not specified<br>99 = If Outcom$2A=0
Critical Outcome$2C	Report all outcome data for 'maternal mortality.'	Text<br>98 = Unclear/not specified<br>99 = If Outcom$2A=0
Critical Outcome$3A	Did the study report on the outcome 'newborn morbidity?'	0 = No<br>1 = Yes<br>98 = Unclear/not specified
Critical Outcome$3B	In the author's words, include the text describing the outcome 'newborn morbidity.'	Text<br>98 = Unclear/not specified<br>99 = If Outcom$3A=0
Critical Outcome$3C	Report all outcome data for 'newborn morbidity.'	Text<br>98 = Unclear/not specified

For peer review only - http://bmjopen.bmj.com/site/about/guidelines.xhtml

Variable	Question and instruction	Response options
		99 = If Outcom$3A=0
Critical Outcome$4A	Did the study report on the outcome 'newborn mortality?'	0 = No<br>1 = Yes<br>98 = Unclear/not specified
Critical Outcome$4B	In the author's words, include the text describing the outcome 'newborn mortality.'	Text<br>98 = Unclear/not specified<br>99 = If Outcom$4A=0
Critical Outcome$4C	Report all outcome data for 'newborn mortality.'	Text<br>98 = Unclear/not specified<br>99 = If Outcom$4A=0
Critical Outcome$5A	Did the study report on the outcome 'child morbidity?'	0 = No<br>1 = Yes<br>98 = Unclear/not specified
Critical Outcome$5B	In the author's words, include the text describing the outcome 'child morbidity.'	Text<br>98 = Unclear/not specified<br>99 = If Outcom$5A=0
Critical Outcome$5C	Report all outcome data for 'child morbidity.'	Text<br>98 = Unclear/not specified<br>99 = If Outcom$5A=0
Critical Outcome$6A	Did the study report on the outcome 'child mortality?'	0 = No<br>1 = Yes<br>98 = Unclear/not specified
Critical Outcome$6B	In the author's words, include the text describing the outcome 'child mortality.'	Text<br>98 = Unclear/not specified<br>99 = If Outcom$6A=0
Critical Outcome$6C	Report all outcome data for 'child mortality.'	Text<br>98 = Unclear/not specified<br>99 = If Outcom$6A=0
Critical Outcome$7A	Did the study report on the outcome 'quality of care?'	0 = No<br>1 = Yes<br>98 = Unclear/not specified
Critical Outcome$7B	In the author's words, include the text describing the outcome 'quality of care.'	Text<br>98 = Unclear/not specified<br>99 = If Outcom$7A=0
Critical Outcome$7C	Report all outcome data for 'quality of care.'	Text<br>98 = Unclear/not specified<br>99 = If Outcom$7A=0
Critical Outcome$8A	Did the study report on the outcome 'experience of care, including respectful care?'	0 = No<br>1 = Yes<br>98 = Unclear/not specified
Critical Outcome$8B	In the author's words, include the text describing the outcome 'experience of care, including respectful care.'	Text<br>98 = Unclear/not specified<br>99 = If Outcom$8A=0
Critical Outcome$8C	Report all outcome data for 'experience of care, including respectful care.'	Text<br>98 = Unclear/not specified<br>99 = If Outcom$2A=0
Critical Outcome$9A	Did the study report on the outcome 'service utilization and	0 = No<br>1 = Yes

For peer review only - http://bmjopen.bmj.com/site/about/guidelines.xhtml

Variable	Question and instruction	Response options
	efficiency?'	98 = Unclear/not specified
Critical Outcome$9B	In the author's words, include the text describing the outcome 'service utilization and efficiency.'	Text<br>98 = Unclear/not specified<br>99 = If Outcom$9A=0
Critical Outcome$9C	Report all outcome data for 'service utilization and efficiency.'	Text<br>98 = Unclear/not specified<br>99 = If Outcom$9A=0
Additional Outcomes	What other outcomes were described or measured? Give details. Include all outcomes relating to stillbirth, childbirth, health system outcomes, and women's views and satisfaction.	Text
Evaluation/study details		
Evaluated	Was the intervention evaluated?	0 = No<br>1 = Yes
Type of Evaluation$1	What type of evaluation was done?	1 = Impact (quantitative change in relevant outcome (use of services))<br>2 = Process (evaluation of intervention implementation)<br>3 = Impact and process<br>4 = Economic<br>5 = Multiple<br>6 = Other<br>98 = Unclear/not specified<br>99 = Not applicable
Type of Evaluation$2	Give details with quotations	Text<br>99 = Not applicable
Study Type$1	What was the design of the study?	1 = Randomized controlled trial<br>2 = Controlled clinical trial<br>3 = Cohort analytic (two groups pre + post)<br>4 = Case-control<br>5 = Cohort (one group pre + post (before and after))<br>6 = Interrupted time series<br>7 = Qualitative<br>8 = Mixed methods<br>9 = Regression analysis<br>10 = Other<br>98 = Unclear/not specified
Study Type$2	Give details with quotations	Text<br>99 = Not applicable
Control/Comparator$1	Control/comparator group included?	0 = No<br>1 = Yes<br>98 = Unclear/not specified
Control/Comparator$2	If Control/Comparator$1 = 1, give details	Text<br>99= Not applicable
Data Type	What type of data were presented	1 = Quantitative

For peer review only - http://bmjopen.bmj.com/site/about/guidelines.xhtml

Variable	Question and instruction	Response options
	in the evaluation findings/results?	2 = Qualitative<br>3 = Both
Longitudinal Data	Were the data longitudinal?	0 = No<br>1 = Yes<br>98 = Unclear/not specified
Intervention Claims$1	What claims are made for the effects of the intervention on the outcome/s relevant to quality of maternal, newborn, and child health services?	1 = Positive<br>2 = Neutral<br>3 = Negative<br>4 = Mixed (give details)<br>5 = Other (give details)<br>98 = Unclear/not specified
Intervention Claims$2	Give details. Use author's words (with quotation marks).	Text<br>99 = Not applicable
Strategy Effectiveness	Copy any text about barriers/enablers to the intervention, factors influencing its delivery.	Text
Cost Data	Extract all data on the cost of the intervention and cost compared to outcomes.	Text
Mechanisms	Did the study discuss or propose any mechanisms for engaging the private sector in planning, delivering, and demonstrating accountability for quality maternal, newborn, and child health services? If so, give details and use the author's words (with quotation marks).	Text
Context	In what context was the study conducted? Give details (e.g. regulatory environment, collaborative service delivery models).	Text
Notes	Include any notes you think are relevant	Text
Quality assessment (for quantitative studies): Reviewer 1		
Selection Bias$1	Are the individuals selected to participate in the study likely to be representative of the target population?	1 = Very likely<br>2 = Somewhat likely<br>3 = Not likely<br>98 = Unclear/not specified
Selection Bias$2	What percentage of selected individuals agreed to participate?	1 = 80-100% agreement<br>2 = 60-79% agreement<br>3 = Less than 60% agreement<br>98 = Unclear/not specified<br>99 = Not applicable

For peer review only - http://bmjopen.bmj.com/site/about/guidelines.xhtml

Variable	Question and instruction	Response options
Selection Bias$3	Rate this section (see dictionary[3])	1 = Strong<br>2 = Moderate<br>3 = Weak
Study Design$1	Was the study described as randomized? (Check Study Type$1)<br><br>If NO, go to Confounders$1.	0 = No<br>1 = Yes
Study Design$2	If YES, was the method of randomization described? (see dictionary)	0 = No<br>1 = Yes
Study Design$3	If YES, was the method appropriate? (see dictionary)	0 = No<br>1 = Yes
Study Design$4	Rate this section (see dictionary)	1 = Strong<br>2 = Moderate<br>3 = Weak
Confounders$1	Were there important differences between groups prior to the intervention?<br><br>The following are examples of confounders:<br>1. Race<br>2. Sex<br>3. Marital status/family<br>4. Age<br>5. SES (income or class)<br>6. Education<br>7. Health status<br>Pre-intervention score on outcome measure	0 = No<br>1 = Yes<br>98 = Unclear/not specified
Confounders$2	If YES, indicate the percentage of relevant confounders that were controlled (either in the design (e.g. stratification, matching) or analysis)?	1 = 80-100% (most)<br>2 = 60-79% (some)<br>3 = Less than 60% (few or none)<br>98 = Unclear/not specified<br>99 = Confou$1=0 or 98
Confounders$3	Rate this section (see dictionary)	1 = Strong<br>2 = Moderate<br>3 = Weak
Blinding$1	Was (were) the outcome assessor(s) aware of the information or exposure status of participants?	0 = No<br>1 = Yes<br>98 = Unclear/not specified
Blinding$2	Were the study participants aware of the research question?	0 = No<br>1 = Yes<br>98 = Unclear/not specified
Blinding$3	Rate this section (see dictionary)	1 = Strong

[3] Follow EPHPP's 'Quality Assessment Tool for Quantitative Studies Dictionary' to score study quality for quantitative studies.

For peer review only - http://bmjopen.bmj.com/site/about/guidelines.xhtml

Variable	Question and instruction	Response options
		2 = Moderate<br>3 = Weak
Data Collection Methods$1	Were data collection tools shown to be valid?	0 = No<br>1 = Yes<br>98 = Unclear/not specified
Data Collection Methods$2	Were data collection tools shown to be reliable?	0 = No<br>1 = Yes<br>98 = Unclear/not specified
Data Collection Methods$3	Rate this section (see dictionary)	1 = Strong<br>2 = Moderate<br>3 = Weak
Withdrawals and Dropouts$1	Were withdrawals and dropouts reported in terms of numbers and/or reasons per group?	0 = No<br>1 = Yes<br>98 = Unclear/not specified<br>99 = Not applicable
Withdrawals and Dropouts$2	Indicate the percentage of participants completing the study. (If the percentage differs by groups, record the lowest.)	1 = 80-100%<br>2 = 60-79%<br>3 = Less than 60%<br>98 = Unclear/not specified<br>99 = Not applicable
Withdrawals and Dropouts$3	Rate this section (see dictionary)	1 = Strong<br>2 = Moderate<br>3 = Weak<br>99 = Not applicable
Intervention Integrity$1	What percentage of participants received the allocated intervention or exposure of interest?	1 = 80-100%<br>2 = 60-79%<br>3 = Less than 60%<br>98 = Unclear/not specified
Intervention Integrity$2	Was the consistency of the intervention measured?	0 = No<br>1 = Yes<br>98 = Unclear/not specified
Intervention Integrity$3	Is it likely that the subjects received an unintended intervention (contamination or co-intervention) that may influence the results?	0 = No<br>1 = Yes<br>98 = Unclear/not specified
Analyses$1	Indicate the unit of allocation.	1 = Community<br>2 = Organization/institution<br>3 = Practice/office<br>4 = Individual
Analyses$2	Indicate the unit of analysis.	1 = Community<br>2 = Organization/institution<br>3 = Practice/office<br>4 = Individual
Analyses$3	Are the statistical methods appropriate for the study design?	0 = No<br>1 = Yes<br>98 = Unclear/not specified
Analyses$4	Is the analysis performed by intervention allocation status (i.e.	0 = No<br>1 = Yes

For peer review only - http://bmjopen.bmj.com/site/about/guidelines.xhtml

Variable	Question and instruction	Response options
	intention to treat) rather than the actual intervention received?	98 = Unclear/not specified
Global Rating$1	Based on the final section ratings for selection bias, study design, confounders, blinding, data collection methods, and withdrawals and dropouts, what is the global rating for this paper?	1 = Strong (no WEAK ratings)<br>2 = Moderate (one WEAK rating)<br>3 = Weak (two or more WEAK ratings)
Global Rating$2	With both reviewers discussing the ratings, is there a discrepancy between the two reviewers with respect to the following sections: for selection bias, study design, confounders, blinding, data collection methods, and withdrawals and dropouts	0 = No<br>1 = Yes
Global Rating$3	If YES, indicate the reason for the discrepancy.	1 = Oversight<br>2 = Differences in interpretation of criteria<br>3 = Differences in interpretation of study
Global Rating$4	Final decision of both reviewers	1 = Strong<br>2 = Moderate<br>3 = Weak
Quality assessment (for quantitative studies): Reviewer 2		
Selection Bias$4	Are the individuals selected to participate in the study likely to be representative of the target population?	1 = Very likely<br>2 = Somewhat likely<br>3 = Not likely<br>98 = Unclear/not specified
Selection Bias$5	What percentage of selected individuals agreed to participate?	1 = 80-100% agreement<br>2 = 60-79% agreement<br>3 = Less than 60% agreement<br>98 = Unclear/not specified<br>99 = Not applicable
Selection Bias$6	Rate this section (see dictionary[4])	1 = Strong<br>2 = Moderate<br>3 = Weak
Study Design$5	Was the study described as randomized? (Check Study Type$1)<br><br>If NO, go to Confounders$4.	0 = No<br>1 = Yes
Study Design$6	If YES, was the method of randomization described? (see dictionary)	0 = No<br>1 = Yes
Study Design$7	If YES, was the method appropriate? (see dictionary)	0 = No<br>1 = Yes

[4] Follow EPHPP's 'Quality Assessment Tool for Quantitative Studies Dictionary' to score study quality for quantitative studies.

For peer review only - http://bmjopen.bmj.com/site/about/guidelines.xhtml

Variable	Question and instruction	Response options
Study Design$8	Rate this section (see dictionary)	1 = Strong<br>2 = Moderate<br>3 = Weak
Confounders$4	Were there important differences between groups prior to the intervention?<br><br>The following are examples of confounders:<br>8. Race<br>9. Sex<br>10. Marital status/family<br>11. Age<br>12. SES (income or class)<br>13. Education<br>14. Health status<br>Pre-intervention score on outcome measure	0 = No<br>1 = Yes<br>98 = Unclear/not specified
Confounders$5	If YES, indicate the percentage of relevant confounders that were controlled (either in the design (e.g. stratification, matching) or analysis)?	1 = 80-100% (most)<br>2 = 60-79% (some)<br>3 = Less than 60% (few or none)<br>98 = Unclear/not specified<br>99 = Confou$1=0 or 98
Confounders$6	Rate this section (see dictionary)	1 = Strong<br>2 = Moderate<br>3 = Weak
Blinding$4	Was (were) the outcome assessor(s) aware of the information or exposure status of participants?	0 = No<br>1 = Yes<br>98 = Unclear/not specified
Blinding$5	Were the study participants aware of the research question?	0 = No<br>1 = Yes<br>98 = Unclear/not specified
Blinding$6	Rate this section (see dictionary)	1 = Strong<br>2 = Moderate<br>3 = Weak
Data Collection Methods$4	Were data collection tools shown to be valid?	0 = No<br>1 = Yes<br>98 = Unclear/not specified
Data Collection Methods$5	Were data collection tools shown to be reliable?	0 = No<br>1 = Yes<br>98 = Unclear/not specified
Data Collection Methods$6	Rate this section (see dictionary)	1 = Strong<br>2 = Moderate<br>3 = Weak
Withdrawals and Dropouts$4	Were withdrawals and dropouts reported in terms of numbers and/or reasons per group?	0 = No<br>1 = Yes<br>98 = Unclear/not specified<br>99 = Not applicable

For peer review only - http://bmjopen.bmj.com/site/about/guidelines.xhtml

Variable	Question and instruction	Response options
Withdrawals and Dropouts$5	Indicate the percentage of participants completing the study. (If the percentage differs by groups, record the lowest.)	1 = 80-100%<br>2 = 60-79%<br>3 = Less than 60%<br>98 = Unclear/not specified<br>99 = Not applicable
Withdrawals and Dropouts$6	Rate this section (see dictionary)	1 = Strong<br>2 = Moderate<br>3 = Weak<br>99 = Not applicable
Intervention Integrity$4	What percentage of participants received the allocated intervention or exposure of interest?	1 = 80-100%<br>2 = 60-79%<br>3 = Less than 60%<br>98 = Unclear/not specified
Intervention Integrity$5	Was the consistency of the intervention measured?	0 = No<br>1 = Yes<br>98 = Unclear/not specified
Intervention Integrity$6	Is it likely that the subjects received an unintended intervention (contamination or co-intervention) that may influence the results?	0 = No<br>1 = Yes<br>98 = Unclear/not specified
Analyses$5	Indicate the unit of allocation.	1 = Community<br>2 = Organization/institution<br>3 = Practice/office<br>4 = Individual
Analyses$6	Indicate the unit of analysis.	1 = Community<br>2 = Organization/institution<br>3 = Practice/office<br>4 = Individual
Analyses$7	Are the statistical methods appropriate for the study design?	0 = No<br>1 = Yes<br>98 = Unclear/not specified
Analyses$8	Is the analysis performed by intervention allocation status (i.e. intention to treat) rather than the actual intervention received?	0 = No<br>1 = Yes<br>98 = Unclear/not specified
Global Rating$5	Based on the final section ratings for selection bias, study design, confounders, blinding, data collection methods, and withdrawals and dropouts, what is the global rating for this paper?	1 = Strong (no WEAK ratings)<br>2 = Moderate (one WEAK rating)<br>3 = Weak (two or more WEAK ratings)
Quality assessment (for qualitative studies): Reviewer 1		
Scope and Purpose$1	Does the study have a clear statement of, and rationale for, its research question/aims/purposes?<br><br>Specific prompts:<br>Clarity of focus demonstrated	0 = No<br>1 = Yes

For peer review only - http://bmjopen.bmj.com/site/about/guidelines.xhtml

Variable	Question and instruction	Response options
	▯ Explicit purpose given, such as descriptive/explanatory intent, theory building, hypothesis testing<br>▯ Link between research and existing knowledge demonstrated	
Scope and Purpose$2	Is the study thoroughly contextualized by existing literature?<br><br>Specific prompt:<br>▯ Evidence of systematic approach to literature review, location of literature to contextualize the findings, or both	0 = No<br>1 = Yes
Scope and Purpose$3	Rate this section.<br><br>When Purpose$1 and Purpose$2 = 1, then Purpose$3 = 1. When Purpose$1 or Purpose$2 = 0, then Purpose$3 = 2. When Purpose$1 and Purpose$2 = 0, then Purpose$3 = 3.	1 = Strong<br>2 = Moderate<br>3 = Weak
Design$1	Is the method/design apparent and consistent with the research intent?<br><br>Specific prompts:<br>▯ Rationale given for use of qualitative design<br>▯ Discussion of epistemological/ontological grounding<br>▯ Rationale explored for specific qualitative method (e.g. ethnography, grounded theory, phenomenology)<br>▯ Discussion of why particular method chosen is most appropriate/sensitive/relevant for research question/aims<br>▯ Setting appropriate	0 = No<br>1 = Yes
Design$2	Is the data collection strategy apparent and appropriate?<br><br>Specific prompts:<br>▯ Were data collection methods appropriate for type of data required and for specific qualitative method?<br>▯ Were they likely to capture the	0 = No<br>1 = Yes

For peer review only - http://bmjopen.bmj.com/site/about/guidelines.xhtml

For peer review only

Variable	Question and instruction	Response options
	complexity/diversity of experience and illuminate context in sufficient detail?<br>Was triangulation of data sources used if appropriate?	
Design$3	Rate this section<br><br>When Design$1 and Design$2 = 1, then Design$3 = 1. When Design$1 or Design$2 = 0, then Design$3 = 2. When Design$1 and Design$2 = 0, then Design$3 = 3.	1 = Strong<br>2 = Moderate<br>3 = Weak
Sampling Strategy$1	Are the sampling and sampling method appropriate?<br><br>Specific prompts:<br>Selection criteria detailed, and description of how sampling was undertaken<br>Justification for sampling strategy given<br>Thickness of description likely to be achieved from sampling<br>Any disparity between planned and actual sample explained	0 = No<br>1 = Yes
Sampling Strategy$2	Rate this section<br><br>When Strategy$1 = 1, then Strategy$2 = 1. When Strategy$1 = 0, then Strategy$2 = 3. At the reviewer's discretion, Strategy$2 may be rated as 2, if the study met most but not all of the prompts.	1 = Strong<br>2 = Moderate<br>3 = Weak
Analysis$1	Is the analytical approach appropriate?<br><br>Specific prompts:<br>Approach made explicit (e.g. Thematic distillation, constant comparative method, grounded theory)<br>Was it appropriate for the qualitative method chosen?<br>Was data managed by software package or by hand and why?<br>Discussion of how coding systems/conceptual frameworks evolved<br>How was context of data retained during analysis	0 = No<br>1 = Yes

For peer review only - http://bmjopen.bmj.com/site/about/guidelines.xhtml

Variable	Question and instruction	Response options
	▯ Evidence that the subjective meanings of participants were portrayed<br>▯ Evidence of more than one researcher involved in stages if appropriate to epistemological/theoretical stance<br>▯ Did research participants have any involvement in analysis (e.g. member checking)<br>▯ Evidence provided that data reached saturation or discussion/rationale if it did not<br>▯ Evidence that deviant data was sought, or discussion/rationale if it was not	
Analysis$2	Rate this section<br><br>When Analysis$1 = 1, then Analysis$2 = 1. When Analysis$1 = 0, then Analysis$2 = 3. At the reviewer's discretion, Analysis$2 may be rated as 2, if the study met most but not all of the prompts.	1 = Strong<br>2 = Moderate<br>3 = Weak
Interpretation$1	Is the context described and taken account of in the interpretation?<br><br>Specific prompts:<br>▯ Description of social/physical and interpersonal contexts of data collection<br>▯ Evidence that researcher spent time 'dwelling with the data', interrogating it for competing/alternative explanations of phenomena	0 = No<br>1 = Yes
Interpretation$2	Is a clear audit trail given?<br><br>Specific prompt:<br>▯ Sufficient discussion of research processes such that others can follow the 'decision trail'	0 = No<br>1 = Yes
Interpretation$3	Are data used to support interpretation?<br><br>Specific prompts:<br>▯ Extensive use of field notes entries/verbatim interview quotes in discussion of findings<br>▯ Clear exposition of how	0 = No<br>1 = Yes

For peer review only - http://bmjopen.bmj.com/site/about/guidelines.xhtml

Variable	Question and instruction	Response options
	interpretation led to conclusions	
Interpretation$4	Rate this section<br><br>When Interpretation$1, Interpretation$2, and Interpretation$3 = 1, then Interpretation$4 = 1. When one response to Interpretation$1—Interpretation$3 = 0, then Interpretation$4 = 2. When two or more responses to Interpretation$1—Interpretation$3 = 0, then Interpretation$4 = 3.	1 = Strong<br>2 = Moderate<br>3 = Weak
Reflexivity$1	Is researcher reflexivity demonstrated?<br><br>Specific prompts:<br>▯ Discussion of relationship between researcher and participants during fieldwork<br>▯ Demonstration of researcher's influence on stages of research process<br>▯ Evidence of self-awareness/insight<br>▯ Documentation of effects of the research on researcher<br>▯ Evidence of how problems/complications met were dealt with	0 = No<br>1 = Yes
Reflexivity$2	Rate this section<br><br>When Reflexivity$1 = 1, then Reflexivity$2 = 1. When Reflexivity$1 = 0, then Reflexivity$2 = 3. At the reviewer's discretion, Reflexivity$2 may be rated as 2, if the study met most but not all of the prompts.	1 = Strong<br>2 = Moderate<br>3 = Weak
Ethical Dimensions$1	Is there demonstration of sensitivity to ethical concerns?<br><br>Specific prompts:<br>▯ Ethical committee approval granted<br>▯ Clear commitment to integrity, honesty, transparency, equality and mutual respect in relationships with participants<br>▯ Evidence of fair dealing with all	0 = No<br>1 = Yes

For peer review only

Variable	Question and instruction	Response options
	research participants<br>⍰ Recording of dilemmas met and how resolved in relation to ethical issues<br>⍰ Documentation of how autonomy, consent, confidentiality, anonymity were managed	
Ethical Dimensions$2	Rate this section<br><br>When Dimensions$1 = 1, then Dimensions$2 = 1. When Dimensions$1 = 0, then Dimensions$2 = 3. At the reviewer's discretion, Dimensions$2 may be rated as 2, if the study met most but not all of the prompts.	1 = Strong<br>2 = Moderate<br>3 = Weak
Relevance and Transferability$1	Are relevance and transferability evident?<br><br>Specific prompts:<br>⍰ Sufficient evidence for typicality specificity to be assessed<br>⍰ Analysis interwoven with existing theories and other relevant explanatory literature drawn from similar settings and studies<br>⍰ Discussion of how explanatory propositions/emergent theory may fit other contexts<br>⍰ Limitations/weaknesses of study clearly outlined<br>⍰ Clearly resonates with other knowledge and experience<br>⍰ Results/conclusions obviously supported by evidence<br>⍰ Interpretation plausible and 'makes sense'<br>⍰ Provides new insights and increases understanding<br>⍰ Significance for current policy and practice outlined<br>⍰ Assessment of value/empowerment for participants<br>⍰ Outlines further directions for investigation<br>⍰ Comment on whether aims/purposes of research were achieved	0 = No<br>1 = Yes

For peer review only

For peer review only - http://bmjopen.bmj.com/site/about/guidelines.xhtml

Variable	Question and instruction	Response options
Relevance and Transferability$2	Rate this section<br><br>Transferability$1 = 1, then Transferability$2 = 1. When Transferability$1 = 0, then Transferability$2 = 3. At the reviewer's discretion, Transferability$2 may be rated as 2, if the study met most but not all of the prompts.	1 = Strong<br>2 = Moderate<br>3 = Weak
Global Rating$1	Based on the final section ratings for scope and purpose, design, sampling strategy, analysis, interpretation, reflexivity, ethical dimensions, and relevance and transferability, what is the global rating for this paper?	1 = Strong (no WEAK ratings)<br>2 = Moderate (one WEAK rating)<br>3 = Weak (two or more WEAK ratings)
Global Rating$2	With both reviewers discussing the ratings, is there a discrepancy between the two reviewers with respect to the following sections: scope and purpose, design, sampling strategy, analysis, interpretation, reflexivity, ethical dimensions, and relevance and transferability	0 = No<br>1 = Yes
Global Rating$3	If YES, indicate the reason for the discrepancy.	1 = Oversight<br>2 = Differences in interpretation of criteria<br>3 = Differences in interpretation of study
Global Rating$4	Final decision of both reviewers	1 = Strong<br>2 = Moderate<br>3 = Weak
Quality assessment (for qualitative studies): Reviewer 2		
Scope and Purpose$4	Does the study have a clear statement of, and rationale for, its research question/aims/purposes?<br><br>Specific prompts:<br>▯ Clarity of focus demonstrated<br>▯ Explicit purpose given, such as descriptive/explanatory intent, theory building, hypothesis testing<br>▯ Link between research and existing knowledge demonstrated	0 = No<br>1 = Yes
Scope and Purpose$5	Is the study thoroughly contextualized by existing literature?	0 = No<br>1 = Yes

For peer review only - http://bmjopen.bmj.com/site/about/guidelines.xhtml

Variable	Question and instruction	Response options
	Specific prompt:<br>▯ Evidence of systematic approach to literature review, location of literature to contextualize the findings, or both	
Scope and Purpose$6	Rate this section.<br><br>When Purpose$4 and Purpose$5 = 1, then Purpose$6 = 1. When Purpose$4 or Purpose$5 = 0, then Purpose$6 = 2. When Purpose$4 and Purpose$5 = 0, then Purpose$6 = 3.	1 = Strong<br>2 = Moderate<br>3 = Weak
Design$4	Is the method/design apparent and consistent with the research intent?<br><br>Specific prompts:<br>▯ Rationale given for use of qualitative design<br>▯ Discussion of epistemological/ontological grounding<br>▯ Rationale explored for specific qualitative method (e.g. ethnography, grounded theory, phenomenology)<br>▯ Discussion of why particular method chosen is most appropriate/sensitive/relevant for research question/aims<br>▯ Setting appropriate	0 = No<br>1 = Yes
Design$5	Is the data collection strategy apparent and appropriate?<br><br>Specific prompts:<br>▯ Were data collection methods appropriate for type of data required and for specific qualitative method?<br>▯ Were they likely to capture the complexity/diversity of experience and illuminate context in sufficient detail?<br>▯▯Was triangulation of data sources used if appropriate?	0 = No<br>1 = Yes
Design$6	Rate this section<br><br>When Design$4 and Design$5 = 1,	1 = Strong<br>2 = Moderate<br>3 = Weak

For peer review only - http://bmjopen.bmj.com/site/about/guidelines.xhtml

Variable	Question and instruction	Response options
	then Design$6 = 1. When Design$4 or Design$5 = 0, then Design$6 = 2. When Design$4 and Design$5 = 0, then Design$6 = 3.	
Sampling Strategy$3	Are the sampling and sampling method appropriate?<br><br>Specific prompts:<br>▯ Selection criteria detailed, and description of how sampling was undertaken<br>▯ Justification for sampling strategy given<br>▯ Thickness of description likely to be achieved from sampling<br>▯ Any disparity between planned and actual sample explained	0 = No<br>1 = Yes
Sampling Strategy$4	Rate this section<br><br>When Strategy$3 = 1, then Strategy$4 = 1. When Strategy$3 = 0, then Strategy$4 = 3. At the reviewer's discretion, Strategy$4 may be rated as 2, if the study met most but not all of the prompts.	1 = Strong<br>2 = Moderate<br>3 = Weak
Analysis$3	Is the analytical approach appropriate?<br><br>Specific prompts:<br>▯ Approach made explicit (e.g. Thematic distillation, constant comparative method, grounded theory)<br>▯ Was it appropriate for the qualitative method chosen?<br>▯ Was data managed by software package or by hand and why?<br>▯ Discussion of how coding systems/conceptual frameworks evolved<br>▯ How was context of data retained during analysis<br>▯ Evidence that the subjective meanings of participants were portrayed<br>▯ Evidence of more than one researcher involved in stages if appropriate to epistemological/theoretical stance<br>▯ Did research participants have	0 = No<br>1 = Yes

For peer review only

Variable	Question and instruction	Response options
	any involvement in analysis (e.g. member checking)<br>▯ Evidence provided that data reached saturation or discussion/rationale if it did not<br>▯ Evidence that deviant data was sought, or discussion/rationale if it was not	
Analysis$4	Rate this section<br><br>When Analysis$3 = 1, then Analysis$4 = 1. When Analysis$3 = 0, then Analysis$4 = 3. At the reviewer's discretion, Analysis$4 may be rated as 2, if the study met most but not all of the prompts.	1 = Strong<br>2 = Moderate<br>3 = Weak
Interpretation$5	Is the context described and taken account of in the interpretation?<br><br>Specific prompts:<br>▯ Description of social/physical and interpersonal contexts of data collection<br>▯ Evidence that researcher spent time 'dwelling with the data', interrogating it for competing/alternative explanations of phenomena	0 = No<br>1 = Yes
Interpretation$6	Is a clear audit trail given?<br><br>Specific prompt:<br>▯ Sufficient discussion of research processes such that others can follow the 'decision trail'	0 = No<br>1 = Yes
Interpretation$7	Are data used to support interpretation?<br><br>Specific prompts:<br>▯ Extensive use of field notes entries/verbatim interview quotes in discussion of findings<br>▯ Clear exposition of how interpretation led to conclusions	0 = No<br>1 = Yes
Interpretation$8	Rate this section<br><br>When Interpretation$5, Interpretation$6, and Interpretation$7 = 1, then Interpretation$8 = 1. When one response to Interpretation$5—	1 = Strong<br>2 = Moderate<br>3 = Weak

For peer review only - http://bmjopen.bmj.com/site/about/guidelines.xhtml

Variable	Question and instruction	Response options
	Interpretation$7 = 0, then Interpretation$8 = 2. When two or more responses to Interpretation$5—Interpretation$7 = 0, then Interpretation$8 = 3.	
Reflexivity$3	Is researcher reflexivity demonstrated?<br><br>Specific prompts:<br>▯ Discussion of relationship between researcher and participants during fieldwork<br>▯ Demonstration of researcher's influence on stages of research process<br>▯ Evidence of self-awareness/insight<br>▯ Documentation of effects of the research on researcher<br>▯ Evidence of how problems/complications met were dealt with	0 = No<br>1 = Yes
Reflexivity$4	Rate this section<br><br>When Reflexivity$3 = 1, then Reflexivity$4 = 1. When Reflexivity$3 = 0, then Reflexivity$4 = 3. At the reviewer's discretion, Reflexivity$4 may be rated as 2, if the study met most but not all of the prompts.	1 = Strong<br>2 = Moderate<br>3 = Weak
Ethical Dimensions$3	Is there demonstration of sensitivity to ethical concerns?<br><br>Specific prompts:<br>▯ Ethical committee approval granted<br>▯ Clear commitment to integrity, honesty, transparency, equality and mutual respect in relationships with participants<br>▯ Evidence of fair dealing with all research participants<br>▯ Recording of dilemmas met and how resolved in relation to ethical issues<br>▯ Documentation of how autonomy, consent, confidentiality, anonymity were managed	0 = No<br>1 = Yes
Ethical Dimensions$4	Rate this section	1 = Strong

For peer review only - http://bmjopen.bmj.com/site/about/guidelines.xhtml

Variable	Question and instruction	Response options
	When Dimensions$3 = 1, then Dimensions$4 = 1. When Dimensions$3 = 0, then Dimensions$4 = 3. At the reviewer's discretion, Dimensions$4 may be rated as 2, if the study met most but not all of the prompts.	2 = Moderate<br>3 = Weak
Relevance and Transferability$3	Are relevance and transferability evident?<br><br>Specific prompts:<br>? Sufficient evidence for typicality specificity to be assessed<br>? Analysis interwoven with existing theories and other relevant explanatory literature drawn from similar settings and studies<br>? Discussion of how explanatory propositions/emergent theory may fit other contexts<br>? Limitations/weaknesses of study clearly outlined<br>? Clearly resonates with other knowledge and experience<br>? Results/conclusions obviously supported by evidence<br>? Interpretation plausible and 'makes sense'<br>? Provides new insights and increases understanding<br>? Significance for current policy and practice outlined<br>? Assessment of value/empowerment for participants<br>? Outlines further directions for investigation<br>? Comment on whether aims/purposes of research were achieved	0 = No<br>1 = Yes
Relevance and Transferability$4	Rate this section<br><br>Transferability$3 = 1, then Transferability$4 = 1. When Transferability$3 = 0, then Transferability$4 = 3. At the reviewer's discretion, Transferability$4 may be rated as	1 = Strong<br>2 = Moderate<br>3 = Weak

For peer review only

For peer review only - http://bmjopen.bmj.com/site/about/guidelines.xhtml

Variable	Question and instruction	Response options
	2, if the study met most but not all of the prompts.	
Global Rating$5	Based on the final section ratings for scope and purpose, design, sampling strategy, analysis, interpretation, reflexivity, ethical dimensions, and relevance and transferability, what is the global rating for this paper?	1 = Strong (no WEAK ratings)<br>2 = Moderate (one WEAK rating)<br>3 = Weak (two or more WEAK ratings)

For peer review only

PRISMA-P (Preferred Reporting Items for Systematic review and Meta-Analysis Protocols) 2015 checklist: recommended items to address in a systematic review protocol*

Section and topic	Item	Checklist item	Reported on page
**ADMINISTRATIVE INFORMATION**			
Title:			
Identification	1a	Identify the report as a protocol of a systematic review	1
Update	1b	If the protocol is for an update of a previous systematic review, identify as such	Not applicable
Registration	2	If registered, provide the name of the registry (such as PROSPERO) and registration number	12
Authors:			
Contact	3a	Provide name, institutional affiliation, e-mail address of all protocol authors; provide physical mailing address of corresponding author	1
Contributions	3b	Describe contributions of protocol authors and identify the guarantor of the review	12
Amendments	4	If the protocol represents an amendment of a previously completed or published protocol, identify as such and list changes; otherwise, state plan for documenting important protocol amendments	12
Support:			
Sources	5a	Indicate sources of financial or other support for the review	12
Sponsor	5b	Provide name for the review funder and/or sponsor	12
Role of sponsor or funder	5c	Describe roles of funder(s), sponsor(s), and/or institution(s), if any, in developing the protocol	12
**INTRODUCTION**			
Rationale	6	Describe the rationale for the review in the context of what is already known	4
Objectives	7	Provide an explicit statement of the question(s) the review will address with reference to participants, interventions, comparators, and outcomes (PICO)	4-5
**METHODS**			
Eligibility criteria	8	Specify the study characteristics (such as PICO, study design, setting, time frame) and report characteristics (such as years considered, language, publication status) to be used as criteria for eligibility for the review	5-6

For peer review only

Information sources	9	Describe all intended information sources (such as electronic databases, contact with study authors, trial registers or other grey literature sources) with planned dates of coverage	6
Search strategy	10	Present draft of search strategy to be used for at least one electronic database, including planned limits, such that it could be repeated	8-11
Study records:			
Data management	11a	Describe the mechanism(s) that will be used to manage records and data throughout the review	7
Selection process	11b	State the process that will be used for selecting studies (such as two independent reviewers) through each phase of the review (that is, screening, eligibility and inclusion in meta-analysis)	7
Data collection process	11c	Describe planned method of extracting data from reports (such as piloting forms, done independently, in duplicate), any processes for obtaining and confirming data from investigators	7
Data items	12	List and define all variables for which data will be sought (such as PICO items, funding sources), any pre-planned data assumptions and simplifications	7, Annexes A-B
Outcomes and prioritization	13	List and define all outcomes for which data will be sought, including prioritization of main and additional outcomes, with rationale	5-7, Annexes A-B
Risk of bias in individual studies	14	Describe anticipated methods for assessing risk of bias of individual studies, including whether this will be done at the outcome or study level, or both; state how this information will be used in data synthesis	7-8
Data synthesis	15a	Describe criteria under which study data will be quantitatively synthesised	8
	15b	If data are appropriate for quantitative synthesis, describe planned summary measures, methods of handling data and methods of combining data from studies, including any planned exploration of consistency (such as I^2, Kendall's τ)	8
	15c	Describe any proposed additional analyses (such as sensitivity or subgroup analyses, meta-regression)	8
	15d	If quantitative synthesis is not appropriate, describe the type of summary planned	8
Meta-bias(es)	16	Specify any planned assessment of meta-bias(es) (such as publication bias across studies, selective reporting within studies)	8
Confidence in cumulative evidence	17	Describe how the strength of the body of evidence will be assessed (such as GRADE)	7

*** It is strongly recommended that this checklist be read in conjunction with the PRISMA-P Explanation and Elaboration (cite when available) for important clarification on the items. Amendments to a review protocol should be tracked and dated. The copyright for PRISMA-P (including checklist) is held by the PRISMA-P Group and is distributed under a Creative Commons Attribution Licence 4.0.**

From: Shamseer L, Moher D, Clarke M, Ghersi D, Liberati A, Petticrew M, Shekelle P, Stewart L, PRISMA-P Group. Preferred reporting items for systematic review and meta-analysis protocols (PRISMA-P) 2015: elaboration and explanation. BMJ. 2015 Jan 2;349(jan02 1):g7647.

For peer review only

BMJ Open

Private sector delivery of quality care for maternal, newborn, and child health in low- and middle-income countries: A mixed-methods systematic review protocol

Journal:	*BMJ Open*
Manuscript ID	bmjopen-2019-033141.R1
Article Type:	Protocol
Date Submitted by the Author:	04-Oct-2019
Complete List of Authors:	Lattof, Samantha; World Health Organization, Department of Maternal, Newborn, Child and Adolescent Health<br>Maliqi, Blerta; World Health Organization, Department of Maternal, Newborn, Child and Adolescent Health
**Primary Subject Heading**:	Global health
Secondary Subject Heading:	Health services research, Obstetrics and gynaecology, Paediatrics, Public health, Research methods
Keywords:	private sector, maternal health, newborn health, child health, Quality in health care < HEALTH SERVICES ADMINISTRATION & MANAGEMENT, systematic review

SCHOLARONE™
Manuscripts

I, the Submitting Author has the right to grant and does grant on behalf of all authors of the Work (as defined in the below author licence), an exclusive licence and/or a non-exclusive licence for contributions from authors who are: i) UK Crown employees; ii) where BMJ has agreed a CC-BY licence shall apply, and/or iii) in accordance with the terms applicable for US Federal Government officers or employees acting as part of their official duties; on a worldwide, perpetual, irrevocable, royalty-free basis to BMJ Publishing Group Ltd ("BMJ") its licensees and where the relevant Journal is co-owned by BMJ to the co-owners of the Journal, to publish the Work in this journal and any other BMJ products and to exploit all rights, as set out in our licence.

The Submitting Author accepts and understands that any supply made under these terms is made by BMJ to the Submitting Author unless you are acting as an employee on behalf of your employer or a postgraduate student of an affiliated institution which is paying any applicable article publishing charge ("APC") for Open Access articles. Where the Submitting Author wishes to make the Work available on an Open Access basis (and intends to pay the relevant APC), the terms of reuse of such Open Access shall be governed by a Creative Commons licence – details of these licences and which Creative Commons licence will apply to this Work are set out in our licence referred to above.

Other than as permitted in any relevant BMJ Author's Self Archiving Policies, I confirm this Work has not been accepted for publication elsewhere, is not being considered for publication elsewhere and does not duplicate material already published. I confirm all authors consent to publication of this Work and authorise the granting of this licence.

For peer review only

Title

Private sector delivery of quality care for maternal, newborn, and child health in low- and middle-income countries: A mixed-methods systematic review protocol

Authors

Samantha R. Lattof, PhD; Corresponding author; Department of Maternal, Newborn, Child and Adolescent Health; World Health Organization; Avenue Appia 20, 1202 Geneva, Switzerland; lattofs@who.int

Blerta Maliqi, MD; Department of Maternal, Newborn, Child and Adolescent Health; World Health Organization; Avenue Appia 20, 1202 Geneva, Switzerland; maliqib@who.int

Keywords

private sector, maternal health, newborn health, child health, quality of care, systematic review, protocols & guidelines

Word count

3,344 words

For peer review only

Abstract

Introduction

To accelerate progress to reach the Sustainable Development Goals for ending preventable maternal, newborn, and child deaths, it is critical that both the public and private health service delivery systems invest in increasing coverage of interventions to sustainably deliver quality care for mothers, newborns, and children at scale. Although various approaches have been successful in high-income countries, little is known about how to effectively engage and sustain private sector involvement in delivering quality care in low- and middle-income countries. Our systematic review will examine private sector implementation of quality care for maternal, newborn, and child health (MNCH) and the impact of this care. This protocol details our intended methodological and analytical approaches, based on the Preferred Reporting Items for Systematic reviews and Meta-Analyses (PRISMA) reporting guideline for protocols.

Methods and analysis

Following the PRISMA approach, this systematic review will include quantitative, qualitative, and mixed-methods studies addressing the provision of quality MNCH care by private sector providers. Eight databases (Cumulative Index to Nursing and Allied Health, EconLit, Excerpta Medica Database, International Bibliography of the Social Sciences, Popline, PubMed, ScienceDirect, Web of Science) and two websites will be searched for relevant studies published between 1 January 1995 and 30 June 2019. For inclusion, studies in low- and middle-income countries must examine at least one of the following critical outcomes: maternal morbidity or mortality; newborn morbidity or mortality; child morbidity or mortality; quality of care; experience of care; and service utilization. Depending on the data, analyses could include meta-analysis, descriptive quantitative statistics, narrative synthesis, and thematic synthesis. Quality will be assessed using tools for qualitative and quantitative studies.

Ethics and dissemination

Formal ethical approval is not required for this research, as the secondary data are not identifiable. Findings from this review will be used to develop models for effective collaboration of the private and public sectors in implementing quality of care for MNCH. In addition to publishing our findings in a peer-reviewed journal, the findings will be shared through the Quality of Care Network, relevant mailing lists, webinars, and social media.

For peer review only

Strengths and limitations of this study

- This mixed-methods systematic review protocol is the first to examine private sector delivery of quality maternal, newborn, and child health care in low- and middle-income countries.
- By using the PRISMA checklist, we increase the potential usefulness, clarity, and transparency with which we will report the results from this study.
- To minimize potential bias from the publication of positive results and to increase the validity of this systematic review, we will make a concerted effort to supplement our electronic database searches with grey literature and programmatic reports.
- We acknowledge a risk of bias in locating studies, as it is possible evidence on the provision of quality health care by the private sector may be disseminated internally beyond public reach.
- For transparency and quality, the protocol includes a codebook and data extraction template.

For peer review only

Introduction

The private sector plays a key role in delivering sexual and reproductive health services. It provides a substantial proportion of family planning services among women aged 15-49 years in Asia (45%), Latin America and the Caribbean (44%), and sub-Saharan Africa (28%).[1] In certain countries, like Democratic Republic of Congo and Nigeria, the private sector's family planning market share exceeds 60%.[2] One in five births in low- and middle-income countries occurred with care delivered by the private sector.[3] Despite the private sector's expanding role in many countries, the quality of services varies. To accelerate progress to reach the Sustainable Development Goals for ending preventable maternal, newborn, and child deaths, it is critical that both the public and private health service delivery systems invest in increasing coverage of interventions to sustainably deliver quality care at scale.

Amongst countries in the Network for Improving Quality of Care for Maternal, Newborn and Child Health (the Network), the private sector addresses an increasing volume of maternal, newborn, and child health (MNCH) care needs. The Network, a consortium of eleven countries and their technical partners, aims to halve maternal and newborn deaths and stillbirths in health facilities in five years' time.[4] Whilst the Network's efforts to achieve this ambitious goal have largely focused on strengthening the public health sector, members of the Network recognize that private providers (i.e., non-government providers, for-profit businesses) are an important source of health care and have a role to play in improving quality care. However, little is known about how to effectively engage and sustain private sector involvement in delivering quality care in low and middle-income countries. This gap must be addressed, if the Network is to achieve its aims of reducing maternal and newborn deaths and stillbirths.

The engagement and contribution of the private sector in implementing quality care standards, developing and identifying best practices for delivering quality MNCH care, and strengthening health systems for delivering quality are areas of great potential that require immediate attention. There is a need to understand what can be done to create, to nurture, and to encourage a vibrant private sector that is fully engaged in improving and sustaining quality of care for mothers, newborns, and children. We aim to begin filling these knowledge gaps by conducting a systematic review that addresses the following research questions:

Primary research questions

1. How and to what extent does the provision of quality health care by the private sector affect morbidity and mortality among mothers, newborns, and children?
2. How and to what extent does provision of quality health care by the private sector affect utilization of services by mothers, newborns, and children?
3. How effective and efficient is the private sector at delivering quality of care?
4. Among mothers, newborns, and children utilizing health care provided by the private sector, what are their experiences of care?

Secondary research questions

For peer review only

5. What mechanisms[a] exist for engaging the private sector[b,5] in delivering quality maternal, newborn, and child health services?
6. What types of regulatory and collaborative service delivery models[c] exist between the public and private sectors to deliver quality of care?

This protocol details our intended methodological and analytical approaches for the systematic review, based on the Preferred Reporting Items for Systematic reviews and Meta-Analyses (PRISMA) reporting guideline for protocols.[6]

Methods and analysis

This systematic review will utilize the PRISMA checklist for reporting systematic reviews and meta-analyses so as to ensure clarity and transparency.[7]

Inclusion/exclusion criteria

This review focuses on studies examining the private sector's involvement in delivering quality health care to pregnant women, mothers, newborns, and children in low- and middle-income countries. Using the World Bank Atlas method for the current 2020 fiscal year, this review will include countries classified as having low-income economies, lower-middle-income economies, and upper-middle-economies.[8] The PICOTS (population, interventions, control, outcomes, timeframe, setting) criteria, detailed in Table 1, provide an overview of our inclusion criteria. Quantitative, qualitative, and mixed-methods studies in low- and middle-income countries will be considered, provided that they report on at least one of the following critical outcomes:

- Maternal morbidity or mortality
- Newborn morbidity or mortality
- Child morbidity or mortality
- Components of quality of care, defined by WHO as "the extent to which health care services provided to individuals and patient populations improve desired health outcomes. In order to achieve this, health care must be safe, effective, timely, efficient, equitable and people-centred," (p. 1046).[9]
- Experience of care, including respectful care
- Service utilization

Journal articles and grey literature (e.g., reports, book chapters), both peer-reviewed and non-peer-reviewed, will be eligible for inclusion. In recognition of the rapid increase in

[a] These mechanisms may allow for demonstrating accountability for quality MNCH services; developing, implementing, and sharing MNCH standards and quality improvement approaches, developing and sharing quality of care implementation packages for MNCH, and developing and sharing policies and plans for quality MNCH.

[b] WHO recognizes three broad categories of private sector engagement: (1) including private actors in the development of public health policy and the development of ownership and contracting arrangements; (2) influencing private sector behaviour through regulatory and financing policy tools; and (3) assigning "private attributes" to public sector organizations (e.g., by giving them managerial autonomy and exposing them to market forces and incentives).[5]

[c] These models may include the private sector's involvement in national MNCH quality of care structures and coordinating mechanisms.

public-private collaborations for health during the late 1990s,[10] items must be published from 1 January 1995 to 30 June 2019. Items will be eligible if published in English, French, German, or Italian. For inclusion, quality must be a specific focus amongst the interventions being implemented, such as quality assessment, quality improvement, clinical quality, perceived quality, or the delivery of quality care. Interventions addressing universal health coverage will also be considered as focusing on quality, since quality health services are a fundamental feature of universal health coverage.[11]

Table 1. PICOTS criteria used in the systematic review

**PICOTS**	
Populations	Pregnant women, mothers, newborns, and children
Interventions	Delivery of quality maternal, newborn, and/or child health services by the private sector
Control	Not necessary
Outcomes	Quantitative, qualitative, or mixed-methods data on:<br>- Maternal morbidity or mortality<br>- Newborn morbidity or mortality<br>- Child morbidity or mortality<br>- Components of quality of care (i.e. safety, effectiveness, timeliness, efficiency, equity, people-centred care)<br>- Experience of care, including respectful care<br>- Service utilization
Timeframe	1 January 1995 to 30 June 2019
Setting	Low- and middle-income countries

As we are focused on service delivery, we are limiting the private sector to providers who deliver direct medical care (e.g., private health facilities, private health providers, civil society organizations delivering care, charities delivering care). Thus, private sector entities that do not deliver direct medical care will be excluded. For example, we will exclude private organizations offering social service support to orphaned children, private pharmaceutical providers (including pharmacies), and private health insurance companies.

We will exclude studies reporting on aggregated service delivery data (i.e., public sector and private sector outcome data combined), as we are only interested in outcomes resulting from service delivery by the private sector. Studies addressing allied fields like family planning, malaria, and HIV/AIDS will be excluded unless they include specific MNCH interventions (e.g., postpartum family planning, integration of family planning into MCH services, abortion services, treatment of malaria in children, prevention of mother-to-child transmission of HIV) as a specific focus or aim. If commentaries and editorials do not introduce new evidence, then we will exclude them in favour of the original study or studies being discussed. Study protocols will also be excluded.

Search strategy and terms

We will search the following eight electronic databases that were assessed for likely coverage, availability, and relevance of literature on the private sector:

For peer review only - http://bmjopen.bmj.com/site/about/guidelines.xhtml

- Cumulative Index to Nursing and Allied Health (CINAHL)
- EconLit
- Excerpta Medica Database (EMBASE)
- International Bibliography of the Social Sciences (IBSS)
- Popline
- PubMed
- ScienceDirect
- Web of Science

Whilst we considered the LILACS database that includes literature on Latin America and the Caribbean, our test search returned no results. A test search in Open Grey returned one extraneous result, so we excluded that database as well. Based on discussions with experts and an initial assessment of the electronic databases, we suspect that a significant body of literature on the private sector is published as grey literature or programmatic reports. Thus, we will make a concerted effort to supplement our electronic database searches with expert-recommended articles and reports that we will obtain by sending a standardized request for literature to a list of experts and working groups identified in collaboration with colleagues at WHO.

Given our interest in locating grey literature, we will also conduct a targeted search of publications on two websites: Health Care Provider Performance Review (HCPPR) and the Maternal healthcare markets Evaluation Team (MET) at the London School of Hygiene & Tropical Medicine. HCPPR is a database of over 700 studies from a systematic review on the effectiveness of strategies to improve healthcare provider performance in low- and middle-income countries.[12] HCPPR's search strategy included extensive searching of document inventories for unpublished studies. MET conducts multidisciplinary research on the role of public and private health sectors in delivering maternal healthcare. Its website includes both peer-reviewed literature and grey literature. We acknowledge risk of bias in locating studies. Our efforts to locate evidence on the provision of quality health care by the private sector may be limited, specifically when such evidence is only disseminated internally where it is beyond public reach.

Our searches will be conducted using combinations of search terms detailed in Table 2. These terms aim to capture all studies examining the private sector; quality of care; and maternal, newborn, and child health (MNCH). The terms were developed and tested for sensitivity, and they will be adapted to the basic search particulars (e.g., wildcards (*), capacity for complex searches) of each electronic database.

Table 2. Search terms and their combinations

1. Private sector	2. Quality of care	3. MNCH
private sector	quality	matern*
for-profit		pregnan*
for profit		mother*
public-private		newborn*
private enterprise*		infant*
NGO		child*

For peer review only - http://bmjopen.bmj.com/site/about/guidelines.xhtml

non-government*		pediatric*
		paediatric*
		neonat*

Screening process

Searches and application of the inclusion/exclusion criteria will be conducted according to the PRISMA flow approach. Citation abstracts for all items uncovered by our searches will be exported into EndNote for screening. After first removing duplicates, remaining items will be screened for inclusion on the basis of title and abstract (TIAB). If inclusion/exclusion cannot be determined based on the TIAB, then the item will be pushed forward for full-text screening. SL will conduct all TIAB and full-text screening, seeking guidance from BM on items considered borderline or problematic.

Data extraction

The data extraction form (Annex A) will be piloted with five randomly selected studies. Any necessary changes will be discussed amongst the authors prior to full data extraction. Based on the guidance in our codebook (Annex B), SL will extract data for all studies into the data extraction template in Excel. Data will be extracted on the following categories:

- Background information (e.g., author, date, setting, study objective)
- Intervention background information (e.g., implementing agency, geographic level, study population)
- Intervention details (e.g., intervention recipients, nature of intervention, dimensions of quality care)
- Critical outcomes (both quantitative and qualitative):
  - Maternal morbidity or mortality
  - Newborn morbidity or mortality
  - Child morbidity or mortality
  - Quality of care
  - Experience of care, including respectful care
  - Service utilization and efficiency
- Secondary outcome (both quantitative and qualitative):
  - Infant and/or child growth
- Evaluation/study details (e.g., study type, data type, intervention claims, strategy effectiveness, cost data)
- Study quality (qualitative and quantitative)

Study quality and risks of bias will be assessed in duplicate using quality assessment tools for quantitative and qualitative studies. For quantitative studies, we will use the Effective Public Health Practice Project's (EPHPP) quality assessment tool that rates public health studies on selection bias, study design, confounders, blinding, data collection methods, withdrawals and dropouts, intervention integrity, and analysis.[13] The accompanying reviewer's dictionary provides details on how to evaluate each section (i.e., strong, moderate, or weak) with the process resulting in 'excellent' agreement between reviewers on the study's final grade.[13 14] For qualitative studies, we will use Solnes Miltenburg et al.'s quality assessment tool based

For peer review only

on criteria developed by Walsh and Downe.[15 16] This tool rates qualitative studies' scope and purpose, design, sampling strategy, analysis, interpretation, reflexivity, ethical dimensions and relevance and transferability.[16] Like the EPHPP tool, Solnes Miltenburg et al.'s tool involves rating each section as strong, moderate, or weak and then using these ratings to determine the paper's overall quality rating (i.e., strong, moderate, or weak).

Data synthesis

Once the data have been extracted, we will analyse the quantitative and qualitative findings separately. Depending on the quantitative studies and data available in the final inventory, a meta-analysis may be appropriate to examine the outcomes of private sector interventions on the provision of quality MNCH care. Data extracted on intervention details (see Annexes A and B, variables 'Nature of Intervention$1' through 'Nature of Intervention$6') will be reviewed for their suitability in meta-analyses. We will examine the effect of each type of private sector intervention affecting the delivery of care (e.g., introduction or change to on-site support for quality improvement, introduction or change to data systems) on our six critical outcomes. When appropriate, we will conduct meta-analyses and generate forest plots in RevMan 5.3.5 in non-Cochrane mode.[17] For interventions in which the study sample size is large and inter-study heterogeneity is minimal, we will use fixed model effect; otherwise, we will use random effect size.[18] The meta-analysis will be limited to randomized control trials and observational studies rated by the EPHPP tool as strong or moderate quality, and meta-biases of included studies will be assessed using ROBIS.[19] If high heterogeneity exists between study designs, interventions, populations, and definitions, then we will instead present the quantitative findings using a narrative synthesis to report the data thematically with tables of descriptive quantitative statistics and study outcomes.

For the qualitative data, we will enter the verbatim findings into NVivo for a three-step thematic synthesis.[20] Each line of text will initially be coded based on its content and meaning. All codes will be included in a codebook with new codes added when appropriate. The codes will then be related together to develop categories and linkages between the data. After reviewing the data for analytical themes, we will make inferences about the provision of quality maternal, newborn, and child health care by the private sector. We suspect that this type of synthesis will be particularly well suited to data on patients' experiences of care by the private sector and existing mechanisms for engaging the private sector in delivering quality MNCH services.

Patient and public involvement

The design of this systematic review protocol did not involve patients, though we hope the final inventory of studies included in this review will report on patients' experiences of care. The need for this systematic review was initiated by discussions with public sector health officials in Network countries.

Final search strategy by database

The full electronic search strategies for all databases, including limits and filters used, appear below.

For peer review only

CINAHL

Search strategy: We will search all sets of search terms (Table 2).

Search options:

- Search mode: Boolean/phrase
- Limit results:
  - Published date: January 1995 to June 2019
  - Language: English, French, German, Italian

Search terms: ("private sector" OR for-profit OR "for profit" OR public-private OR "private enterprise*" OR NGO OR non-government*) AND (quality) AND (matern* OR pregnan* OR mother* OR newborn* OR infant* OR child* OR pediatric* OR paediatric* OR neonat*)

EconLit

Search strategy: We will search all sets of search terms (Table 2).

Search options:

- Search mode: Boolean/phrase
- Limit results:
  - Published date: January 1995 to June 2019

Search terms: ("private sector" OR for-profit OR "for profit" OR public-private OR "private enterprise*" OR NGO OR non-government*) AND (quality) AND (matern* OR pregnan* OR mother* OR newborn* OR infant* OR child* OR pediatric* OR paediatric* OR neonat*)

EMBASE

Search strategy: We will search all search terms (Table 2) using the multi-field search in the abstract field.

Search options:

- Limit results:
  - Publication year: 1995 – current
  - Language: English, French, German, Italian

Search terms: (private sector OR for-profit OR for profit OR public-private OR private enterprise* OR NGO OR non-government*) AND (quality) AND (matern* OR pregnan* OR mother* OR newborn* OR infant* OR child* OR pediatric* OR paediatric* OR neonat*)

HCPPR

Search strategy: For this systematic review database on the effectiveness of strategies to improve health care provider performance in low- and middle-income countries, we will obtain a list of all HCPPR's included grey literature directly from the project investigator.

For peer review only

Grey literature on maternal, newborn, and/or child health services will be included for review.

Search options:

- Limit results:
    - Publication year: 1995 – 2019

IBSS

Search strategy: We will search all sets of search terms (Table 2) using the advanced search feature. To minimize extraneous results returned during test searches, searches will be conducted "anywhere except full text." Thus, the searches will use title and abstract, in line with our initial plans for TIAB screening.

Search options:

- Limit results:
    - Publication date: 1 January 1995 – 30 June 2019
    - Language: English, French, German, Italian

Search terms: noft((“private sector” OR for-profit OR “for profit” OR public-private OR “private enterprise*” OR NGO OR non-government*) AND (quality) AND (matern* OR pregnan* OR mother* OR newborn* OR infant* OR child* OR pediatric* OR paediatric* OR neonat*))

MET

Search strategy: On the MET publications page, we will export all documents that have not been published in peer-reviewed journals (e.g., reports, policy briefs). Peer-reviewed publications appear in journals that have been indexed in other electronic databases included in this systematic review.

Popline

Search strategy: We searched a modified set of search terms (Table 2). Since Popline was retired on 1 September 2019, we relied on an earlier search that did not include the search term ‘neonat*.’ The database does not permit users to include wildcards inside quotation marks, so we split “private enterprise*” into two terms: “private enterprise” and “private enterprises.”

Search options:

- Filters:
    - Year: Published since 1995
    - Language: English, French, German, Italian

For peer review only

Search terms: (("private sector" OR for-profit OR "for profit" OR public-private OR "private enterprise" OR "private enterprises" OR NGO OR non-government*) AND (quality) AND (matern* OR pregnan* OR mother* OR newborn* OR infant* OR child* OR pediatric* OR paediatric*))

PubMed

Search strategy: We will search all sets of search terms (Table 2) using the advanced search builder.

Search options:

- Limit results:
  - Publication dates: From 1 January 1995 to 30 June 2019
  - Language: English, French, German, Italian

Search terms: ("private sector" OR for-profit OR "for profit" OR public-private OR "private enterprise*" OR NGO OR non-government*) AND (quality) AND (matern* OR pregnan* OR mother* OR newborn* OR infant* OR child* OR pediatric* OR paediatric* OR neonat*)

ScienceDirect

Search strategy: Since this database does not support wildcards (*) or more than eight Boolean connectors per field, we will search a modified set of search terms (Table 2) using the advanced search feature and limiting keywords to those most widely used. We will split the terms between articles and title, abstract, or keywords, as detailed below.

Search options:

- Limit results:
  - Year(s): 1995-2019

Search terms:

- Find articles with these terms: (maternal OR maternity OR newborn OR child OR children OR childhood OR childbirth OR pregnancy OR pregnant)
- Title, abstract or keywords: (quality) AND ("private sector" OR for-profit OR "for profit" OR public-private OR "private enterprise" OR "private enterprises" OR NGO OR non-governmental)

Web of Science

Search strategy: We will search all sets of search terms (Table 2) using the advanced search feature and topic (TS) field tag.

Search options:

- Limit results:
  - Year(s): 1995-2019
  - Language: English, French, German, Italian

For peer review only

Search terms: ("private sector" OR for-profit OR "for profit" OR public-private OR "private enterprise*" OR NGO OR non-government*) AND (quality) AND (matern* OR pregnan* OR mother* OR newborn* OR infant* OR child* OR pediatric* OR paediatric* OR neonat*)

Ethics and dissemination

Formal ethical approval is not required for this research, as the publically available secondary data are not identifiable.

This systematic review, which we anticipate concluding by 1 June 2020, will provide evidence on different models that can be drawn upon by countries in planning and implementing their national plans and processes for quality of care. Findings from this review will be used to develop models for effective collaboration of the private and public sectors in implementing quality of care for MNCH. In addition to publishing our findings in a peer-reviewed journal, the findings will be shared through the Quality of Care Network, relevant mailing lists, webinars, and social media.

Should we need to amend this protocol following its publication, we will ensure that future publications arising from this protocol provide the date of each amendment, describe the change(s), and report the rationale for the change(s).

Acknowledgements

We wish to thank David Clarke and Wilson Were (WHO) for their contributions to the wider discussions around this research, Moïse Muzigaba (WHO) for his comments on an earlier draft of this manuscript, and Alex Rowe (Centers for Disease Control and Prevention) for sharing information on the HCPPR database, including a list of grey literature references. This protocol has been registered with PROSPERO (CRD42019143383).

Contributors

BM and SL conceived the idea for the review. SL designed and wrote the first draft of the protocol. Both authors contributed to subsequent revisions and approved the protocol prior to its submission. SL is the guarantor.

Funding

This work was supported by MSD for Mothers and the Maternal, Newborn, Child and Adolescent Health Department of the World Health Organization. MSD for Mothers had no role in the design and development of the study protocol or the decision to publish.

For peer review only - http://bmjopen.bmj.com/site/about/guidelines.xhtml

Competing interests

We declare no competing interests.

Data sharing statement

The data extraction workbook and codebook are available as supplementary annexes.

References

1. Ugaz JI, Chatterji M, Gribble JN, et al. Regional trends in the use of short-acting and long-acting contraception accessed through the private and public sectors. *International Journal of Gynecology & Obstetrics* 2015;130(S3):E3-E7. doi: 10.1016/j.ijgo.2015.03.021
2. Dennis ML, Benova L, Owolabi OO, et al. Meeting need vs. sharing the market: a systematic review of methods to measure the use of private sector family planning and childbirth services in sub-Saharan Africa. *BMC health services research* 2018;18(1):699. doi: 10.1186/s12913-018-3514-y [published Online First: 2018/09/12]
3. Benova L, Macleod D, Footman K, et al. Role of the private sector in childbirth care: cross-sectional survey evidence from 57 low- and middle-income countries using Demographic and Health Surveys. *Tropical Medicine & International Health* 2015;20(12):1657-73. doi: 10.1111/tmi.12598
4. Adeniran A, Likaka A, Knutsson A, et al. Leadership, action, learning and accountability to deliver quality care for women, newborns and children. *Bull World Health Organ* 2018;96(3):222-24. doi: 10.2471/BLT.17.197939 [published Online First: 02/05]
5. Clarke D, Paviza A. The private sector, universal health coverage and primary health care. Technical Series on Primary Health Care. Geneva: WHO, 2018.
6. Moher D, Shamseer L, Clarke M, et al. Preferred reporting items for systematic review and meta-analysis protocols (PRISMA-P) 2015 statement. *Systematic Reviews* 2015;4(1):1. doi: 10.1186/2046-4053-4-1
7. Liberati A, Altman DG, Tetzlaff J, et al. The PRISMA statement for reporting systematic reviews and meta-analyses of studies that evaluate healthcare interventions: explanation and elaboration. *BMJ* 2009;339:b2700. doi: 10.1136/bmj.b2700
8. World Bank. World Bank Country and Lending Groups n.d. [Available from: https://datahelpdesk.worldbank.org/knowledgebase/articles/906519 accessed 3 October 2019.
9. Tunçalp Ö, Were WM, MacLennan C, et al. Quality of care for pregnant women and newborns-the WHO vision. *BJOG* 2015;122(8):1045-49. doi: 10.1111/1471-0528.13451 [published Online First: 2015/05/01]
10. Buse K, Walt G. Global public-private partnerships: part I - a new development in health? *WHO Bulletin* 2000;78(4):549-61.
11. WHO, World Bank, OECD. Delivering quality health services: A global imperative for universal health coverage. Geneva: World Health Organization, Organisation for Economic Co-operation and Development, and The World Bank, 2018:1-99.

For peer review only

12. Rowe SY, Peters DH, Holloway KA, et al. A systematic review of the effectiveness of strategies to improve health care provider performance in low- and middle-income countries: Methods and descriptive results. *PLOS ONE* 2019;14(5):e0217617. doi: 10.1371/journal.pone.0217617

13. EPHPP. Quality assessment tool for quantitative studies Hamilton: Effective Public Health Practice Project 2010 [Available from: https://merst.ca/ephpp/.

14. Armijo-Olivo S, Stiles CR, Hagen NA, et al. Assessment of study quality for systematic reviews: a comparison of the Cochrane Collaboration Risk of Bias Tool and the Effective Public Health Practice Project Quality Assessment Tool: methodological research. *Journal of Evaluation in Clinical Practice* 2012;18(1):12-18. doi: 10.1111/j.1365-2753.2010.01516.x

15. Walsh D, Downe S. Appraising the quality of qualitative research. *Midwifery* 2006;22(2):108-19. doi: 10.1016/j.midw.2005.05.004 [published Online First: 2005/10/26]

16. Solnes Miltenburg A, Roggeveen Y, Elteren Mv, et al. A protocol for a systematic review of birth preparedness and complication readiness programs. *Systematic Reviews* 2013;2(1):11. doi: 10.1186/2046-4053-2-11

17. Review Manager (RevMan) [Computer program] [program]. Version 5.3.5 version. Copenhagen: The Nordic Cochrane Centre, The Cochrane Collaboration, 2014.

18. Bashir Y, Conlon KC. Step by step guide to do a systematic review and meta-analysis for medical professionals. *Irish Journal of Medical Science (1971 -)* 2018;187(2):447-52. doi: 10.1007/s11845-017-1663-3

19. Whiting P, Savovic J, Higgins JP, et al. ROBIS: A new tool to assess risk of bias in systematic reviews was developed. *Journal of clinical epidemiology* 2016;69:225-34. doi: 10.1016/j.jclinepi.2015.06.005 [published Online First: 2015/06/21]

20. Thomas J, Harden A. Methods for the thematic synthesis of qualitative research in systematic reviews. *BMC Medical Research Methodology* 2008;8(1):45. doi: 10.1186/1471-2288-8-45

For peer review only

Background information											
Reviewer's initials	Number assigned to each article/study (e.g., 001, 002)	First author's surname	Full name of the study or article	Year of publication	State the exclusion criteria for the study, or state "none." *If study meets any of the exclusion criteria, end data extraction.*	Does this study meet all inclusion criteria? Check PICOTS, language, year, and inclusion of quality. If all criteria are met, state “all.”	Type of publication	If PubTyp = 5, give details.	Detail any other publications linked to the study (e.g. papers describing methods, additional analyses)	Language of publication
**Reviewer**	**Study ID**	**Author**	**Study Name**	**Publication Date**	**Exclusion Criteria**	**Inclusion Criteria**	**Publication Type$1**	**Publication Type$2**	**Other Publications**	**Language of Publication**

For peer review only

				Intervention background information									
List all countries in which the study was conducted	Country(ies) income group	World region(s)	State the primary or main objective(s)/aim(s) of the study	Give (Month/Year) or (Year)	Give (Month/Year) or (Year)	Intervention duration in months	Which agency or agencies implemented the intervention?	Details	At which geographical level did the study occur?	Give specific detail for previous response (e.g. Bongo district (17 villages, 6,158 inhabitants))	What was the main identifying characteristic of the population targeted?	Give specific detail for previous response (e.g., women in the third trimester). Use author's words (with quotation marks)	Provide the number enrolled in the intervention population
**Country**	**Country Income Group**	**World Region**	**Study Objective**	**Intervention Start Date**	**Intervention End Date**	**Intervention Duration**	**Implementing Agency$1**	**Implementing Agency$2**	**Geographic Level$1**	**Geographic Level$2**	**Study Population$1**	**Study Population$2**	**Study Population$3**

For peer review only - http://bmjopen.bmj.com/site/about/guidelines.xhtml

Provide the number enrolled in the control population<br>**Study Population$4**	Provide the mean age of the intervention population<br>**Study Population$5**	Provide the mean age of the control population<br>**Study Population$6**	Provide the mean age of the combined population (intervention and control)<br>**Study Population$7**	Copy all text describing the intervention; ensure comprehensive extraction of intervention characteristics<br>**Intervention Description**	Did the study examine a single intervention or multiple interventions?<br>**Intervention Quantity**	Was the intervention focused on supply-side or demand-side factors?<br>**Intervention Supply--Demand**	Were women during pregnancy, childbirth and/or postpartum intervention recipients? (Unless it is explicit, it is a no).<br>**Intervention Recipient$1**	Was a subgroup of women during pregnancy, childbirth, postpartum (e.g., women with HIV) the intervention recipient? (Unless it is explicit, it is a no).<br>**Intervention Recipient$2**	Were women defined more generally (e.g., all women, all women of reproductive age) intervention recipients? (Unless it is explicit, it is a no).<br>**Intervention Recipient$3**	Were newborns intervention recipients? (Unless it is explicit, it is a no).<br>**Intervention Recipient$4**	Were children intervention recipients? (Unless it is explicit, it is a no).<br>**Intervention Recipient$5**	Were health care providers intervention recipients? (Unless it is explicit, it is a no).<br>**Intervention Recipient$6**

For peer review only

For peer review only - http://bmjopen.bmj.com/site/about/guidelines.xhtml

Intervention details										
Were any others intervention recipients? (Unless it is explicit, it is a no).	Give specific detail for previous response (Intervention recipient code: "author's description")	Was on-site support for quality improvement introduced/changed as part of the intervention? (Unless it is explicit, it is a no).	Were data systems introduced/changed as part of the intervention? (Unless it is explicit, it is a no).	Were learning systems introduced/changed as part of the intervention? (Unless it is explicit, it is a no).	Was program management selected/changed as part of the intervention? (Unless it is explicit, it is a no).	Was advocacy introduced/ provided as part of the intervention? (Unless it is explicit, it is a no).	Was policy and strategy development introduced/provided as part of the intervention? (Unless it is explicit, it is a no).	Were other systems introduced or changed as part of the intervention?	Give specific details for previous responses (e.g., coaching, training, electronic data systems, community involvement) (Nature of intervention code: "author's description")	Did the text explicitly use one of the following quality terms: 'quality of care,' 'quality care,' 'quality assessment,' 'quality improvement,' 'clinical quality,' 'perceived quality,' or another 'quality' term?
**Intervention Recipient$7**	**Intervention Recipient$8**	**Nature of Intervention$1**	**Nature of Intervention$2**	**Nature of Intervention$3**	**Nature of Intervention$4**	**Nature of Intervention$5**	**Nature of Intervention$6**	**Nature of Intervention$7**	**Nature of Intervention$8**	**Reference to Quality$1**

For peer review only - http://bmjopen.bmj.com/site/about/guidelines.xhtml

Did the text explicitly define 'quality of care,' 'quality care,' 'quality assessment,' 'quality improvement' or another 'quality' term?<br>**Reference to Quality$2**	If Quality$2 = 1, how did the text define/refer to 'quality of care,' 'quality care,' 'quality assessment,' 'quality improvement' or another 'quality' term?' Give exact text in quotation marks.<br>**Reference to Quality$3**	Which of the six dimensions of quality did the study address?<br>**Reference to Quality$4**	If Quality$4 = 7, specify the dimensions (in alphabetical order).<br>**Reference to Quality$5**	Did the study report on the outcome 'maternal morbidity?'<br>**Critical Outcome$1A**	In the author's words, include the text describing the outcome 'maternal morbidity.'<br>**Critical Outcome$1B**	Report all outcome data for 'maternal morbidity.'<br>**Critical Outcome$1C**	Did the study report on the outcome 'maternal mortality?'<br>**Critical Outcome$2A**	In the author's words, include the text describing the outcome 'maternal mortality.'<br>**Critical Outcome$2B**	Report all outcome data for 'maternal mortality.'<br>**Critical Outcome$2C**	Did the study report on the outcome 'newborn morbidity?'<br>**Critical Outcome$3A**	In the author's words, include the text describing the outcome 'newborn morbidity.'<br>**Critical Outcome$3B**	Report all outcome data for 'newborn morbidity.'<br>**Critical Outcome$3C**

For peer review only

Outcomes													
Did the study report on the outcome 'newborn mortality?' **Critical Outcome$4A**	In the author's words, include the text describing the outcome 'newborn mortality.' **Critical Outcome$4B**	Report all outcome data for 'newborn mortality.' **Critical Outcome$4C**	Did the study report on the outcome 'child morbidity?' **Critical Outcome$5A**	In the author's words, include the text describing the outcome 'child morbidity.' **Critical Outcome$5B**	Report all outcome data for 'child morbidity.' **Critical Outcome$5C**	Did the study report on the outcome 'child mortality?' **Critical Outcome$6A**	In the author's words, include the text describing the outcome 'child mortality.' **Critical Outcome$6B**	Report all outcome data for 'child mortality.' **Critical Outcome$6C**	Did the study report on the outcome 'quality of care?' **Critical Outcome$7A**	In the author's words, include the text describing the outcome 'quality of care.' **Critical Outcome$7B**	Report all outcome data for 'quality of care.' **Critical Outcome$7C**	Did the study report on the outcome 'experience of care, including respectful care?' **Critical Outcome$8A**	In the author's words, include the text describing the outcome 'experience of care, including respectful care.' **Critical Outcome$8B**

For peer review only

Report all outcome data for 'experience of care, including respectful care.' **Critical Outcome$8C**	Did the study report on the outcome 'service utilization and efficiency?' **Critical Outcome$9A**	In the author's words, include the text describing the outcome 'service utilization and efficiency.' **Critical Outcome$9B**	Report all outcome data for 'service utilization and efficiency.' **Critical Outcome$9C**	Did the study report on the outcome 'infant and/or child growth?' **Secondary Outcome$1A**	In the author's words, include the text describing the outcome 'infant and/or child growth.' **Secondary Outcome$1B**	Report all outcome data for 'infant and/or child growth.' **Secondary Outcome$1C**	What other outcomes were described or measured? Give details. Include all outcomes relating to stillbirth, childbirth, health system outcomes, and women's views and satisfaction. **Additional Outcomes**	Was the intervention evaluated? **Evaluated**	What type of evaluation was done? **Type of Evaluation$1**	Give details with quotations **Type of Evaluation$2**	What was the design of the study? **Study Type$1**	Give details with quotations **Study Type$2**

For peer review only

Evaluation/study details											
Control or comparator group included?	If Control/ Comparator$1 = 1, give details	What type of data were presented in the evaluation findings/results?	Were the data longitudinal?	What claims are made for the effects of the intervention on the outcome/s relevant to quality of maternal, newborn, and child health care services?	Give details. Use author's words (with quotation marks)	Copy any text about barriers/enablers to the intervention, factors influencing its delivery.	Extract all data on the cost of the intervention and cost compared to outcomes.	Extract all data on the cost effectiveness of the intervention.	Did the study discuss or propose any mechanisms for engaging the private sector in planning, delivering, and demonstrating accountability for quality maternal, newborn, and child health services? If so, give details and use the author's words (with quotation marks).	In what context was the study conducted? Give details (e.g. regulatory environment, collaborative service delivery models).
**Control/ Comparator$1**	**Control/ Comparator$2**	**Data Type**	**Longitudinal Data**	**Intervention Claim$1**	**Intervention Claim$2**	**Strategy Effectiveness**	**Cost Data**	**Cost Effectiveness Data**	**Mechanisms**	**Context**

For peer review only

													Qu
Include any notes you think are relevant<br>**Notes**	Are the individuals selected to participate in the study likely to be representative of the target population?<br>**Selection Bias$1**	What percentage of selected individuals agreed to participate?<br>**Selection Bias$2**	Rate this section (see dictionary)<br>**Selection Bias$3**	Was the study described as randomized? (Check Study Type$1) If NO, go to Confounders $1.<br>**Study Design$1**	If YES, was the method of randomization described? (see dictionary)<br>**Study Design$2**	If YES, was the method appropriate? (see dictionary)<br>**Study Design$3**	Rate this section (see dictionary)<br>**Study Design$4**	Were there important differences between groups prior to the intervention?<br>**Confounders$1**	If YES, indicate the percentage of relevant confounders that were controlled (either in the design (e.g. stratification, matching) or analysis)?<br>**Confounders$2**	Rate this section (see dictionary)<br>**Confounders$3**	Was (were) the outcome assessor(s) aware of the information or exposure status of participants?<br>**Blinding$1**	Were the study participants aware of the research question?<br>**Blinding$2**	Rate this section (see dictionary)<br>**Blinding$3**

For peer review only

uality assessment (for quantitative studies): Reviewer 1

Were data collection tools shown to be valid? **Data Collection Methods$1**	Were data collection tools shown to be reliable? **Data Collection Methods$2**	Rate this section (see dictionary) **Data Collection Methods$3**	Were withdrawals and dropouts reported in terms of numbers and/or reasons per group? **Withdrawals and Dropouts$1**	Indicate the percentage of participants completing the study. (If the percentage differs by groups, record the lowest.) **Withdrawals and Dropouts$2**	Rate this section (see dictionary) **Withdrawals and Dropouts$3**	What percentage of participants received the allocated intervention or exposure of interest? **Intervention Integrity$1**	Was the consistency of the intervention measured? **Intervention Integrity$2**	Is it likely that the subjects received an unintended intervention (contamination or co-intervention) that may influence the results? **Intervention Integrity$3**	Indicate the unit of allocation. **Analyses$1**	Indicate the unit of analysis. **Analyses$2**	Are the statistical methods appropriate for the study design? **Analyses$3**	Is the analysis performed by intervention allocation status (i.e. intention to treat) rather than the actual intervention received? **Analyses$4**	Based on the final section ratings for selection bias, study design, confounders, blinding, data collection methods, and withdrawals and dropouts, what is the global rating for this paper? **Global Rating$1**

For peer review only

With both reviewers discussing the ratings, is there a discrepancy between the two reviewers with respect to the following sections: for selection bias, study design, confounders, blinding, data collection methods, and withdrawals and dropouts **Global Rating$2**	If YES, indicate the reason for the discrepancy. **Global Rating$3**	Final decision of both reviewers **Global Rating$4**	Are the individuals selected to participate in the study likely to be representative of the target population? **Selection Bias$4**	What percentage of selected individuals agreed to participate? **Selection Bias$5**	Rate this section (see dictionary) **Selection Bias$6**	Was the study described as randomized? (Check Study Type$1) If NO, go to Confounders $4. **Study Design$5**	If YES, was the method of randomization described? (see dictionary) **Study Design$6**	If YES, was the method appropriate? (see dictionary) **Study Design$7**	Rate this section (see dictionary) **Study Design$8**	Were there important differences between groups prior to the intervention? **Confounders$4**	If YES, indicate the percentage of relevant confounders that were controlled (either in the design (e.g. stratification, matching) or analysis)? **Confounders$5**	Rate this section (see dictionary) **Confounders$6**

For peer review only

Quality assessment (for quantitative studies): Reviewer 2

Was (were) the outcome assessor(s) aware of the information or exposure status of participants? **Blinding$4**	Were the study participants aware of the research question? **Blinding$5**	Rate this section (see dictionary) **Blinding$6**	Were data collection tools shown to be valid? **Data Collection Methods$4**	Were data collection tools shown to be reliable? **Data Collection Methods$5**	Rate this section (see dictionary) **Data Collection Methods$6**	Were withdrawals and dropouts reported in terms of numbers and/or reasons per group? **Withdrawals and Dropouts$4**	Indicate the percentage of participants completing the study. (If the percentage differs by groups, record the lowest.) **Withdrawals and Dropouts$5**	Rate this section (see dictionary) **Withdrawals and Dropouts$6**	What percentage of participants received the allocated intervention or exposure of interest? **Intervention Integrity$4**	Was the consistency of the intervention measured? **Intervention Integrity$5**	Is it likely that the subjects received an unintended intervention (contamination or co-intervention) that may influence the results? **Intervention Integrity$6**	Indicate the unit of allocation. **Analyses$5**	Indicate the unit of analysis. **Analyses$6**

For peer review only

Are the statistical methods appropriate for the study design?	Is the analysis performed by intervention allocation status (i.e. intention to treat) rather than the actual intervention received?	Based on the final section ratings for selection bias, study design, confounders, blinding, data collection methods, and withdrawals and dropouts, what is the global rating for this paper?	Does the study have a clear statement of, and rationale for, its research question/aims/ purposes?	Is the study thoroughly contextualized by existing literature?	Rate this section	Is the method/design apparent and consistent with the research intent?	Is the data collection strategy apparent and appropriate?	Rate this section	Are the sampling and sampling method appropriate?	Rate this section	Is the analytical approach appropriate?	Rate this section
**Analyses$7**	**Analyses$8**	**Global Rating$5**	**Scope and Purpose$1**	**Scope and Purpose$2**	**Scope and Purpose$3**	**Design$1**	**Design$2**	**Design$3**	**Sampling Strategy$1**	**Sampling Strategy$2**	**Analysis$1**	**Analysis$2**

For peer review only

Quality assessment (for qualitative studies): Reviewer 1										
Is the context described and taken account of in the interpretation?	Is a clear audit trail given?	Are data used to support interpretation?	Rate this section	Is researcher reflexivity demonstrated?	Rate this section	Is there demonstration of sensitivity to ethical concerns?	Rate this section	Are relevance and transferability evident?	Rate this section	Based on the final section ratings for scope and purpose, design, sampling strategy, analysis, interpretation, reflexivity, ethical dimensions, and relevance and transferability, what is the global rating for this paper?
**Interpretation$1**	**Interpretation$2**	**Interpretation$3**	**Interpretation$4**	**Reflexivity$1**	**Reflexivity$2**	**Ethical Dimensions$1**	**Ethical Dimensions$2**	**Relevance and Transferability$1**	**Relevance and Transferability$2**	**Global Rating$1**

For peer review only

			Quality									
With both reviewers discussing the ratings, is there a discrepancy between the two reviewers with respect to the following sections: scope and purpose, design, sampling strategy, analysis, interpretation, reflexivity, ethical dimensions, and relevance and transferability	If YES, indicate the reason for the discrepancy.	Final decision of both reviewers	Does the study have a clear statement of, and rationale for, its research question/aims/ purposes?	Is the study thoroughly contextualized by existing literature?	Rate this section	Is the method/design apparent and consistent with the research intent?	Is the data collection strategy apparent and appropriate?	Rate this section	Are the sampling and sampling method appropriate?	Rate this section	Is the analytical approach appropriate?	Rate this section
**Global Rating$2**	**Global Rating$3**	**Global Rating$4**	**Scope and Purpose$4**	**Scope and Purpose$5**	**Scope and Purpose$6**	**Design$4**	**Design$5**	**Design$6**	**Sampling Strategy$3**	**Sampling Strategy$4**	**Analysis$3**	**Analysis$4**

For peer review only

assessment (for qualitative studies): Reviewer 2

Is the context described and taken account of in the interpretation?	Is a clear audit trail given?	Are data used to support interpretation?	Rate this section	Is researcher reflexivity demonstrated?	Rate this section	Is there demonstration of sensitivity to ethical concerns?	Rate this section	Are relevance and transferability evident?	Rate this section	Based on the final section ratings for scope and purpose, design, sampling strategy, analysis, interpretation, reflexivity, ethical dimensions, and relevance and transferability, what is the global rating for this paper?
**Interpretation$5**	**Interpretation$6**	**Interpretation$7**	**Interpretation$8**	**Reflexivity$3**	**Reflexivity$4**	**Ethical Dimensions$3**	**Ethical Dimensions$4**	**Relevance and Transferability$3**	**Relevance and Transferability$4**	**Global Rating$5**

For peer review only

Annex B: Codebook

Variable	Question and instruction	Response options
Background information		
Reviewer	State your initials	SL<br>BM
Study ID	Number assigned to each article/study (e.g., 001, 002)	#
Author	First author's surname	Text
Study Name	Full name of the study or article	Text
Publication Date	Year of publication	#
Exclusion Criteria	State the exclusion criteria for the study, or state "none."<br><br>*If study meets any of the exclusion criteria, end data extraction.*	Text
Inclusion Criteria	Does this study meet all inclusion criteria? Check PICOTS, language, year, and inclusion of quality. If all criteria are met, state "all."<br><br>*If all of the inclusion criteria are not met, return to "exclusion criteria."*	All
Publication Type$1	Type of publication	1 = Peer-reviewed journal article<br>2 = Non-peer-reviewed journal article<br>3 = Report<br>4 = Book or book chapter<br>5 = Other
Publication Type$2	If PubTyp = 5, give details.	Text<br>99 = Not applicable
Other Publications	Detail any other publications linked to the study (e.g. papers describing methods, additional analyses)	Text
Language of Publication		1 = English<br>2 = French<br>3 = German<br>4 = Italian<br>5 = Other
Country	List all countries in which the study was conducted	Text
Country Income Group[1]	Country income group	1 = Low<br>2 = Lower-middle<br>3 = Upper-middle<br>4 = Multiple
World Region[2]	World region(s)	1 = Africa

[1] World Bank Atlas method groupings of countries by income: https://datahelpdesk.worldbank.org/knowledgebase/articles/906519
[2] WHO region groupings: Africa, Asia, Europe, Latin America and the Caribbean, Northern America, Oceania: https://unstats.un.org/unsd/methodology/m49/

For peer review only - http://bmjopen.bmj.com/site/about/guidelines.xhtml

Variable	Question and instruction	Response options
		2 = Asia<br>3 = Europe<br>4 = Latin America and the Caribbean<br>5 = Northern America<br>6 = Oceania<br>7 = Multiple
Study Objective	State the primary or main objective(s) or aim(s) of the study	Text
Intervention background information		
Intervention Start Date	Give (Month/Year) or (Year)<br><br>[Give start date of implementation rather than preliminary work]	(MM/YYYY) or (YYYY)<br>98 = Unclear/not specified
Intervention End Date	Give (Month/Year) or (Year)	(MM/YYYY) or (YYYY)<br>Ongoing= Ongoing at time of publication<br>98= Unclear/not specified
Intervention Duration	Duration of the intervention in months. If not straightforward, give details.	Text<br>98 = Unclear/not specified
Implementing Agency$1	Which type of agency or agencies implemented the intervention?	1 = Domestic government (in the case of a public-private partnership)<br>2 = Domestic non-profit organization (e.g., charity, civil society organization)<br>3 = Domestic for-profit organization<br>4 = International non-governmental organization (NGO)<br>5 = International for-profit organization<br>6 = Other international institution<br>7 = Multiple answers from list<br>8 = Other<br>98 = Unclear/not specified
Implementing Agency$2	If Agency$1 = 1-8, give details. (e.g., international NGO “Health Unlimited”…)	Text<br>99 = Not applicable
Geographic Level$1	At which geographical level did the study occur?	1 = National<br>2 = Sub-national (e.g., region, state, county, district, whole city)<br>3 = Local (e.g., village, neighborhood)<br>4 = Health facility<br>5 = Other<br>98 = Unclear/not specified
Geographic Level$2	Give specific detail for previous response (e.g. Bongo district (17 villages, 6,158 inhabitants)). Provide name if only one location unit.	Text<br>99 = if GeoLvl$1 = 98

For peer review only

For peer review only - http://bmjopen.bmj.com/site/about/guidelines.xhtml

Variable	Question and instruction	Response options
Study Population$1	What was the main identifying characteristic of the population the study targeted?	1 = Pregnant women<br>2 = Women during childbirth<br>3 = Mothers postpartum<br>4 = Newborns<br>5 = Children<br>6 = Health care providers<br>7 = Multiple answers from list<br>8 = Other, specify<br>98 = Unclear/not specified<br>99 = Not applicable
Study Population$2	Give specific detail for previous response (e.g., women in the third trimester). Use author's words (with quotation marks).	Text
Study Population$3	Provide the number enrolled in the intervention population.	#<br>98 = Unclear/not specified
Study Population$4	Provide the number enrolled in the control population, if applicable.	#<br>98 = Unclear/not specified<br>99 = No control population
Study Population$5	Provide the mean age of the intervention population.	#<br>98 = Unclear/not specified
Study Population$6	Provide the mean age of the control population, if applicable.	#<br>98 = Unclear/not specified<br>99 = No control population
Study Population$7	Provide the mean age of the combined population (intervention and control), if applicable.	#<br>98 = Unclear/not specified<br>99 = No control population
Intervention details		
Intervention Description	Copy all text describing the intervention; ensure comprehensive extraction of intervention characteristics.	Text
Intervention Quantity	Did the study examine a single intervention or multiple interventions?	1 = Single intervention<br>2 = Multiple interventions<br>98 = Unclear/not specified
Intervention Supply – Demand	Was the intervention focused on supply-side or demand-side factors?	1 = Supply side (e.g., staff training, change in types of staff, service environment, provider communication skills)<br>2 = Demand-side (e.g., education, peer encouragement for service use)<br>3 = Both<br>98 = Unclear/not specified
Intervention Recipient$1	Were women during pregnancy, childbirth and/or postpartum intervention recipients?<br>(Unless it is explicit, it is a no).	1 = Indirect<br>2 = Direct<br>3 = Not a recipient
Intervention Recipient$2	Was a subgroup of women during pregnancy, childbirth, postpartum	1 = Indirect<br>2 = Direct

For peer review only - http://bmjopen.bmj.com/site/about/guidelines.xhtml

Variable	Question and instruction	Response options
	(e.g., women who experience birth complications, women with HIV) the intervention recipient? (Unless it is explicit, it is a no).	3 = Not a recipient
Intervention Recipient$3	Were women defined more generally (e.g., all women, all women of reproductive age) intervention recipients? (Unless it is explicit, it is a no).	1 = Indirect<br>2 = Direct<br>3 = Not a recipient
Intervention Recipient$4	Were newborns intervention recipients? (Unless it is explicit, it is a no).	1 = Indirect<br>2 = Direct<br>3 = Not a recipient
Intervention Recipient$5	Were children intervention recipients? (Unless it is explicit, it is a no).	1 = Indirect<br>2 = Direct<br>3 = Not a recipient
Intervention Recipient$6	Were health care providers intervention recipients? (Unless it is explicit, it is a no).	1 = Indirect<br>2 = Direct<br>3 = Not a recipient
Intervention Recipient$7	Were any others intervention recipients? (Unless it is explicit, it is a no).	1 = Indirect<br>2 = Direct<br>3 = Not a recipient
Intervention Recipient$8	Give specific detail for previous response (Intervention recipient code: "author's description")	Text<br>99 = If Recipient$1-Recipient$7 = 3
Nature of Intervention$1	Was on-site support for quality improvement introduced/changed as part of the intervention? (Unless it is explicit, it is a no).	0 = No<br>1 = Yes
Nature of Intervention$2	Were data systems introduced/changed as part of the intervention? (Unless it is explicit, it is a no).	0 = No<br>1 = Yes
Nature of Intervention$3	Were learning systems introduced/changed as part of the intervention? (Unless it is explicit, it is a no).	0 = No<br>1 = Yes
Nature of Intervention$4	Was a program management selected/changed as part of the intervention? (Unless it is explicit, it is a no).	0 = No<br>1 = Yes
Nature of Intervention$5	Was advocacy introduced/ provided as part of the intervention? (Unless it is explicit, it is a no).	0 = No<br>1 = Yes
Nature of Intervention$6	Was policy and strategy development introduced/provided as part of the intervention? (Unless it is explicit, it is a no).	0 = No<br>1 = Yes
Nature of	Were other systems introduced or	0 = No

For peer review only - http://bmjopen.bmj.com/site/about/guidelines.xhtml

Variable	Question and instruction	Response options
Intervention$7	changed as part of the intervention?	1 = Yes
Nature of Intervention$8	Give specific details for previous responses (e.g., coaching, training, electronic data systems, community involvement)<br>(Nature of intervention code: "author's description")	Text
Reference to Quality$1	Did the text explicitly use one of the following quality terms: 'quality of care,' 'quality care,' 'quality assessment,' 'quality improvement,' 'clinical quality,' 'perceived quality,' or another 'quality' term?	0 = No<br>1 = Yes, quality of care or quality care<br>2 = Yes, quality assessment<br>3 = Yes, quality improvement<br>4 = Yes, clinical quality<br>5 = Yes, perceived quality<br>6 = Yes, multiple quality terms<br>7 = Yes, another quality term [specify in Quality$3]
Reference to Quality$2	Did the text explicitly define 'quality of care,' 'quality care,' 'quality assessment,' 'quality improvement,' 'clinical quality,' 'perceived quality,' or another 'quality' term?	0 = No<br>1 = Yes
Reference to Quality$3	If Quality$2 = 1, how did the text define/refer to 'quality of care,' 'quality care,' 'quality assessment,' 'quality improvement,' 'clinical quality,' 'perceived quality,' or another 'quality' term? Give exact text in quotation marks.	Text<br>99 = If Quality$2=0
Reference to Quality$4	Which of the six dimensions of quality[3] did the study address?<br>• *Safe*—delivering health care which minimises risks and harm to service users, including avoiding preventable injuries and reducing medical errors<br>• *Effective*—providing services based on scientific knowledge and evidence-based guidelines<br>• *Timely*—reducing delays in providing/receiving health care<br>• *Efficient*—delivering health care in a manner which maximises	1 = Safe<br>2 = Effective<br>3 = Timely<br>4 = Efficient<br>5 = Equitable<br>6 = People-centered<br>7 = Multiple (2-5) dimensions<br>8 = All dimensions<br>98 = Unclear/not specified

[3] Operational definitions for the characteristics of quality care come from WHO's vision for quality of care for pregnant women and newborns: https://www.ncbi.nlm.nih.gov/pmc/articles/PMC5029576/

For peer review only - http://bmjopen.bmj.com/site/about/guidelines.xhtml

Variable	Question and instruction	Response options
	resource use and avoids wastage<br>• *Equitable*—delivering health care which does not vary in quality because of personal characteristics such as gender, race, ethnicity, geographical location or socioeconomic status<br>• *People-centered*—providing care which takes into account the preferences and aspirations of individual service users and the cultures of their communities	
Reference to Quality$5	If Quality$4 = 7, specify the dimensions (in alphabetical order).	Text
Outcomes		
Critical Outcome$1A	Did the study report on the outcome 'maternal morbidity?'	0 = No<br>1 = Yes<br>98 = Unclear/not specified
Critical Outcome$1B	In the author's words, include the text describing the outcome 'maternal morbidity.'	Text<br>98 = Unclear/not specified<br>99 = If Outcom$1A=0
Critical Outcome$1C	Report all outcome data for 'maternal morbidity.'	Text<br>98 = Unclear/not specified<br>99 = If Outcom$1A=0
Critical Outcome$2A	Did the study report on the outcome 'maternal mortality?'	0 = No<br>1 = Yes<br>98 = Unclear/not specified
Critical Outcome$2B	In the author's words, include the text describing the outcome 'maternal mortality.'	Text<br>98 = Unclear/not specified<br>99 = If Outcom$2A=0
Critical Outcome$2C	Report all outcome data for 'maternal mortality.'	Text<br>98 = Unclear/not specified<br>99 = If Outcom$2A=0
Critical Outcome$3A	Did the study report on the outcome 'newborn morbidity?'	0 = No<br>1 = Yes<br>98 = Unclear/not specified
Critical Outcome$3B	In the author's words, include the text describing the outcome 'newborn morbidity.'	Text<br>98 = Unclear/not specified<br>99 = If Outcom$3A=0
Critical Outcome$3C	Report all outcome data for 'newborn morbidity.'	Text<br>98 = Unclear/not specified<br>99 = If Outcom$3A=0
Critical Outcome$4A	Did the study report on the outcome 'newborn mortality?'	0 = No<br>1 = Yes<br>98 = Unclear/not specified
Critical Outcome$4B	In the author's words, include the	Text

For peer review only - http://bmjopen.bmj.com/site/about/guidelines.xhtml

Variable	Question and instruction	Response options
	text describing the outcome 'newborn mortality.'	98 = Unclear/not specified<br>99 = If Outcom$4A=0
Critical Outcome$4C	Report all outcome data for 'newborn mortality.'	Text<br>98 = Unclear/not specified<br>99 = If Outcom$4A=0
Critical Outcome$5A	Did the study report on the outcome 'child morbidity?'	0 = No<br>1 = Yes<br>98 = Unclear/not specified
Critical Outcome$5B	In the author's words, include the text describing the outcome 'child morbidity.'	Text<br>98 = Unclear/not specified<br>99 = If Outcom$5A=0
Critical Outcome$5C	Report all outcome data for 'child morbidity.'	Text<br>98 = Unclear/not specified<br>99 = If Outcom$5A=0
Critical Outcome$6A	Did the study report on the outcome 'child mortality?'	0 = No<br>1 = Yes<br>98 = Unclear/not specified
Critical Outcome$6B	In the author's words, include the text describing the outcome 'child mortality.'	Text<br>98 = Unclear/not specified<br>99 = If Outcom$6A=0
Critical Outcome$6C	Report all outcome data for 'child mortality.'	Text<br>98 = Unclear/not specified<br>99 = If Outcom$6A=0
Critical Outcome$7A	Did the study report on the outcome 'quality of care?' This includes outcomes involving:<br>• Safety<br>• Efficacy<br>• Timeliness<br>• Efficiency<br>• Equitability<br>• People-centered care	0 = No<br>1 = Yes<br>98 = Unclear/not specified
Critical Outcome$7B	In the author's words, include the text describing the outcome 'quality of care.'	Text<br>98 = Unclear/not specified<br>99 = If Outcom$7A=0
Critical Outcome$7C	Report all outcome data for 'quality of care.'	Text<br>98 = Unclear/not specified<br>99 = If Outcom$7A=0
Critical Outcome$8A	Did the study report on the outcome 'experience of care, including respectful care?'	0 = No<br>1 = Yes<br>98 = Unclear/not specified
Critical Outcome$8B	In the author's words, include the text describing the outcome 'experience of care, including respectful care.'	Text<br>98 = Unclear/not specified<br>99 = If Outcom$8A=0
Critical Outcome$8C	Report all outcome data for 'experience of care, including respectful care.'	Text<br>98 = Unclear/not specified<br>99 = If Outcom$2A=0
Critical Outcome$9A	Did the study report on the	0 = No

For peer review only - http://bmjopen.bmj.com/site/about/guidelines.xhtml

Variable	Question and instruction	Response options
	outcome 'service utilization and efficiency?'	1 = Yes<br>98 = Unclear/not specified
Critical Outcome$9B	In the author's words, include the text describing the outcome 'service utilization and efficiency.'	Text<br>98 = Unclear/not specified<br>99 = If Outcom$9A=0
Critical Outcome$9C	Report all outcome data for 'service utilization and efficiency.'	Text<br>98 = Unclear/not specified<br>99 = If Outcom$9A=0
Secondary Outcome$1A	Did the study report on the outcome 'infant and/or child growth?'	0 = No<br>1 = Yes<br>98 = Unclear/not specified
Secondary Outcome$1B	In the author's words, include the text describing the outcome 'infant and/or child growth.'	Text<br>98 = Unclear/not specified<br>99 = If Outcom$9A=0
Secondary Outcome$1C	Report all outcome data for 'infant and/or child growth.'	Text<br>98 = Unclear/not specified<br>99 = If Outcom$9A=0
Additional Outcomes	What other outcomes were described or measured? Give details. Include all outcomes relating to stillbirth, childbirth, health system outcomes, and women's views and satisfaction.	Text
Evaluation/study details		
Evaluated	Was the intervention evaluated?	0 = No<br>1 = Yes
Type of Evaluation$1	What type of evaluation was done?	1 = Impact (quantitative change in relevant outcome (use of services))<br>2 = Process (evaluation of intervention implementation)<br>3 = Impact and process<br>4 = Economic<br>5 = Multiple<br>6 = Other<br>98 = Unclear/not specified<br>99 = Not applicable
Type of Evaluation$2	Give details with quotations	Text<br>99 = Not applicable
Study Type$1	What was the design of the study?	1 = Randomized controlled trial<br>2 = Controlled clinical trial<br>3 = Cohort analytic (two groups pre + post)<br>4 = Case-control<br>5 = Cohort (one group pre + post (before and after))<br>6 = Interrupted time series<br>7 = Qualitative<br>8 = Mixed methods<br>9 = Regression analysis

For peer review only - http://bmjopen.bmj.com/site/about/guidelines.xhtml

Variable	Question and instruction	Response options
		10 = Other<br>98 = Unclear/not specified
Study Type$2	Give details with quotations	Text<br>99 = Not applicable
Control/Comparator$1	Control/comparator group included?	0 = No<br>1 = Yes<br>98 = Unclear/not specified
Control/Comparator$2	If Control/Comparator$1 = 1, give details	Text<br>99= Not applicable
Data Type	What type of data were presented in the evaluation findings/results?	1 = Quantitative<br>2 = Qualitative<br>3 = Both
Longitudinal Data	Were the data longitudinal?	0 = No<br>1 = Yes<br>98 = Unclear/not specified
Intervention Claims$1	What claims are made for the effects of the intervention on the outcome/s relevant to quality of maternal, newborn, and child health services?	1 = Positive<br>2 = Neutral<br>3 = Negative<br>4 = Mixed (give details)<br>5 = Other (give details)<br>98 = Unclear/not specified
Intervention Claims$2	Give details. Use author's words (with quotation marks).	Text<br>99 = Not applicable
Strategy Effectiveness	Copy any text about barriers/enablers to the intervention, factors influencing its delivery.	Text
Cost Data	Extract all data on the cost of the intervention and cost compared to outcomes.	Text
Cost Effectiveness Data	Extract all data on the cost effectiveness of the intervention.	Text
Mechanisms	Did the study discuss or propose any mechanisms for engaging the private sector in planning, delivering, and demonstrating accountability for quality maternal, newborn, and child health services? If so, give details and use the author's words (with quotation marks).	Text
Context	In what context was the study conducted? Give details (e.g. regulatory environment, collaborative service delivery models).	Text
Notes	Include any notes you think are relevant	Text

For peer review only

Variable	Question and instruction	Response options
Quality assessment (for quantitative studies): Reviewer 1		
Selection Bias$1	Are the individuals selected to participate in the study likely to be representative of the target population?	1 = Very likely<br>2 = Somewhat likely<br>3 = Not likely<br>98 = Unclear/not specified
Selection Bias$2	What percentage of selected individuals agreed to participate?	1 = 80-100% agreement<br>2 = 60-79% agreement<br>3 = Less than 60% agreement<br>98 = Unclear/not specified<br>99 = Not applicable
Selection Bias$3	Rate this section (see dictionary[4])	1 = Strong<br>2 = Moderate<br>3 = Weak
Study Design$1	Was the study described as randomized? (Check Study Type$1)<br><br>If NO, go to Confounders$1.	0 = No<br>1 = Yes
Study Design$2	If YES, was the method of randomization described? (see dictionary)	0 = No<br>1 = Yes
Study Design$3	If YES, was the method appropriate? (see dictionary)	0 = No<br>1 = Yes
Study Design$4	Rate this section (see dictionary)	1 = Strong<br>2 = Moderate<br>3 = Weak
Confounders$1	Were there important differences between groups prior to the intervention?<br><br>The following are examples of confounders:<br>1. Race<br>2. Sex<br>3. Marital status/family<br>4. Age<br>5. SES (income or class)<br>6. Education<br>7. Health status<br>Pre-intervention score on outcome measure	0 = No<br>1 = Yes<br>98 = Unclear/not specified
Confounders$2	If YES, indicate the percentage of relevant confounders that were controlled (either in the design (e.g. stratification, matching) or analysis)?	1 = 80-100% (most)<br>2 = 60-79% (some)<br>3 = Less than 60% (few or none)<br>98 = Unclear/not specified<br>99 = Confou$1=0 or 98
Confounders$3	Rate this section (see dictionary)	1 = Strong

[4] Follow EPHPP's 'Quality Assessment Tool for Quantitative Studies Dictionary' to score study quality for quantitative studies.

For peer review only - http://bmjopen.bmj.com/site/about/guidelines.xhtml

Variable	Question and instruction	Response options
		2 = Moderate<br>3 = Weak
Blinding$1	Was (were) the outcome assessor(s) aware of the information or exposure status of participants?	0 = No<br>1 = Yes<br>98 = Unclear/not specified
Blinding$2	Were the study participants aware of the research question?	0 = No<br>1 = Yes<br>98 = Unclear/not specified
Blinding$3	Rate this section (see dictionary)	1 = Strong<br>2 = Moderate<br>3 = Weak
Data Collection Methods$1	Were data collection tools shown to be valid?	0 = No<br>1 = Yes<br>98 = Unclear/not specified
Data Collection Methods$2	Were data collection tools shown to be reliable?	0 = No<br>1 = Yes<br>98 = Unclear/not specified
Data Collection Methods$3	Rate this section (see dictionary)	1 = Strong<br>2 = Moderate<br>3 = Weak
Withdrawals and Dropouts$1	Were withdrawals and dropouts reported in terms of numbers and/or reasons per group?	0 = No<br>1 = Yes<br>98 = Unclear/not specified<br>99 = Not applicable
Withdrawals and Dropouts$2	Indicate the percentage of participants completing the study. (If the percentage differs by groups, record the lowest.)	1 = 80-100%<br>2 = 60-79%<br>3 = Less than 60%<br>98 = Unclear/not specified<br>99 = Not applicable
Withdrawals and Dropouts$3	Rate this section (see dictionary)	1 = Strong<br>2 = Moderate<br>3 = Weak<br>99 = Not applicable
Intervention Integrity$1	What percentage of participants received the allocated intervention or exposure of interest?	1 = 80-100%<br>2 = 60-79%<br>3 = Less than 60%<br>98 = Unclear/not specified
Intervention Integrity$2	Was the consistency of the intervention measured?	0 = No<br>1 = Yes<br>98 = Unclear/not specified
Intervention Integrity$3	Is it likely that the subjects received an unintended intervention (contamination or co-intervention) that may influence the results?	0 = No<br>1 = Yes<br>98 = Unclear/not specified
Analyses$1	Indicate the unit of allocation.	1 = Community<br>2 = Organization/institution<br>3 = Practice/office

For peer review only - http://bmjopen.bmj.com/site/about/guidelines.xhtml

Variable	Question and instruction	Response options
		4 = Individual
Analyses$2	Indicate the unit of analysis.	1 = Community<br>2 = Organization/institution<br>3 = Practice/office<br>4 = Individual
Analyses$3	Are the statistical methods appropriate for the study design?	0 = No<br>1 = Yes<br>98 = Unclear/not specified
Analyses$4	Is the analysis performed by intervention allocation status (i.e. intention to treat) rather than the actual intervention received?	0 = No<br>1 = Yes<br>98 = Unclear/not specified
Global Rating$1	Based on the final section ratings for selection bias, study design, confounders, blinding, data collection methods, and withdrawals and dropouts, what is the global rating for this paper?	1 = Strong (no WEAK ratings)<br>2 = Moderate (one WEAK rating)<br>3 = Weak (two or more WEAK ratings)
Global Rating$2	With both reviewers discussing the ratings, is there a discrepancy between the two reviewers with respect to the following sections: for selection bias, study design, confounders, blinding, data collection methods, and withdrawals and dropouts	0 = No<br>1 = Yes
Global Rating$3	If YES, indicate the reason for the discrepancy.	1 = Oversight<br>2 = Differences in interpretation of criteria<br>3 = Differences in interpretation of study
Global Rating$4	Final decision of both reviewers	1 = Strong<br>2 = Moderate<br>3 = Weak
Quality assessment (for quantitative studies): Reviewer 2		
Selection Bias$4	Are the individuals selected to participate in the study likely to be representative of the target population?	1 = Very likely<br>2 = Somewhat likely<br>3 = Not likely<br>98 = Unclear/not specified
Selection Bias$5	What percentage of selected individuals agreed to participate?	1 = 80-100% agreement<br>2 = 60-79% agreement<br>3 = Less than 60% agreement<br>98 = Unclear/not specified<br>99 = Not applicable
Selection Bias$6	Rate this section (see dictionary[5])	1 = Strong<br>2 = Moderate

[5] Follow EPHPP's 'Quality Assessment Tool for Quantitative Studies Dictionary' to score study quality for quantitative studies.

For peer review only - http://bmjopen.bmj.com/site/about/guidelines.xhtml

Variable	Question and instruction	Response options
		3 = Weak
Study Design$5	Was the study described as randomized? (Check Study Type$1)<br><br>If NO, go to Confounders$4.	0 = No<br>1 = Yes
Study Design$6	If YES, was the method of randomization described? (see dictionary)	0 = No<br>1 = Yes
Study Design$7	If YES, was the method appropriate? (see dictionary)	0 = No<br>1 = Yes
Study Design$8	Rate this section (see dictionary)	1 = Strong<br>2 = Moderate<br>3 = Weak
Confounders$4	Were there important differences between groups prior to the intervention?<br><br>The following are examples of confounders:<br>8. Race<br>9. Sex<br>10. Marital status/family<br>11. Age<br>12. SES (income or class)<br>13. Education<br>14. Health status<br>Pre-intervention score on outcome measure	0 = No<br>1 = Yes<br>98 = Unclear/not specified
Confounders$5	If YES, indicate the percentage of relevant confounders that were controlled (either in the design (e.g. stratification, matching) or analysis)?	1 = 80-100% (most)<br>2 = 60-79% (some)<br>3 = Less than 60% (few or none)<br>98 = Unclear/not specified<br>99 = Confou$1=0 or 98
Confounders$6	Rate this section (see dictionary)	1 = Strong<br>2 = Moderate<br>3 = Weak
Blinding$4	Was (were) the outcome assessor(s) aware of the information or exposure status of participants?	0 = No<br>1 = Yes<br>98 = Unclear/not specified
Blinding$5	Were the study participants aware of the research question?	0 = No<br>1 = Yes<br>98 = Unclear/not specified
Blinding$6	Rate this section (see dictionary)	1 = Strong<br>2 = Moderate<br>3 = Weak
Data Collection Methods$4	Were data collection tools shown to be valid?	0 = No<br>1 = Yes<br>98 = Unclear/not specified

For peer review only

Variable	Question and instruction	Response options
Data Collection Methods$5	Were data collection tools shown to be reliable?	0 = No<br>1 = Yes<br>98 = Unclear/not specified
Data Collection Methods$6	Rate this section (see dictionary)	1 = Strong<br>2 = Moderate<br>3 = Weak
Withdrawals and Dropouts$4	Were withdrawals and dropouts reported in terms of numbers and/or reasons per group?	0 = No<br>1 = Yes<br>98 = Unclear/not specified<br>99 = Not applicable
Withdrawals and Dropouts$5	Indicate the percentage of participants completing the study. (If the percentage differs by groups, record the lowest.)	1 = 80-100%<br>2 = 60-79%<br>3 = Less than 60%<br>98 = Unclear/not specified<br>99 = Not applicable
Withdrawals and Dropouts$6	Rate this section (see dictionary)	1 = Strong<br>2 = Moderate<br>3 = Weak<br>99 = Not applicable
Intervention Integrity$4	What percentage of participants received the allocated intervention or exposure of interest?	1 = 80-100%<br>2 = 60-79%<br>3 = Less than 60%<br>98 = Unclear/not specified
Intervention Integrity$5	Was the consistency of the intervention measured?	0 = No<br>1 = Yes<br>98 = Unclear/not specified
Intervention Integrity$6	Is it likely that the subjects received an unintended intervention (contamination or co-intervention) that may influence the results?	0 = No<br>1 = Yes<br>98 = Unclear/not specified
Analyses$5	Indicate the unit of allocation.	1 = Community<br>2 = Organization/institution<br>3 = Practice/office<br>4 = Individual
Analyses$6	Indicate the unit of analysis.	1 = Community<br>2 = Organization/institution<br>3 = Practice/office<br>4 = Individual
Analyses$7	Are the statistical methods appropriate for the study design?	0 = No<br>1 = Yes<br>98 = Unclear/not specified
Analyses$8	Is the analysis performed by intervention allocation status (i.e. intention to treat) rather than the actual intervention received?	0 = No<br>1 = Yes<br>98 = Unclear/not specified
Global Rating$5	Based on the final section ratings for selection bias, study design, confounders, blinding, data	1 = Strong (no WEAK ratings)<br>2 = Moderate (one WEAK rating)<br>3 = Weak (two or more WEAK ratings)

For peer review only - http://bmjopen.bmj.com/site/about/guidelines.xhtml

Variable	Question and instruction	Response options
	collection methods, and withdrawals and dropouts, what is the global rating for this paper?	
Quality assessment (for qualitative studies): Reviewer 1		
Scope and Purpose$1	Does the study have a clear statement of, and rationale for, its research question/aims/purposes?<br><br>Specific prompts:<br>▯ Clarity of focus demonstrated<br>▯ Explicit purpose given, such as descriptive/explanatory intent, theory building, hypothesis testing<br>▯ Link between research and existing knowledge demonstrated	0 = No<br>1 = Yes
Scope and Purpose$2	Is the study thoroughly contextualized by existing literature?<br><br>Specific prompt:<br>▯ Evidence of systematic approach to literature review, location of literature to contextualize the findings, or both	0 = No<br>1 = Yes
Scope and Purpose$3	Rate this section.<br><br>When Purpose$1 and Purpose$2 = 1, then Purpose$3 = 1. When Purpose$1 or Purpose$2 = 0, then Purpose$3 = 2. When Purpose$1 and Purpose$2 = 0, then Purpose$3 = 3.	1 = Strong<br>2 = Moderate<br>3 = Weak
Design$1	Is the method/design apparent and consistent with the research intent?<br><br>Specific prompts:<br>▯ Rationale given for use of qualitative design<br>▯ Discussion of epistemological/ontological grounding<br>▯ Rationale explored for specific qualitative method (e.g. ethnography, grounded theory, phenomenology)<br>▯ Discussion of why particular method chosen is most appropriate/sensitive/relevant for research question/aims	0 = No<br>1 = Yes

For peer review only - http://bmjopen.bmj.com/site/about/guidelines.xhtml

Variable	Question and instruction	Response options
	▯ Setting appropriate	
Design$2	Is the data collection strategy apparent and appropriate?<br><br>Specific prompts:<br>▯ Were data collection methods appropriate for type of data required and for specific qualitative method?<br>▯ Were they likely to capture the complexity/diversity of experience and illuminate context in sufficient detail?<br>▯▯Was triangulation of data sources used if appropriate?	0 = No<br>1 = Yes
Design$3	Rate this section<br><br>When Design$1 and Design$2 = 1, then Design$3 = 1. When Design$1 or Design$2 = 0, then Design$3 = 2. When Design$1 and Design$2 = 0, then Design$3 = 3.	1 = Strong<br>2 = Moderate<br>3 = Weak
Sampling Strategy$1	Are the sampling and sampling method appropriate?<br><br>Specific prompts:<br>▯ Selection criteria detailed, and description of how sampling was undertaken<br>▯ Justification for sampling strategy given<br>▯ Thickness of description likely to be achieved from sampling<br>▯ Any disparity between planned and actual sample explained	0 = No<br>1 = Yes
Sampling Strategy$2	Rate this section<br><br>When Strategy$1 = 1, then Strategy$2 = 1. When Strategy$1 = 0, then Strategy$2 = 3. At the reviewer's discretion, Strategy$2 may be rated as 2, if the study met most but not all of the prompts.	1 = Strong<br>2 = Moderate<br>3 = Weak
Analysis$1	Is the analytical approach appropriate?<br><br>Specific prompts:<br>▯ Approach made explicit (e.g. Thematic distillation, constant comparative method, grounded	0 = No<br>1 = Yes

For peer review only - http://bmjopen.bmj.com/site/about/guidelines.xhtml

Variable	Question and instruction	Response options
	theory)<br>? Was it appropriate for the qualitative method chosen?<br>? Was data managed by software package or by hand and why?<br>? Discussion of how coding systems/conceptual frameworks evolved<br>? How was context of data retained during analysis<br>? Evidence that the subjective meanings of participants were portrayed<br>? Evidence of more than one researcher involved in stages if appropriate to epistemological/theoretical stance<br>? Did research participants have any involvement in analysis (e.g. member checking)<br>? Evidence provided that data reached saturation or discussion/rationale if it did not<br>? Evidence that deviant data was sought, or discussion/rationale if it was not	
Analysis$2	Rate this section<br><br>When Analysis$1 = 1, then Analysis$2 = 1. When Analysis$1 = 0, then Analysis$2 = 3. At the reviewer's discretion, Analysis$2 may be rated as 2, if the study met most but not all of the prompts.	1 = Strong<br>2 = Moderate<br>3 = Weak
Interpretation$1	Is the context described and taken account of in the interpretation?<br><br>Specific prompts:<br>? Description of social/physical and interpersonal contexts of data collection<br>? Evidence that researcher spent time 'dwelling with the data', interrogating it for competing/alternative explanations of phenomena	0 = No<br>1 = Yes
Interpretation$2	Is a clear audit trail given?<br><br>Specific prompt:<br>? Sufficient discussion of research	0 = No<br>1 = Yes

For peer review only - http://bmjopen.bmj.com/site/about/guidelines.xhtml

Variable	Question and instruction	Response options
	processes such that others can follow the 'decision trail'	
Interpretation$3	Are data used to support interpretation?<br><br>Specific prompts:<br>? Extensive use of field notes entries/verbatim interview quotes in discussion of findings<br>? Clear exposition of how interpretation led to conclusions	0 = No<br>1 = Yes
Interpretation$4	Rate this section<br><br>When Interpretation$1, Interpretation$2, and Interpretation$3 = 1, then Interpretation$4 = 1. When one response to Interpretation$1—Interpretation$3 = 0, then Interpretation$4 = 2. When two or more responses to Interpretation$1—Interpretation$3 = 0, then Interpretation$4 = 3.	1 = Strong<br>2 = Moderate<br>3 = Weak
Reflexivity$1	Is researcher reflexivity demonstrated?<br><br>Specific prompts:<br>? Discussion of relationship between researcher and participants during fieldwork<br>? Demonstration of researcher's influence on stages of research process<br>? Evidence of self-awareness/insight<br>? Documentation of effects of the research on researcher<br>? Evidence of how problems/complications met were dealt with	0 = No<br>1 = Yes
Reflexivity$2	Rate this section<br><br>When Reflexivity$1 = 1, then Reflexivity$2 = 1. When Reflexivity$1 = 0, then Reflexivity$2 = 3. At the reviewer's discretion, Reflexivity$2 may be rated as 2, if the study met most but not all of the prompts.	1 = Strong<br>2 = Moderate<br>3 = Weak
Ethical Dimensions$1	Is there demonstration of	0 = No

For peer review only - http://bmjopen.bmj.com/site/about/guidelines.xhtml

Variable	Question and instruction	Response options
	sensitivity to ethical concerns?<br><br>Specific prompts:<br>? Ethical committee approval granted<br>? Clear commitment to integrity, honesty, transparency, equality and mutual respect in relationships with participants<br>? Evidence of fair dealing with all research participants<br>? Recording of dilemmas met and how resolved in relation to ethical issues<br>? Documentation of how autonomy, consent, confidentiality, anonymity were managed	1 = Yes
Ethical Dimensions$2	Rate this section<br><br>When Dimensions$1 = 1, then Dimensions$2 = 1. When Dimensions$1 = 0, then Dimensions$2 = 3. At the reviewer's discretion, Dimensions$2 may be rated as 2, if the study met most but not all of the prompts.	1 = Strong<br>2 = Moderate<br>3 = Weak
Relevance and Transferability$1	Are relevance and transferability evident?<br><br>Specific prompts:<br>? Sufficient evidence for typicality specificity to be assessed<br>? Analysis interwoven with existing theories and other relevant explanatory literature drawn from similar settings and studies<br>? Discussion of how explanatory propositions/emergent theory may fit other contexts<br>? Limitations/weaknesses of study clearly outlined<br>? Clearly resonates with other knowledge and experience<br>? Results/conclusions obviously supported by evidence<br>? Interpretation plausible and 'makes sense'<br>? Provides new insights and increases understanding	0 = No<br>1 = Yes

For peer review only

Variable	Question and instruction	Response options
	▯ Significance for current policy and practice outlined<br>▯ Assessment of value/empowerment for participants<br>▯ Outlines further directions for investigation<br>▯ Comment on whether aims/purposes of research were achieved	
Relevance and Transferability$2	Rate this section<br><br>Transferability$1 = 1, then Transferability$2 = 1. When Transferability$1 = 0, then Transferability$2 = 3. At the reviewer's discretion, Transferability$2 may be rated as 2, if the study met most but not all of the prompts.	1 = Strong<br>2 = Moderate<br>3 = Weak
Global Rating$1	Based on the final section ratings for scope and purpose, design, sampling strategy, analysis, interpretation, reflexivity, ethical dimensions, and relevance and transferability, what is the global rating for this paper?	1 = Strong (no WEAK ratings)<br>2 = Moderate (one WEAK rating)<br>3 = Weak (two or more WEAK ratings)
Global Rating$2	With both reviewers discussing the ratings, is there a discrepancy between the two reviewers with respect to the following sections: scope and purpose, design, sampling strategy, analysis, interpretation, reflexivity, ethical dimensions, and relevance and transferability	0 = No<br>1 = Yes
Global Rating$3	If YES, indicate the reason for the discrepancy.	1 = Oversight<br>2 = Differences in interpretation of criteria<br>3 = Differences in interpretation of study
Global Rating$4	Final decision of both reviewers	1 = Strong<br>2 = Moderate<br>3 = Weak
Quality assessment (for qualitative studies): Reviewer 2		
Scope and Purpose$4	Does the study have a clear statement of, and rationale for, its research question/aims/purposes?	0 = No<br>1 = Yes

For peer review only

Variable	Question and instruction	Response options
	Specific prompts:<br>? Clarity of focus demonstrated<br>? Explicit purpose given, such as descriptive/explanatory intent, theory building, hypothesis testing<br>? Link between research and existing knowledge demonstrated	
Scope and Purpose$5	Is the study thoroughly contextualized by existing literature?<br><br>Specific prompt:<br>? Evidence of systematic approach to literature review, location of literature to contextualize the findings, or both	0 = No<br>1 = Yes
Scope and Purpose$6	Rate this section.<br><br>When Purpose$4 and Purpose$5 = 1, then Purpose$6 = 1. When Purpose$4 or Purpose$5 = 0, then Purpose$6 = 2. When Purpose$4 and Purpose$5 = 0, then Purpose$6 = 3.	1 = Strong<br>2 = Moderate<br>3 = Weak
Design$4	Is the method/design apparent and consistent with the research intent?<br><br>Specific prompts:<br>? Rationale given for use of qualitative design<br>? Discussion of epistemological/ontological grounding<br>? Rationale explored for specific qualitative method (e.g. ethnography, grounded theory, phenomenology)<br>? Discussion of why particular method chosen is most appropriate/sensitive/relevant for research question/aims<br>? Setting appropriate	0 = No<br>1 = Yes
Design$5	Is the data collection strategy apparent and appropriate?<br><br>Specific prompts:<br>? Were data collection methods appropriate for type of data required and for specific qualitative	0 = No<br>1 = Yes

For peer review only - http://bmjopen.bmj.com/site/about/guidelines.xhtml

Variable	Question and instruction	Response options
	method?<br>Were they likely to capture the complexity/diversity of experience and illuminate context in sufficient detail?<br>Was triangulation of data sources used if appropriate?	
Design$6	Rate this section<br><br>When Design$4 and Design$5 = 1, then Design$6 = 1. When Design$4 or Design$5 = 0, then Design$6 = 2. When Design$4 and Design$5 = 0, then Design$6 = 3.	1 = Strong<br>2 = Moderate<br>3 = Weak
Sampling Strategy$3	Are the sampling and sampling method appropriate?<br><br>Specific prompts:<br>Selection criteria detailed, and description of how sampling was undertaken<br>Justification for sampling strategy given<br>Thickness of description likely to be achieved from sampling<br>Any disparity between planned and actual sample explained	0 = No<br>1 = Yes
Sampling Strategy$4	Rate this section<br><br>When Strategy$3 = 1, then Strategy$4 = 1. When Strategy$3 = 0, then Strategy$4 = 3. At the reviewer's discretion, Strategy$4 may be rated as 2, if the study met most but not all of the prompts.	1 = Strong<br>2 = Moderate<br>3 = Weak
Analysis$3	Is the analytical approach appropriate?<br><br>Specific prompts:<br>Approach made explicit (e.g. Thematic distillation, constant comparative method, grounded theory)<br>Was it appropriate for the qualitative method chosen?<br>Was data managed by software package or by hand and why?<br>Discussion of how coding systems/conceptual frameworks evolved	0 = No<br>1 = Yes

For peer review only

For peer review only - http://bmjopen.bmj.com/site/about/guidelines.xhtml

Variable	Question and instruction	Response options
	▯ How was context of data retained during analysis<br>▯ Evidence that the subjective meanings of participants were portrayed<br>▯ Evidence of more than one researcher involved in stages if appropriate to epistemological/theoretical stance<br>▯ Did research participants have any involvement in analysis (e.g. member checking)<br>▯ Evidence provided that data reached saturation or discussion/rationale if it did not<br>▯ Evidence that deviant data was sought, or discussion/rationale if it was not	
Analysis$4	Rate this section<br><br>When Analysis$3 = 1, then Analysis$4 = 1. When Analysis$3 = 0, then Analysis$4 = 3. At the reviewer's discretion, Analysis$4 may be rated as 2, if the study met most but not all of the prompts.	1 = Strong<br>2 = Moderate<br>3 = Weak
Interpretation$5	Is the context described and taken account of in the interpretation?<br><br>Specific prompts:<br>▯ Description of social/physical and interpersonal contexts of data collection<br>▯ Evidence that researcher spent time 'dwelling with the data', interrogating it for competing/alternative explanations of phenomena	0 = No<br>1 = Yes
Interpretation$6	Is a clear audit trail given?<br><br>Specific prompt:<br>▯ Sufficient discussion of research processes such that others can follow the 'decision trail'	0 = No<br>1 = Yes
Interpretation$7	Are data used to support interpretation?<br><br>Specific prompts:<br>▯ Extensive use of field notes entries/verbatim interview quotes	0 = No<br>1 = Yes

For peer review only

Variable	Question and instruction	Response options
	in discussion of findings<br>? Clear exposition of how interpretation led to conclusions	
Interpretation$8	Rate this section<br><br>When Interpretation$5, Interpretation$6, and Interpretation$7 = 1, then Interpretation$8 = 1. When one response to Interpretation$5—Interpretation$7 = 0, then Interpretation$8 = 2. When two or more responses to Interpretation$5—Interpretation$7 = 0, then Interpretation$8 = 3.	1 = Strong<br>2 = Moderate<br>3 = Weak
Reflexivity$3	Is researcher reflexivity demonstrated?<br><br>Specific prompts:<br>? Discussion of relationship between researcher and participants during fieldwork<br>? Demonstration of researcher's influence on stages of research process<br>? Evidence of self-awareness/insight<br>? Documentation of effects of the research on researcher<br>? Evidence of how problems/complications met were dealt with	0 = No<br>1 = Yes
Reflexivity$4	Rate this section<br><br>When Reflexivity$3 = 1, then Reflexivity$4 = 1. When Reflexivity$3 = 0, then Reflexivity$4 = 3. At the reviewer's discretion, Reflexivity$4 may be rated as 2, if the study met most but not all of the prompts.	1 = Strong<br>2 = Moderate<br>3 = Weak
Ethical Dimensions$3	Is there demonstration of sensitivity to ethical concerns?<br><br>Specific prompts:<br>? Ethical committee approval granted<br>? Clear commitment to integrity, honesty, transparency, equality and mutual respect in relationships	0 = No<br>1 = Yes

For peer review only

Variable	Question and instruction	Response options
	with participants<br>▯ Evidence of fair dealing with all research participants<br>▯ Recording of dilemmas met and how resolved in relation to ethical issues<br>▯ Documentation of how autonomy, consent, confidentiality, anonymity were managed	
Ethical Dimensions$4	Rate this section<br><br>When Dimensions$3 = 1, then Dimensions$4 = 1. When Dimensions$3 = 0, then Dimensions$4 = 3. At the reviewer's discretion, Dimensions$4 may be rated as 2, if the study met most but not all of the prompts.	1 = Strong<br>2 = Moderate<br>3 = Weak
Relevance and Transferability$3	Are relevance and transferability evident?<br><br>Specific prompts:<br>▯ Sufficient evidence for typicality specificity to be assessed<br>▯ Analysis interwoven with existing theories and other relevant explanatory literature drawn from similar settings and studies<br>▯ Discussion of how explanatory propositions/emergent theory may fit other contexts<br>▯ Limitations/weaknesses of study clearly outlined<br>▯ Clearly resonates with other knowledge and experience<br>▯ Results/conclusions obviously supported by evidence<br>▯ Interpretation plausible and 'makes sense'<br>▯ Provides new insights and increases understanding<br>▯ Significance for current policy and practice outlined<br>▯ Assessment of value/empowerment for participants<br>▯ Outlines further directions for investigation<br>▯ Comment on whether	0 = No<br>1 = Yes

For peer review only - http://bmjopen.bmj.com/site/about/guidelines.xhtml

Variable	Question and instruction	Response options
	aims/purposes of research were achieved	
Relevance and Transferability$4	Rate this section<br><br>Transferability$3 = 1, then Transferability$4 = 1. When Transferability$3 = 0, then Transferability$4 = 3. At the reviewer's discretion, Transferability$4 may be rated as 2, if the study met most but not all of the prompts.	1 = Strong<br>2 = Moderate<br>3 = Weak
Global Rating$5	Based on the final section ratings for scope and purpose, design, sampling strategy, analysis, interpretation, reflexivity, ethical dimensions, and relevance and transferability, what is the global rating for this paper?	1 = Strong (no WEAK ratings)<br>2 = Moderate (one WEAK rating)<br>3 = Weak (two or more WEAK ratings)

For peer review only

For peer review only - http://bmjopen.bmj.com/site/about/guidelines.xhtml

PRISMA-P (Preferred Reporting Items for Systematic review and Meta-Analysis Protocols) 2015 checklist: recommended items to address in a systematic review protocol*

Section and topic	Item	Checklist item	Reported on page
**ADMINISTRATIVE INFORMATION**			
Title:			
Identification	1a	Identify the report as a protocol of a systematic review	1
Update	1b	If the protocol is for an update of a previous systematic review, identify as such	Not applicable
Registration	2	If registered, provide the name of the registry (such as PROSPERO) and registration number	13
Authors:			
Contact	3a	Provide name, institutional affiliation, e-mail address of all protocol authors; provide physical mailing address of corresponding author	1
Contributions	3b	Describe contributions of protocol authors and identify the guarantor of the review	13
Amendments	4	If the protocol represents an amendment of a previously completed or published protocol, identify as such and list changes; otherwise, state plan for documenting important protocol amendments	13
Support:			
Sources	5a	Indicate sources of financial or other support for the review	13
Sponsor	5b	Provide name for the review funder and/or sponsor	13
Role of sponsor or funder	5c	Describe roles of funder(s), sponsor(s), and/or institution(s), if any, in developing the protocol	13
**INTRODUCTION**			
Rationale	6	Describe the rationale for the review in the context of what is already known	4
Objectives	7	Provide an explicit statement of the question(s) the review will address with reference to participants, interventions, comparators, and outcomes (PICO)	4-5
**METHODS**			
Eligibility criteria	8	Specify the study characteristics (such as PICO, study design, setting, time frame) and report characteristics (such as years considered, language, publication status) to be used as criteria for eligibility for the review	5-6

For peer review only

Information sources	9	Describe all intended information sources (such as electronic databases, contact with study authors, trial registers or other grey literature sources) with planned dates of coverage	5-6
Search strategy	10	Present draft of search strategy to be used for at least one electronic database, including planned limits, such that it could be repeated	9-12
Study records:			
Data management	11a	Describe the mechanism(s) that will be used to manage records and data throughout the review	8
Selection process	11b	State the process that will be used for selecting studies (such as two independent reviewers) through each phase of the review (that is, screening, eligibility and inclusion in meta-analysis)	8
Data collection process	11c	Describe planned method of extracting data from reports (such as piloting forms, done independently, in duplicate), any processes for obtaining and confirming data from investigators	8
Data items	12	List and define all variables for which data will be sought (such as PICO items, funding sources), any pre-planned data assumptions and simplifications	8, Annexes A-B
Outcomes and prioritization	13	List and define all outcomes for which data will be sought, including prioritization of main and additional outcomes, with rationale	6, 8, Annexes A-B
Risk of bias in individual studies	14	Describe anticipated methods for assessing risk of bias of individual studies, including whether this will be done at the outcome or study level, or both; state how this information will be used in data synthesis	8-9
Data synthesis	15a	Describe criteria under which study data will be quantitatively synthesised	9
	15b	If data are appropriate for quantitative synthesis, describe planned summary measures, methods of handling data and methods of combining data from studies, including any planned exploration of consistency (such as I^2, Kendall's τ)	9
	15c	Describe any proposed additional analyses (such as sensitivity or subgroup analyses, meta-regression)	9
	15d	If quantitative synthesis is not appropriate, describe the type of summary planned	9
Meta-bias(es)	16	Specify any planned assessment of meta-bias(es) (such as publication bias across studies, selective reporting within studies)	9
Confidence in cumulative evidence	17	Describe how the strength of the body of evidence will be assessed (such as GRADE)	8-9

*** It is strongly recommended that this checklist be read in conjunction with the PRISMA-P Explanation and Elaboration (cite when available) for important clarification on the items. Amendments to a review protocol should be tracked and dated. The copyright for PRISMA-P (including checklist) is held by the PRISMA-P Group and is distributed under a Creative Commons Attribution Licence 4.0.**

From: Shamseer L, Moher D, Clarke M, Ghersi D, Liberati A, Petticrew M, Shekelle P, Stewart L, PRISMA-P Group. Preferred reporting items for systematic review and meta-analysis protocols (PRISMA-P) 2015: elaboration and explanation. BMJ. 2015 Jan 2;349(jan02 1):g7647.

For peer review only
